# Prevalence and Disease Expression of Pathogenic and Likely Pathogenic Variants Associated With Inherited Cardiomyopathies in the General Population

**DOI:** 10.1161/CIRCGEN.122.003704

**Published:** 2022-10-20

**Authors:** Mimount Bourfiss, Marion van Vugt, Abdulrahman I. Alasiri, Bram Ruijsink, Jessica van Setten, A. Floriaan Schmidt, Dennis Dooijes, Esther Puyol-Antón, Birgitta K. Velthuis, J. Peter van Tintelen, Anneline S.J.M. te Riele, Annette F. Baas, Folkert W. Asselbergs

**Affiliations:** 1 Dept of Cardiology, Univ Medical Center Utrecht, Utrecht Univ, Utrecht, the Netherlands (M.B., M.v.V., A.I.A., A.S.J.M.t.R., B.R., J.v.S., A.F.S., F.W.A.).; 2 School of Biomedical Engineering & Imaging Sciences, King’s College London, London, United Kingdom (B.R., E.P.-A.).; 3 Faculty of Population Health Sciences Institute of Cardiovascular Science, London, London, United Kingdom (A.F.S., F.W.A.).; 4 Dept of Genetics, Univ Medical Center Utrecht, Utrecht Univ, Utrecht, the Netherlands (D.D., J.P.v.T., A.F.B.).; 5 Dept of Radiology, Univ Medical Center Utrecht, Utrecht Univ, Utrecht, the Netherlands (B.K.V.).; 6 Netherlands Heart Institute, Utrecht, the Netherlands (A.S.J.M.t.R).; 7 Health Data Research UK & Institute of Health Informatics, Univ College London, London, United Kingdom (F.W.A.).

**Keywords:** whole exome sequencing, genetics, arrhythmogenic right ventricular cardiomyopathy, dilated cardiomyopathy, hypertrophic cardiomyopathy

## Abstract

Pathogenic and likely pathogenic variants associated with arrhythmogenic right ventricular cardiomyopathy (ARVC), dilated cardiomyopathy (DCM), and hypertrophic cardiomyopathy (HCM) are recommended to be reported as secondary findings in genome sequencing studies. This provides opportunities for early diagnosis, but also fuels uncertainty in variant carriers (G+), since disease penetrance is incomplete. We assessed the prevalence and disease expression of G+ in the general population.

**Methods:**

We identified pathogenic and likely pathogenic variants associated with ARVC, DCM and/or HCM in 200 643 UK Biobank individuals, who underwent whole exome sequencing. We calculated the prevalence of G+ and analyzed the frequency of cardiomyopathy/heart failure diagnosis. In undiagnosed individuals, we analyzed early signs of disease expression using available electrocardiography and cardiac magnetic resonance imaging data.

**Results:**

We found a prevalence of 1:578, 1:251, and 1:149 for pathogenic and likely pathogenic variants associated with ARVC, DCM and HCM respectively. Compared with controls, cardiovascular mortality was higher in DCM G+ (odds ratio 1.67 [95% CI 1.04; 2.59], *P*=0.030), but similar in ARVC and HCM G+ (*P*≥0.100). Cardiomyopathy or heart failure diagnosis were more frequent in DCM G+ (odds ratio 3.66 [95% CI 2.24; 5.81], *P*=4.9×10^−7^) and HCM G+ (odds ratio 3.03 [95% CI 1.98; 4.56], *P*=5.8×10^−7^), but comparable in ARVC G+ (*P*=0.172). In contrast, ARVC G+ had more ventricular arrhythmias (*P*=3.3×10^−4^). In undiagnosed individuals, left ventricular ejection fraction was reduced in DCM G+ (*P*=0.009).

**Conclusions:**

In the general population, pathogenic and likely pathogenic variants associated with ARVC, DCM, or HCM are not uncommon. Although G+ have increased mortality and morbidity, disease penetrance in these carriers from the general population remains low (1.2–3.1%). Follow-up decisions in case of incidental findings should not be based solely on a variant, but on multiple factors, including family history and disease expression.

## Introduction

The major inherited cardiomyopathies arrhythmogenic right ventricular cardiomyopathy (ARVC), dilated cardiomyopathy (DCM), and hypertrophic cardiomyopathy (HCM) are characterized by ventricular dysfunction and ventricular arrhythmias that can lead to progressive heart failure and sudden cardiac death.^1^ ARVC is mainly caused by pathogenic variants in desmosomal genes, whereas DCM and HCM are mainly caused by sarcomeric gene variants.^2^ These cardiomyopathies are typically inherited in an autosomal dominant fashion with incomplete penetrance and variable expressivity. As such, phenotypic expression may vary greatly, even among family members or individuals carrying the same pathogenic variant.

With the implementation of next-generation sequencing, genetic testing has become an important part of routine clinical care in the diagnosis of inherited cardiomyopathies.^3^ Technical advances and commercial availability of next-generation sequencing have led to more affordable and accessible genetic testing. The American College of Medical Genetics and Genomics has developed recommendations for the reporting of incidental or secondary findings unrelated to the test indication.^4^ In this framework, variants in genes associated with ARVC, DCM, and HCM are recommended to be reported as secondary findings from clinical exome and other genome sequencing tests.^4^ Although this offers the potential to prevent morbidity and mortality of heart failure and sudden cardiac death by early treatment, it also fuels uncertainty in carriers of likely pathogenic or pathogenic variants (G+) and their family members, since factors that influence disease penetrance in the general population are largely unknown. More knowledge about disease penetrance of these variants in an unselected population cohort is needed to determine screening protocols in these individuals.

**Figure 1.**
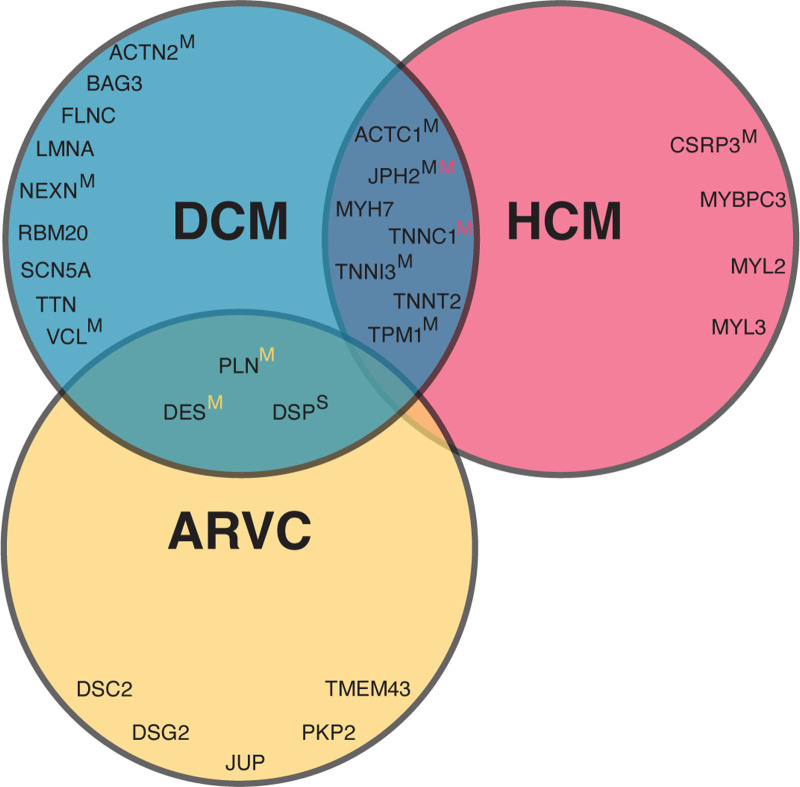
**Included curated genes per cardiomyopathy.** The Venn diagram of curated genes included in this study shows the overlap in genes per cardiomyopathy. Unless otherwise indicated, pathogenicity of genes are classified as definitive. If a superscript S or M is given, genes are classified as having a strong or moderate pathogenicity, respectively. In the overlapping circles, yellow, black, and red colors refer to ARVC, DCM, and HCM, respectively. Table S1 gives an overview of the included genes and pathogenicity classification per gene and abbreviation per gene. ARVC indicates arrhythmogenic right ventricular cardiomyopathy; DCM, dilated cardiomyopathy; HCM, hypertrophic cardiomyopathy.

In this study, we aimed to assess the prevalence of pathogenic and likely pathogenic variants in the general population using a set of recently curated genes for ARVC,^5^ DCM,^6^ and HCM^7^ in 2 (inter)national databases^8^ (see Figure 1 and Table S1). In order to do so, we leveraged data from the UK Biobank (UKB), a population-based cohort with whole exome sequencing data available of 200 643 individuals.^9^ Furthermore, we looked into the UKB-reported phenotypical characteristics of these G+ and assessed the occurrence of early signs of disease in undiagnosed G+ using available electrocardiography and cardiac magnetic resonance (CMR) imaging data.

## Methods

Ethics approval for the UKB study was obtained from the North West Centre for Research Ethics Committee (11/NW/0382), and all participants provided informed consent. All data and materials have been made publicly available on Github and can be accessed at https://github.com/CirculatoryHealth/Inherited-cardiomyopathies. Full methods are available in the Supplemental Material. Disease definitions are given in Table S2.

## Results

### Population Characteristics

**Figure 2.**
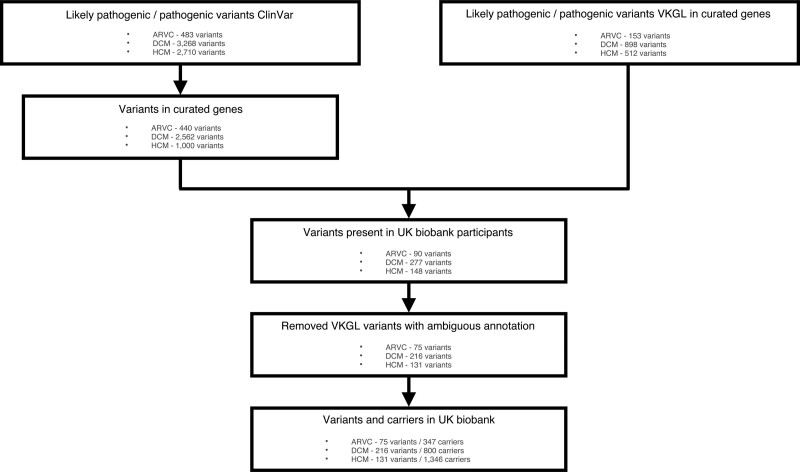
**Flowchart inclusion of variants.** Flowchart depicting the inclusion of pathogenic and likely pathogenic variants associated with arrhythmogenic cardiomyopathy, dilated cardiomyopathy, and hypertrophic cardiomyopathy from the ClinVar^8^ and VKGL database. ARVC indicates arrhythmogenic right ventricular cardiomyopathy; DCM, dilated cardiomyopathy; HCM, hypertrophic cardiomyopathy; and VKGL, Vereniging Klinische Genetische Laboratoriumdiagnostiek.

We identified 2207/200 643 unique G+ individuals from a total of 2493 included individuals (89%) (see Figure 2) classified as (1) ARVC G+ (n=347, 54% women, median age of 57 [50–64] years); (2) DCM G+ (n=800, 56% women, median age of 58 [51–64] years); and (3) HCM G+ (n=1346, 54% women, median age of 56 [49–63] years). The matched control group consisted of 9972 individuals (55% women, median age of 57 [49–63] years). Table 1 and Table S3 show the baseline characteristics of the included individuals. The majority of G+ were of White ethnicity (ARVC 90%, DCM 96%, HCM 75%), followed by Asian (ARVC 3%, DCM 1%, HCM 19%) and Black ethnicity (ARVC 2%, DCM 2% and HCM 2%). This is comparable to what is observed in the UKB, where the majority is of White ethnicity (94%), followed by Asian (2%) and Black ethnicity (2%).

**Table 1.**
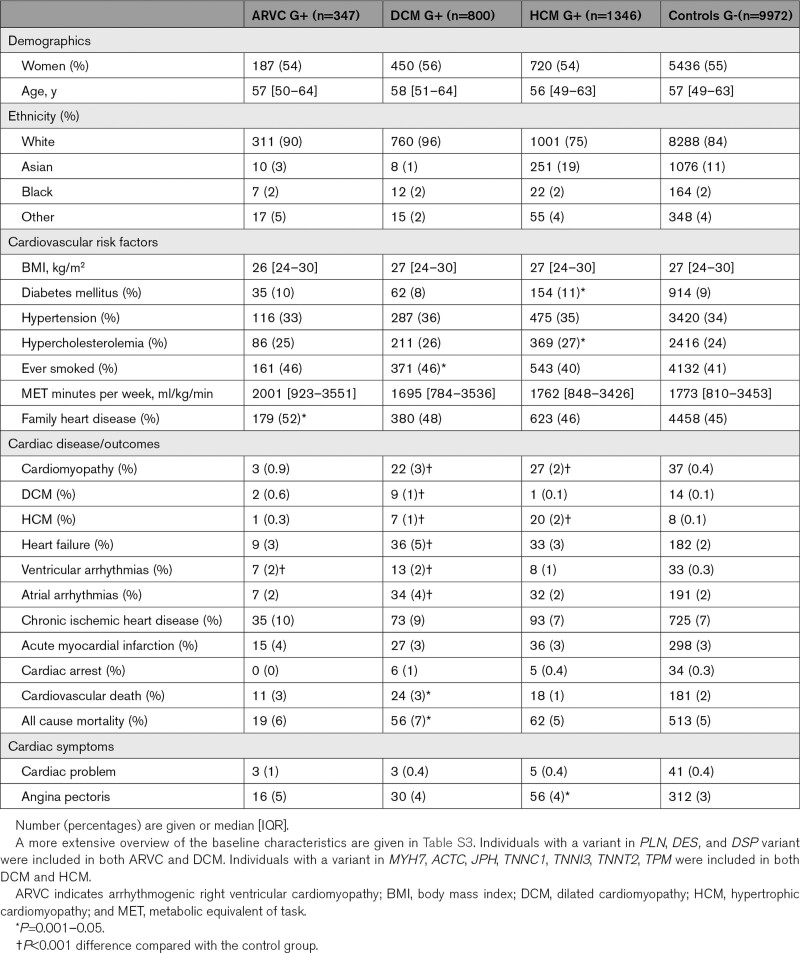
Baseline Characteristics of Variant Carriers and Controls

### Genotypic Characteristics of Pathogenic and Likely Pathogenic Variant Carriers

#### Prevalence of Pathogenic and Likely Pathogenic Variants in the General Population

**Figure 3.**
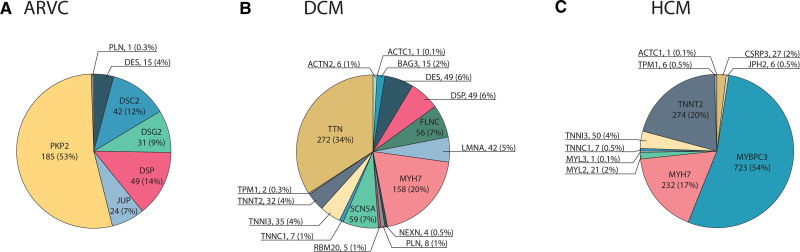
**Distribution of genes per cardiomyopathy.** Piecharts with the distribution of curated genes for (**A**) arrhythmogenic right ventricular cardiomyopathy (ARVC); (**B**) dilated cardiomyopathy (DCM); (**C**) hypertrophic cardiomyopathy (HCM). Abbreviations of the different genes are given in Table S3. G+ indicates pathogenic variant carrier.

We found a prevalence of 1 ARVC G+ in 578 people in the general population (1:578 [1:521; 1:644]) and identified 75 variants of the 593 (13%) pathogenic and likely pathogenic variants described in ClinVar and VKGL: 13 missense and 62 loss of function (Table S4). As shown in Figure 3, most ARVC G+ harbored a pathogenic variant in *PKP2* (n=185, 53%), followed by Desmoplakin (*DSP*; n=49, 14%), *DSC2* (n=42, 12%), *DSG2* (n=31, 9%), *JUP* (n=24, 7%), Desmin (*DES*; n=15, 4%), and *PLN* (n=1, 0.3%).

We found a prevalence of 1:251 [1:234; 1:269] DCM G+ and identified 216 of 3460 (6%) pathogenic and likely pathogenic variants described in ClinVar and VKGL: 80 missense and 136 loss of function (Table S4). Variants in *TTN* (n=272, 34%) and *MYH7* (n=158, 20%) were most prevalent among DCM G+, followed by *SCN5A* (n=59, 7%), *FLNC* (n=56, 7%), *DSP* (n=49, 6%), *DES* (n=49, 6%), Lamin A/C (*LMNA*; n=42, 5%), *TNNI3* (n=35, 4%), and *TNNT2* (n=32, 4%). Eight more genes with a frequency of <3% were identified: *BAG3*, *PLN*, *TNNC1, ACTN2*, *RBM20, NEXN, TPM1*, and *ACTC1* (Figure 3).

We found a prevalence of 1:149 [1:141; 1:157] HCM G+ and identified 131 of 1512 (9%) pathogenic and likely pathogenic variants from ClinVar and VKGL: 98 missense and 23 loss of function (Table S4). Most individuals carried a pathogenic and likely pathogenic variant in *MYBPC3* (n=723, 54%), followed by *TNNT2* (n=274, 20%), *MYH7* (n=232, 17%), and *TNNI3* (n=50, 4%). A frequency of <3% was found in *CSRP3, MYL2, TNNC1, JPH2, TPM1, ACTC1*, and *MYL3* (Figure 3). Interestingly, a variant in *TNNT2* (c.862C>T p.Arg288Cys) affected 242 individuals (18%). Four of these carriers were diagnosed with heart failure, of whom 1 also with HCM. All four heart failure patients also suffered from chronic ischemic heart disease. Furthermore, a variant in *MYBPC3* (c.3628-41_3628-17del) was mainly seen in individuals with an Asian ethnicity (n=237, 18% of the total HCM G+). Four of these individuals were diagnosed with heart failure, of whom 2 also had coronary artery disease and 1 was diagnosed with DCM; however, none were diagnosed with HCM. When excluding these 2 variants, we found a HCM G+ prevalence of 1:250 [1:234; 1:269]. *MYBPC3* remained the most prevalent gene (52%), whereas the *TNNT2* frequency decreased to 4%.

The prevalence of G+ per gene for all cardiomyopathies is depicted in Table S5.

#### Overlapping Variants and Individuals

Some pathogenic and likely pathogenic variants were identified in multiple cardiomyopathy subtypes. First, 26 variants were described in both ARVC and DCM, affecting 53 individuals. Most of these variants (n=20/26 variants, 77%) were found in *DSP* (n=37 individuals, 70%), of whom 1 individual (3%) had heart failure and 1 (3%) was diagnosed with a cardiomyopathy. Five variants out of 26 (19%) were found in *DES* (n=15 individuals, 28%) of whom 2 individuals (13%) had heart failure, and 1 was diagnosed with both DCM and HCM. One variant out of 26 (4%) was found in *PLN* (c.26G>A; p.Arg9His, NM_002667.5) in 1 individual (2%) who was not diagnosed with a cardiomyopathy or heart failure.

Second, 52 variants were described in DCM and HCM, affecting 232 individuals. Most of these variants (n=33/52 variants, 63%) were found in *MYH7* (n=158 individuals, 68%), followed by 10 variants (19%) in *TNNT2* (n=29 individuals, 13%), 6 variants (12%) in *TNNI3* (n=35 individuals, 15%), and 1 (2%) variant in *TNNC1*, *ACTC1* as well as *TPM1*. In this group of 232 individuals, 9 (4%) individuals had a cardiomyopathy or heart failure diagnosis, of whom 5 were diagnosed with HCM and none with DCM.

Furthermore, 3 individuals carried 2 pathogenic variants. Individual 1 was a 65-year-old male, carrying variants in *MYBPC3* (c.3628-41_3628-17del, NM_000256.3) and *TNNT2* (c.460C>T; p.Arg154Trp, NM_001276345.2) and was diagnosed with heart failure, with underlying chronic ischemic heart disease. Individual 2 was a 65-year-old male, carrying variants in *FLNC* (c.7450G>A; p.Gly2484Ser, NM_001458.5) and *PKP2* (c.1867G>T; p.Glu623Ter, NM_001005242.3) and was therefore included in both the ARVC as well as the DCM G+ group. Individual 3 was a 64-year-old man, carrying variants in *MYBPC3* (c.1504C>T; p.Arg502Trp, NM_000256.3) and *MYH7* (c.5655G>A; p.Ala1885=, NM_000257.4). Individuals 2 and 3 were not diagnosed with a cardiomyopathy or heart failure and none had CMR data available.

Table S6 shows the prevalence of the cardiomyopathy variants, with and without the inclusion of overlapping variants.

### Phenotypic Characteristics of Pathogenic and Likely Pathogenic Variant Carriers

#### Cardiovascular Risk Factors

Hypertension, body mass index, and level of activity in metabolic equivalent of task minutes per week were comparable between G− and G+ for all cardiomyopathies (*P*≥0.055; Table 1 and Table S7). Diabetes was more prevalent in G+ HCM (9.2% (G−) versus 11.4% (G+), *P*=0.008), while smoking was more prevalent in DCM G+ (41.4% versus 46.4%, *P=*0.007) (Table S8).

#### Cardiovascular Disease

**Figure 4.**
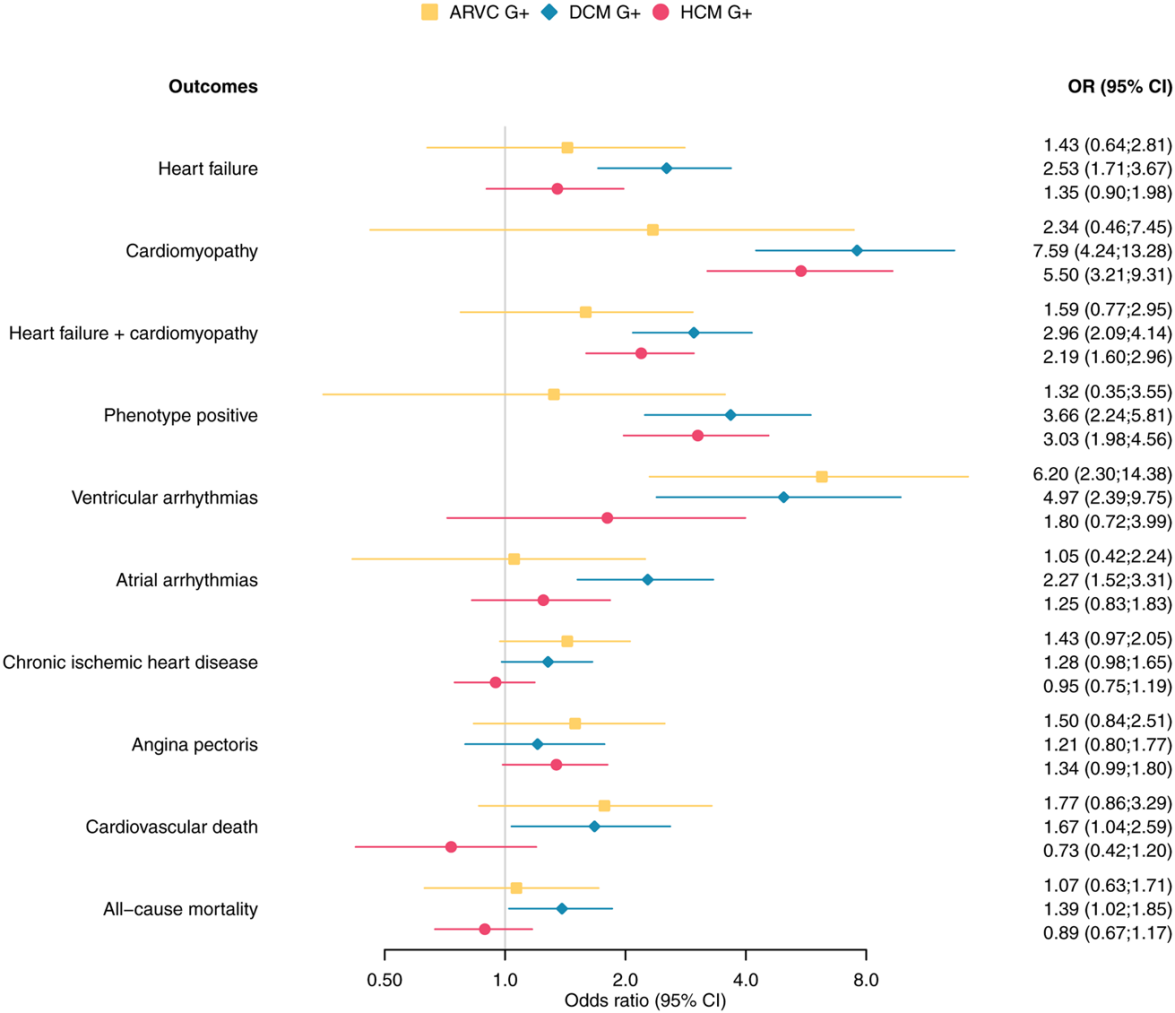
**Forest plot cardiac outcomes stratified per inherited cardiomyopathy. Odds ratios and 95% confidence interval are given for the associations between cardiac outcomes and ARVC, DCM, or HCM pathogenic variant carriers.** ARVC indicates arrhythmogenic right ventricular cardiomyopathy; DCM, dilated cardiomyopathy; G+, pathogenic variant carrier; and HCM= hypertrophic cardiomyopathy.

As seen in Figure 4 and Table S8, compared with G−, cardiomyopathy/heart failure without previous ischemic heart disease (P+, phenotype positive) was more often diagnosed in DCM G+ (odds ratio [OR] 3.66 [95% CI 2.24; 5.81], *P=*4.9×10^−^^−7^) and HCM G+ (OR 3.03 [95% CI 1.98; 4.56], *P=*5.8×10^−^^−7^). Among DCM G+, 25 individuals (3.1%, genes: 8 *MYH7*, 8 *TTN*, 2 *BAG3*, 2 *DSP*, 2 *FLNC*, 1 *DES*, 1 *SCN5A*, and 1 *TNNT2*) were P+. Within HCM G+, 32 individuals (4.0%, genes: 20 *MYBPC3*, 10 *MYH7*, 1 *TNNI3* and 1 *TNNT2*) were P+. For ARVC G+, 4 individuals (1.2%, genes: 2 *DSP*, 1 *DES* and 1 *PKP2*) were P+, being comparable to G- controls (87 subjects, 0.8%).

Ventricular arrhythmias occurred more often in G+ compared with G−, reaching statistical significance for ARVC (OR 6.20 [95% CI 2.30;14.38], *P=*3.3×10^-4^) and DCM (OR 4.97 [95% CI 2.39; 9.75], *P=*1.9×10^−^^−5^). Atrial arrhythmias were more prevalent among DCM G+ (OR 2.27 [95% CI 1.52;3.31], *P=*8.2×10^-5^). Finally, all-cause mortality (OR 1.39 [95% CI 1.02; 1.85], *P=*0.032) and death due to a cardiovascular cause were more prevalent in DCM G+ (OR 1.67 [95% CI 1.04; 2.59], *P=*0.030) but did not reach statistical significance for ARVC G+ and HCM G+ (*P*≥0.100). Figure S1 depicts the overlap in cardiomyopathy, heart failure, ventricular arrhythmia, and ischemic heart disease diagnosis. Figure S2 depicts the forest plots when excluding the more prevalent *TNNT2* and *MYBPC3* variants in HCM G+ individuals and Figure S3 shows the association between different outcomes stratified by genes for each cardiomyopathy.

### Deep Phenotyping of Undiagnosed Pathogenic Variant Carriers

Next, we set out to study early signs of disease in G+ without a cardiomyopathy/heart failure diagnosis (P−) using registered ICD-10 codes, self-reported cardiac symptoms, and abnormal electrocardiography and CMR values.

#### Diagnosis and Symptoms

Ventricular arrhythmias were more prevalent in ARVC G+P− (OR 5.85 [95% CI 1.98;14.40], *P=*0.001) and DCM G+P− (OR 3.43 [95% CI 1.35;7.68], *P=*0.005) but not in HCM G+P− (OR 1.01 [95% CI 0.26;2.86], *P=*1.000) compared with G−P− controls. Also, atrial arrhythmias (OR 2.12 [95% CI 1.36;3.19], *P=*7.9×10^−^^−4^) were more frequent in DCM G+P− compared with G−P− controls. Finally, angina pectoris occurred more often in HCM G+P− (OR 1.38 [95% CI 1.01;1.85], *P=*0.038), but not in ARVC G+P− and DCM G+P− (*P*≥0.117; Table S7).

#### Electrocardiography

In total, 231 of 2207 G+P− and 1058 of 9885 G−P− had various electrocardiography variables available. None of these electrocardiography variables differed significantly between all undiagnosed G+ and control individuals (Table S8).

#### Cardiac Magnetic Resonance Imaging

CMR data were available in 225 G+P− of the 2207 unique G+P− individuals: n=33 for ARVC G+P−, n=87 for DCM G+P− and n=130 for HCM G+P−) and 986 of the 9885 G−P− controls. As shown in Table S9, G+P− and G−P− individuals with CMR data available were mostly comparable. Only smoking was more prevalent among DCM G+P− compared with G−P− controls (OR 1.59 [95%CI 1.00; 2.53], *P=*0.041). Outliers were observed in G−P− controls: 4 individuals with a median age of 64 [60–67] years had a left ventricular ejection fraction (LVEF) below 40% and 3 of them were diagnosed with hypertension and 2 with acute myocardial infarction in the past. In addition, 2 individuals with an age of 42 and 52 years old had an RVEF below 40%. They did not suffer from hypertension and did not have any cardiac diagnosis.

**Figure 5.**
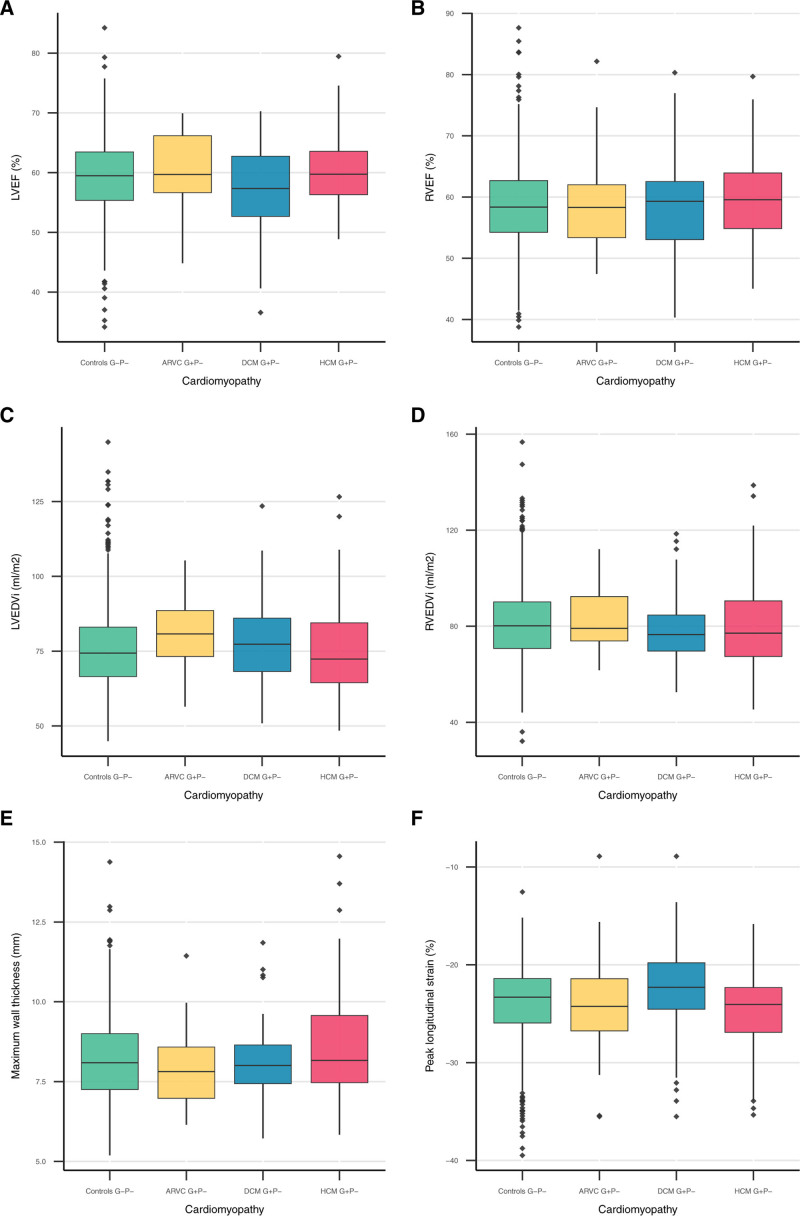
**CMR parameters stratified per inherited cardiomyopathy.** Boxplots of the following CMR parameters: (**A**) LVEF; (**B**) RVEF; (**C**) LVEDVi; (**D**) RVEDVi; (**E**) maximum wall thickness; and (**F**) peak longitudinal strain. Boxplots show the summary statistics of CMR parameters stratified by controls and individuals with a pathogenic variant associated with ARVC, DCM, or HCM. Displayed summary statistics include the median, first and third quartile (lower and upper box edges), and the whiskers represent values within 1.5 times the interquartile range from the box edges. ARVC indicates arrhythmogenic right ventricular cardiomyopathy; DCM, dilated cardiomyopathy; EDVi, body surface area corrected end-diastolic volume; EF, ejection fraction; G+, pathogenic variant carrier; HCM, hypertrophic cardiomyopathy; LV, left ventricular; RV, right ventricular.

As shown in Figure 5 and Table S8, all Right ventricular (*P*≥0.546) and Left ventricular (p≥0.052) functional and structural parameters in ARVC G+P− were comparable to G−P− controls. Three ARVC G+P− individuals had an RVEDV corrected for body surface area (RVEDVi) between 100 and 110 mL/m^−2^ for men or between 90 and 100 mL/m^−2^ for women, meeting the minor CMR task force criteria if wall motion abnormalities were present, and 2 ARVC G+P− individuals had an RVEDVi larger than 110 mL/m^−2^ for men or 100 mL/m^−2^ for women, meeting the major CMR task force criteria.^10^ In addition, ARVC G+P− had reduced inferior and posterolateral wall thickness compared with controls (*P*≤0.035).

Overall, DCM G+P− and G−P− controls had comparable Right ventricular functional and structural measures (p≥0.048). However, DCM G+P− had lower LVEF (57.3% [52.6, 62.8] versus 59.5% [55.3, 63.5] versus, *P=*0.009) and less negative Left ventricular peak longitudinal strain (−22.3% [−24.6, −19.86] versus −23.3% [−26.0, −21.4], *P=*0.009). Although LVEDVi was not significantly increased in DCM G+P−, the LVEDV/RVEDV ratio (0.9 [0.9, 1.0] versus 1.0 [0.9, 1.1], *P=*8.2×10^−^^−4^) and LVESVi (30.0 ml/m^−2^ [25.1, 35.7] versus 31.7 ml/m^−2^ [26.2, 39.8], *P=*0.032) were increased. Six individuals had an LVEF below 45%, but none of the individuals met the Henry criteria for DCM (LVEF below 45% and LVEDVi 2 times the normal SD).^11^

For HCM G+P−, most Right ventricular and Left ventricular functional and dimension parameters were comparable to G−P− controls (*P*≥0.051). Only RVEF was higher than controls (58.4% [54.2, 62.7] versus 59.6% [54.8, 64.0], *P=*0.025). Importantly, wall thickness was not significantly different between HCM G+P− without a cardiomyopathy/heart failure diagnosis and G−P− (*P*≥0.160). None of the G+P− individuals met HCM criteria^12^ of ≥15 mm wall thickness, but 2 individuals met the criteria for limited hypertrophy (13–15 mm) in the presence of a positive genetic test.^12^

Figure S4 shows a summary of all the differences tested.

#### Exclusion of the More Prevalent TNNT2 and MYBPC3 Variants

When excluding the more prevalent *TNNT2* and *MYBPC3* variants in HCM G+P− individuals, the occurrence of ventricular arrhythmias (OR 1.72 [95% CI 0.44;4.89], *P=*0.306) and atrial arrhythmias (OR 1.43 [95% CI 0.84;2.32], *P=*0.156) was comparable to G−P− controls. However, the maximum wall thickness (8.47 mm [7.59, 9.94] versus 8.09 mm [7.24, 9.01], *P=*0.008) and basal anterior wall thickness (7.93 mm [6.97, 9.11] versus 7.65 mm [6.81, 8.49], *P*=0.029) were significantly increased in HCM G+P− compared with controls (Table S8). Two individuals had a maximum wall thickness between 13 and 15 mm.

## Discussion

In this study, we leveraged the largest European population database, including whole exome sequencing and phenotype data to evaluate the prevalence and penetrance of previously reported pathogenic and likely pathogenic variants associated with ARVC, DCM, and HCM. Our study has several interesting findings. First, we found a prevalence of 1:578, 1:251, and 1:149 for variants previously associated with ARVC, DCM, and HCM, respectively. Second, 1.2% of ARVC G+, 3.1% of DCM G+ and 2.6% of HCM G+ were diagnosed with a cardiomyopathy or heart failure without previous chronic ischemic heart disease. Finally, 3.2% of the undiagnosed ARVC G+, 1.8% of the undiagnosed DCM G+, and 0.5% of the undiagnosed HCM G+ reported ventricular arrhythmias or had CMR abnormalities. These results confirm the low disease penetrance in G+ in the general population.

### Prevalence of Pathogenic and Likely Pathogenic Variant Carriers in the General Population

Since rare genetic variants are the major cause of inherited cardiomyopathies, a large dataset is needed to accurately identify the population prevalence of these variants. Prevalence of pathogenic variants in populations has been the focus of several previous studies^4,13–15^; however, they were mostly limited by the number of included individuals. At the time of analysis, we had access to an unprecedented number of 200 643 individuals.

Previously reported prevalence of ARVC G+ in the general population ranges between 1:143 and 1:1706.^13–15^ This variability is likely to be explained by heterogeneity in study populations and definitions of variant pathogenicity. For example, many previous studies did not include all 8 curated genes with strong or moderate disease-gene association but also marked other genes (eg, *TGFB3*) with only limited evidence as associated with ARVC.^14,15^ In addition, we included both missense and loss of function variants, whereas prior studies only included loss of function variants.

For DCM, little is known about the prevalence of DCM-causing variants in the general population. Studies focusing on truncating *TTN* variants in the general population found a prevalence ranging between 1:33 and 1:526.^16,17^ This wide range can partly be explained by the used definition of pathogenicity. Also, disease-causing truncating *TTN* variants associated with DCM are known to be highly enriched in the A band. However, recently, truncating variants in the distal I-band region have also been implicated in DCM.^18^ When solely focussing on *TTN* variants, we found a prevalence of only 1:735. This differs from the previous studies, probably because not all *TTN* variants are reported as pathogenic or likely pathogenic in ClinVar and VKGL. Including all curated DCM-associated genes, we report a prevalence of 1:251.

For HCM, we found a prevalence ranging between 1:250 and 1:149 individuals carrying a pathogenic or likely pathogenic variant, which approaches previous estimates of 1:164.^19^ In a recent study, including the UKB population, a prevalence of 1:407 was reported.^20^ They included 8 sarcomere-encoding genes described to be associated with HCM (*ACTC1, MYBPC3*, *MYH7*, *MYL2*, *MYL3*, *TNNI3*, *TNNT2*, and *TPM1*) and variants that were described as pathogenic or likely pathogenic in ClinVar or annotated as pathogenic or likely pathogenic according to the American College of Medical Genetics and Genomics criteria and filtered variants for an allele frequency of 0.00004. We included additional genes (*CSRP3*, *JPH2*, and *TNNC1*) and pathogenic and likely pathogenic variants from the VKGL database and filtered for a minor allele frequency of 0.001. Especially the latter is a driving force behind the higher prevalence in this study. When also using a minor allele frequency (MAF) <0.00004, the prevalence of our study would be 1:475, approaching the prevalence reported by de Marvao et al.^20^

### Disease Expression of Pathogenic and Likely Pathogenic Variants in the General Population

Most information on disease penetrance in ARVC, DCM, or HCM G+ is based on observations in G+ relatives of cardiomyopathy patients. Previous studies have shown that 37% of ARVC G+ relatives^21^ and up to 50% of HCM G+ relatives with sarcomeric variants^22^ show disease expression during follow-up. Our findings suggest that disease penetrance in the general population is much lower. We found that 1.2% of ARVC G+, 3.1% of DCM G+ and 2.6% of HCM G+ in the UKB were diagnosed with a cardiomyopathy or heart failure, in the absence of chronic ischemic heart disease. Our additional analysis of ventricular function and Electrocardiography in undiagnosed G+ subjects also suggests a low disease penetrance. We found significantly worse LVEF and strain parameters in DCM G+P− compared with controls; however, none met the diagnostic Henry criteria (LVEF below 45% and LVEDVi 2 times the normal SD).^11^ Although CMR data were only available in a subgroup of undiagnosed G+ patients, these findings make it unlikely that the low penetrance found in our study arises from missed diagnoses or covert disease in the G+ cohort. Furthermore, none of the G+P− individuals met HCM criteria^12^ of ≥15 mm wall thickness, 2 individuals did meet the criteria for limited hypertrophy (13–15 mm) in the presence of a positive genetic test.^12^ Interestingly, maximum wall thickness in de Marvao et al^20^ was higher compared with ours. Although this can partly be explained by the inclusion of P+ by Marvao et al, this may also be explained by differences in wall thickness calculation method. While Marvao et al uses the absolute largest wall thickness value at a single point, we have used the American Heart Association segment with the largest wall thickness (which is an average of all the single points within 1 American Heart Association segment to reduce random outliers). In ARVC and DCM G+P− we found a low, but significantly higher prevalence of ventricular arrhythmias compared with controls (1.7% versus 0.3% (OR 5.85 [95% CI 1.98; 14.40]) and 1.0% versus 0.3% (OR 3.43 [95% CI 1.35; 7.68)) respectively). In ARVC, electrical abnormalities are known to precede structural abnormalities.^23^ Therefore, these findings may suggest early disease penetrance in a small subset of undiagnosed G+ individuals. The discrepancy between the high disease penetrance found in G+ family members and the low penetrance in the G+ general population points towards the interaction of possible other (unidentified) genetic and environmental factors leading to this variation. The median age of our study population was 57 [49–63] years; however; inherited cardiomyopathies are generally diagnosed at a younger age. For ARVC, Groeneweg et al showed, in a cohort of 439 index-patients, that the mean age of first disease presentation is 36±14 years. Most of these patients presented with symptoms (95%), of whom 11% with sudden cardiac arrest.^21^ Likewise in DCM, the mean age of presentation is mostly between 30-50 years.^24^ Lastly, in HCM a mean age at presentation of 49±16 years was shown in a cohort of 4893 patients by Lorenzini *et al.*.^25^ Interestingly, although mortality rates were low, young HCM patients showed a worse prognosis compared with their healthy peers, with 80% of mortality being caused by sudden cardiac death.^25^ Therefore, it should be taken into account that younger patients with disease expression are likely underrepresented in our study. This is not only due to higher mortality and morbidity in especially ARVC and HCM, but also because individuals with a diagnosed cardiomyopathy may be less likely to participate in a large-scale biobank study such as the UKB.

Interestingly, the South Asian *MYBPC3* and the *TNNT2* variant, showed a relatively high prevalence in our cohort. In total, 19% of HCM G+ was Asian and most of these individuals carried the c.3628-41_3628-17del variant in the *MYBPC3* gene. Although this variant is indicated as likely pathogenic in ClinVar, a previous study suggests that this variant may be reclassified as benign.^26^ In our study, none of these variant carriers were diagnosed with HCM. Four were diagnosed with heart failure of whom 1 was diagnosed with DCM. This suggests that this variant is associated with heart failure in the setting of multiple forms of cardiomyopathy, and not simply HCM.^26^ Secondly, the c.862C>T p.Arg288Cys variant in *TNNT2* was previously found in HCM individuals but is often observed in patients with a mild phenotype or in combination with other variants. These observations suggest that this variant might not be a monogenic cause of severe HCM but acts in concert with other variants.^27^ Interestingly, when excluding these variants from our G+P- population, a significantly higher wall thickness is measured compared with controls. These 2 examples emphasize that when pathogenic or likely pathogenic variants are identified as a secondary finding, other factors, such as the specific variant and the family history, are crucial for follow-up decisions.

We also assessed gene-specific associations with the cardiovascular outcomes. *PKP2* variant carriers showed a stronger association with ventricular arrhythmias (OR 11.90 [95% CI 4.38; 27.86], p = 6.4×10^–6^) compared with heart failure (OR 1.50 [95% CI 0.48; 3.64], *P=*0.395). This is in concordance with a previous study showing sustained ventricular arrhythmias to be the first clinical presentation in 61% of ARVC patients.^21^ During follow-up, sustained arrhythmias occurred in 72% of ARVC patients, highlighting sustained arrhythmias as the most important ARVC disease manifestation. On the contrary, symptomatic heart failure was seen in 13% of ARVC patients.^21^ In DCM G+, ventricular arrhythmias were significantly more present compared with G– controls, especially in *TTN* (OR 4.49 [95% CI 1.15; 12.76], *P=*0.016), *DES* (OR 12.80 [95% CI 1.45; 52.55], *P=*0.013) and *LMNA* (OR 15.04 [95% CI 1.69; 62.32], *P=*0.009) variant carriers. A recent meta-analysis assessing predictors for sustained ventricular arrhythmias, showed *PLN* and *LMNA* to be associated with arrhythmogenic outcome.^28^ Although we did not have enough power to study *PLN* G+, *LMNA* G+ did show significantly more ventricular arrhythmias compared with G− controls. Furthermore, *BAG3* variant carriers have been associated with significant risk of progressive heart failure.^29^ In our study, *BAG3* variant carriers were significantly more often diagnosed with a cardiomyopathy (OR 41.18 [95% CI 4.36; 192.17], *P=*0.002). Even though more heart failure cases were seen compared with G− controls, this did not reach statistical significance (Table S10).

Interestingly, self-reported health-related quality of life and psychological well-being of 89 asymptomatic HCM G+ were previously evaluated in a Dutch cohort and found to be at least similar to the general population, which suggests that reporting incidental findings will not harm psychological well-being of G+.^30^ However, frequent cardiological examination of G+ and family members turning out to be carriers after cascade screening will put a burden on health care and societal costs.^31^ Genetic screening and cardiological examination are necessary in family members of genetic cardiomyopathy patients since disease expression in family members is considerable. Disease expression in the general population on the other hand is low. Therefore, in case of an incidental finding, multiple factors like family history, presence of symptoms, electrical and/or structural abnormalities and gene and variant type should inform follow-up decisions. Further studies on the genotype-phenotype associations and disease penetrance will aid in facilitating these decisions.

### Limitations

Several variants are associated with more than 1 cardiomyopathy. This is mainly due to phenotypic heterogeneity but may also be partly explained by misdiagnosis. Information is submitted to ClinVar by laboratories, not by clinicians. Phenotype description might therefore be less reliable. To avoid selection bias, we included variants associated with multiple cardiomyopathies in both cardiomyopathy categories, possibly leading to increased prevalence estimates. Although the prevalence of cardiomyopathy variants is slightly affected by including or excluding overlapping variants, this did not substantially affect the results and conclusions (Table S11). Future studies should focus on reaching consensus on variant-phenotype associations for the variants described in multiple cardiomyopathies to avoid variation in prevalence caused by the use of different definitions. Despite recent efforts to harmonize knowledge on genes associated with inherited cardiomyopathies,^5–7^ and guidelines for variant classification,^31^ the adjudication of the clinical significance of single variants can still differ between diagnostic laboratories,^31^ which has led to interpretation differences and difficulties to compare results among studies using different criteria. This highlights the importance of a single set of criteria to ascertain clinical significance of a single variant. Furthermore, not all pathogenic or likely pathogenic variants are reported in these databases, especially family-specific variants and pathogenic variants in non-White populations are underreported.

Lastly, G+P− and G−P− individuals with CMR data available were age, sex, and ethnicity matched and comparable in the presence of cardiovascular risk factors and diseases. Interestingly, outliers in CMR values were also present in G−P− controls, which could be partly explained by the presence of past myocardial infarctions. Therefore, differences in cardiac function and structure between G+P+ and G−P− could be underestimated.

## Conclusions

In a cohort of 200,643 individuals with whole exome sequencing and phenotype data we identified a prevalence of pathogenic variants associated with ARVC, DCM, and HCM of 1:578, 1:251 and 1:149 respectively. Among the identified G+ individuals, cardiomyopathy, heart failure and ventricular arrhythmias were more common compared with G−. However, overall disease penetrance was low (1.2–3.1%). Therefore, in case of incidental findings, decisions on application of cascade screening and frequency of cardiological examination should be based on multiple factors besides variant and gene type, such as family history and disease expression.

## Article Information

### Acknowledgment

This research has been conducted using the UK Biobank Resource under Application Number 24711.

### Sources of Funding

The work was financially supported by the Netherlands Cardiovascular Research Initiative, an initiative supported by the Dutch Heart Foundation (CardioVasculair Onderzoek Nederland (CVON) projects: DOUBLE-DOSE 2020B005 (AB), PREDICT2 2018-30, eDETECT 2015-12 (PvT, Art and FA) and PREDICT Young Talent Program (Art)). In addition, this work was supported by the Dutch Heart Foundation (2015T058 (Art), 2015T041 (AB) and 2019T045 (MvV and JvS)). Furthermore, MB is supported by the Alexandre Suerman Stipend of the UMC Utrecht (2017), Art by the UMC Utrecht Fellowship Clinical Research Talent and FA by the UCL Hospitals NIHR Biomedical Research Center.

### Disclosures

None

### Supplemental Material

Supplemental Methods

Tables S1–S11

Figures S1–S4

References^32–38^

## Supplementary Material
