# Supplementary material for: Prevalence and Disease Expression of Pathogenic and Likely Pathogenic Variants Associated With Inherited Cardiomyopathies in the General Population
Source: Circ Genom Precis Med. 2022 Oct 20;15(6):e003704. doi: 10.1161/CIRCGEN.122.003704 (PMC9770140; doi:10.1161/CIRCGEN.122.003704)
Supplement: Supplementary file 1 [file hcg-15-e003704-s001.pdf]

Supplemental Material

Content

Supplemental Methods……………………………………………………………………………………………………..2

Supplemental Tables……………………………………………………………………………………………………….5

Supplemental Figures…………………………………………………………………………………………………….30

Supplemental Methods

Genetic variants in the study population

We identified carriers of a pathogenic or likely pathogenic variant associated with ARVC, DCM or HCM in individuals from the UKB who underwent whole exome sequencing (WES, n=200,643 at time of analysis). For each inherited cardiomyopathy we selected curated genes classified to have definite, strong or moderate evidence of pathogenicity as defined by the standardized evidence-based framework of Clinical Genome Resource (ClinGen)[32] and curated by James *et al*.[5] for ARVC, Jordan *et al.*[6] for DCM and Ingles *et al*.[7] for HCM. For ARVC we included *DES, DSC2, DSG2, DSP, JUP, PKP2, PLN* and *TMEM43;* for DCM we included *ACTC1, ACTN2, BAG3, DES, DSP, FLNC, JPH2, LMNA, MYH7, NEXN, PLN, RBM20, SCN5A, TNNC1, TNNI3, TNNT2, TPM1, TTN* and *VCL;* and for HCM we included *ACTC1, CSRP3, JPH2, MYBPC3, MYH7, MYL2, MYL3, TNNC1, TNNI3, TNNT2* and *TPM1* (**Figure 1** and **Table I**). Some genetic variants are associated with two cardiomyopathies (**Table IV**). Individuals carrying these variants were included in the G+ groups of both cardiomyopathies. Next, likely pathogenic and pathogenic variants in these genes were identified using the ClinVar NCBI-NIH database[8] and the Dutch Society for Clinical Genetic Laboratory Diagnostics (Vereniging Klinische Genetische Laboratoriumdiagnostiek, VKGL) database. Laboratories submitting information to these databases use the criteria for variant classification as defined by the American College of Medical Genetics and Genomics and the Association for Molecular Pathology (ACMG-AMP)[33]. An elaborate overview of the ClinVar and VKGL search criteria is given in **Figure 2**. In short, ClinVar was queried using the disease name(s) and filtered for pathogenic and likely pathogenic variants in the curated genes. For variants mentioned in the VKGL database, which does not specify disease associations, association with one of the cardiomyopathies was confirmed in ClinVar. The minor allele frequency

(MAF) cut-off was defined at 0.001, which is the recommended cut-off for including rare variants[34] and still include any potentially at-risk variant carriers. Variants were classified as missense or loss of function (LoF), with LoF being defined as frameshift, stop gain, start lost and canonical splice site variants.

We matched G+ individuals in a 1:4 ratio to UKB individuals without a pathogenic or likely pathogenic variant associated with one of the cardiomyopathies (G-). Matching of this G-control group was based on age, sex, ethnicity and presence of cardiac magnetic resonance imaging (CMR) measurements. Controls are referred to as G- throughout this study.

Data extraction UKB

<u>Disease definitions</u>

An elaborate overview of the disease definitions used in this study is available in **Table II**. In short, individuals were defined to be phenotype positive (P+) if they had an ICD-10 or self-reporting code for cardiomyopathy, DCM, HCM or heart failure, without a diagnosis of chronic ischemic heart disease. No ICD-10 or self-reporting code was available for ARVC in the UKB.

<u>CMR and ECG data analysis</u>

We investigated disease expression on cardiac magnetic resonance imaging (CMR, n = 225 unique individuals) and electrocardiography (ECG, n = 231 unique individuals) of G+P- individuals. The full CMR protocol of the UKB has been described in detail[35]. In short, all CMR examinations were performed on a 1.5 Tesla scanner (Magnetom Aera, Syngo Platform VD13A, Siemens Healthcare, Erlangen, Germany). We used a previously developed and

validated deep-learning methodology (AI-CMR^{QC}) to extract left (LV) and right ventricular (RV) CMR measurements[36]. In short, cine images of short-axis and 2- and 4-chamber long-axis views were used to automatically calculate LV and RV functional measures (ejection fraction [EF], stroke volume [SV]) and structural measures (end diastolic volume [EDV], end-systolic volume [ESV], LV end diastolic mass [EDM], LV mass to EDV ratio [LVMVR] and LV maximal and regional [16 segments model according the American Heart Association[37]] wall thickness).

The electrocardiography (ECG) variables P duration, P axis, PQ interval, QRS duration, R axis, QTc interval and T axis were extracted from the UKB for G+P- individuals.

Statistical analysis

Statistical analysis was performed using R version 4.0.2[38]. Continuous values are presented as median [interquartile range] and for comparisons of two groups, Mann-Whitney-U test was used. Categorical data was displayed as absolute frequency (n) and percentages (%) and Fisher's exact test was used to test for differences. The strength of the association between cardiac outcomes and G+ ARVC, DCM and HCM was calculated by the odds ratio with 95% confidence intervals. The latter was also performed stratifying by genes.

A number of included variants are associated with more than one cardiomyopathy. To investigate the effect of the inclusion of individuals in more than one cardiomyopathy, we removed overlapping variants as described in **Table IV** and calculated the odds ratios of the cardiovascular risk factors and diagnoses for these G+ compared to G-.

A p-value of less than 0.05 was considered significant.

Supplementary Table I: Included curated genes per cardiomyopathy			
**Gene***	**ARVC**	**DCM**	**HCM**
ACTC1		Moderate	Definitive
ACTN2		Moderate	
BAG3		Definitive	
CSRP3			Moderate
DES	Moderate	Definitive	
DSC2	Definitive		
DSG2	Definitive		
DSP	Definitive	Strong	
FLNC		Definitive	
JPH2		Moderate	Moderate
JUP	Definitive		
LMNA		Definitive	
MYBPC3			Definitive
MYH7		Definitive	Definitive
MYL2			Definitive
MYL3			Definitive
NEXN		Moderate	
PKP2	Definitive		
PLN	Moderate	Definitive	
RBM20		Definitive	
SCN5A		Definitive	
TMEM43	Definitive		
TNNC1		Definitive	Moderate
TNNI3		Moderate	Definitive
TNNT2		Definitive	Definitive
TPM1		Moderate	Definitive
TTN		Definitive	
VCL		Moderate	

* ARVC genes are curated by ref 5, DCM genes by ref 6 and HCM genes by ref 7. Pathogenicity is classified as moderate, strong and definitive.

Abbreviations:
ACTC1 : Actin Alpha Cardiac Muscle 1; *ACTN2* : Alpha-actinin 2;
ARVC: Arrhythmogenic right ventricular cardiomyopathy; *BAG3* : BAG Cochaperone 3;
CSRP3 : Cysteine And Glycine Rich Protein 3; DCM: Dilated cardiomyopathy; *DES* : Desmin;
DSC2 : Desmocollin 2; *DSG2* : Desmoglein 2; *DSP* : Desmoplakin; *FLNC* : Filamin-C;
HCM: Hypertrophic cardiomyopathy; *JPH2* : Junctophilin 2; *JUP* : Junction Plakoglobin;
LMNA : Lamin A/C; *MYBPC3* : Myosin Binding Protein C3; *MYH7* : Myosin Heavy Chain 7;
MYL2 : Myosin Light Chain 2; *MYL3* : Myosin Light Chain 3; *NEXN* : Nexilin F-Actin Binding Protein;
PKP2 : Plakophilin 2; *PLN* : phospholamban; *RBM20* : RNA Binding Motif Protein 20;
SCN5A : Sodium Voltage-Gated Channel Alpha Subunit 5; *TMEM43* :Transmembrane Protein 43;
TNNC1 : Troponin C1, Slow Skeletal And Cardiac Type; *TNNI3* : Troponin I3, Cardiac Type;
TNNT2 : Troponin T2, Cardiac Type; *TPM1* : Tropomyosin 1; *TTN* : Titin; *VCL* : Vinculin.

Supplementary Table II: Disease definitions		
**Phenotype**	**Field names**	**Values (ICD or other coding)**
**Diabetes**	Diagnoses ICD10<br>Underlying (primary) cause of death: ICD10<br>Contributory (secondary) causes of death: ICD10<br>External causes ICD10<br>Diagnoses main ICD10<br>Diagnoses secondary ICD10	E10*; E11*; E12*; E13*; E14*
	Non-cancer illness code self-reported	1220; 1222; 1223
	Diabetes diagnosed by doctor	1
	Medication for cholesterol blood pressure or diabetes(, or take exogenous hormones)	3
**Hypertension**	Diagnoses ICD10<br>Underlying (primary) cause of death: ICD10<br>Contributory (secondary) causes of death: ICD10<br>External causes ICD10<br>Diagnoses main ICD10<br>Diagnoses secondary ICD10	I10; I15*
	Non-cancer illness code self-reported	1065; 1072
	Medication for cholesterol blood pressure or diabetes(, or take exogenous hormones)	2
**Hypercholesterolaemia**	Diagnoses ICD10<br>Underlying (primary) cause of death: ICD10<br>Contributory (secondary) causes of death: ICD10<br>External causes ICD10<br>Diagnoses main ICD10<br>Diagnoses secondary ICD10	E780
	Non-cancer illness code self-reported	1473
	Medication for cholesterol blood pressure or diabetes(, or take exogenous hormones)	1
**Ever smoked**	Smoking status	1; 2
**Family heart disease**	Illnesses of father<br>Illnesses of mother<br>Illnesses of siblings	1
**Cardiac problem**	Non-cancer illness code self-reported	1066
**Heart failure**	Diagnoses ICD10<br>Underlying (primary) cause of death: ICD10<br>Contributory (secondary) causes of death: ICD10<br>External causes ICD10<br>Diagnoses main ICD10<br>Diagnoses secondary ICD10	I110; I130; I132; I50*
	Non-cancer illness code self-reported	1076
**Cardiomyopathy**	Diagnoses ICD10<br>Underlying (primary) cause of death: ICD10<br>Contributory (secondary) causes of death: ICD10<br>External causes ICD10<br>Diagnoses main ICD10<br>Diagnoses secondary ICD10	I42*
	Non-cancer illness code self-reported	1079
**Dilated cardiomyopathy**	Diagnoses ICD10<br>Underlying (primary) cause of death: ICD10<br>Contributory (secondary) causes of death: ICD10<br>External causes ICD10<br>Diagnoses main ICD10<br>Diagnoses secondary ICD10	I420
**Hypertrophic cardiomyopathy**	Diagnoses ICD10<br>Underlying (primary) cause of death: ICD10<br>Contributory (secondary) causes of death: ICD10<br>External causes ICD10<br>Diagnoses main ICD10<br>Diagnoses secondary ICD10	I421; I422
	Non-cancer illness code self-reported	1588
**Ventricular arrhythmias**	Diagnoses ICD10<br>Underlying (primary) cause of death: ICD10<br>Contributory (secondary) causes of death: ICD10<br>External causes ICD10<br>Diagnoses main ICD10<br>Diagnoses secondary ICD10	I470; I472; I490; I493
**Atrial arrhythmias**	Diagnoses ICD10<br>Underlying (primary) cause of death: ICD10<br>Contributory (secondary) causes of death: ICD10<br>External causes ICD10<br>Diagnoses main ICD10<br>Diagnoses secondary ICD10	I48*; I471; I491
	Non-cancer illness code self-reported	1471; 1483; 1487
**Heart arrhythmia**	Non-cancer illness code self-reported	1077
**Chronic ischemic heart disease**	Diagnoses ICD10<br>Underlying (primary) cause of death: ICD10<br>Contributory (secondary) causes of death: ICD10<br>External causes ICD10<br>Diagnoses main ICD10<br>Diagnoses secondary ICD10	I25*

Phenotype	Field names	Values (ICD or other coding)
**Acute myocardial infarction**	Diagnoses ICD10<br>Underlying (primary) cause of death: ICD10<br>Contributory (secondary) causes of death: ICD10<br>External causes ICD10<br>Diagnoses main ICD10<br>Diagnoses secondary ICD10	I21*; I22*; I248; I249
	Non-cancer illness code self-reported	1075
**Cardiac arrest**	Diagnoses ICD10<br>Underlying (primary) cause of death: ICD10<br>Contributory (secondary) causes of death: ICD10<br>External causes ICD10<br>Diagnoses main ICD10<br>Diagnoses secondary ICD10	I46*
**Angina pectoris**	Non-cancer illness code self-reported	1074
**Conduction disorders**	Diagnoses ICD10<br>Underlying (primary) cause of death: ICD10<br>Contributory (secondary) causes of death: ICD10<br>External causes ICD10<br>Diagnoses main ICD10<br>Diagnoses secondary ICD10	I44*; I45*
**Valvular disease**	Diagnoses ICD10<br>Underlying (primary) cause of death: ICD10<br>Contributory (secondary) causes of death: ICD10<br>External causes ICD10<br>Diagnoses main ICD10<br>Diagnoses secondary ICD10	I34*; I35*; I36*; I37*; I05*; I06*; I07*; I08*
	Non-cancer illness code self-reported	1078; 1488; 1489; 1490; 1584; 1585; 1586; 1587
**Congenital heart disease**	Diagnoses ICD10<br>Underlying (primary) cause of death: ICD10<br>Contributory (secondary) causes of death: ICD10<br>External causes ICD10<br>Diagnoses main ICD10<br>Diagnoses secondary ICD10	Q20*; Q21*; Q22*; Q23*; Q24*; Q25*; Q26*
**Pulmonary obstructive disease**	Diagnoses ICD10<br>Underlying (primary) cause of death: ICD10<br>Contributory (secondary) causes of death: ICD10<br>External causes ICD10<br>Diagnoses main ICD10<br>Diagnoses secondary ICD10	J44*; J43*; I26*; I27*
	Non-cancer illness code self-reported	1112; 1113; 1114; 1115; 1121
**All-cause mortality**	Date of Death	Any non-missing value
**Cardiovascular death**	Underlying (primary) cause of death: ICD10<br>Contributory (secondary) causes of death: ICD10	I*

* indicates starting with previously indicated code.

Supplementary Table III: Extensive baseline table

	Controls G-				ARVC G+			
	**Overall**	**Diagnosed**	**Non-Diagnosed**	**Missing**	**Overall**	**Diagnosed**	**Non-Diagnosed**	**Missing**
n (%)	9,972 (100)	87 (0.8)	9,885 (99.2)		347 (100)	4 (1.2)	343 (98.8)	
**Sex = Female (%)**	5,436 (54.5)	35 (40.2)	5,401 (54.6)	0	187 (53.9)	2 (50.0)	185 (53.9)	0
**Age (median [IQR])**	57.00 [49.00, 63.00]	62.00 [56.00, 66.00]	57.00 [49.00, 63.00]	0	57.00 [50.00, 64.00]	55.50 [52.25, 58.50]	57.00 [50.00, 64.00]	0
**Ethnicity (%)**				1				0.6
Asian	1,076 (10.9)	5 (5.8)	1071 (10.9)		10 (2.9)	0 (0.0)	10 (2.9)	
Black	164 (1.7)	3 (3.5)	161 (1.6)		7 (2.0)	1 (25.0)	6 (1.8)	
Chinese	56 (0.6)	1 (1.2)	55 (0.6)		11 (3.2)	0 (0.0)	11 (3.2)	
Mixed	132 (1.3)	2 (2.3)	130 (1.3)		1 (0.3)	0 (0.0)	1 (0.3)	
Other	160 (1.6)	1 (1.2)	159 (1.6)		5 (1.4)	0 (0.0)	5 (1.5)	
White	8,288 (83.9)	74 (86.0)	8,214 (83.9)		311 (90.1)	3 (75.0)	308 (90.3)	
**CARDIOVASCULAR RISK FACTORS**								
**BMI (median [IQR])**	26.73 [24.15, 29.82]	28.83 [25.33, 32.20]	26.71 [24.15, 29.80]	0.5	26.40 [24.03, 30.32]	29.29 [27.22, 32.45]	26.36 [24.02, 30.18]	0.3
**Diabetes (%)**	914 (9.2)	21 (24.1)	893 (9.0)	0	35 (10.1)	1 (25.0)	34 (9.9)	0
**Hypertension (%)**	3,420 (34.3)	60 (69.0)	3,360 (34.0)	0	116 (33.4)	3 (75.0)	113 (32.9)	0
**Mean systolic blood pressure (median [IQR])**	135.50 [124.00, 149.00]	144.00 [130.00, 155.50]	135.50 [124.00, 149.00]	0.1	136.00 [124.00, 147.50]	128.25 [120.62, 134.38]	136.25 [124.00, 147.50]	0.3
**Mean diastolic blood pressure (median [IQR])**	82.00 [75.00, 88.50]	84.00 [76.50, 91.50]	81.50 [75.00, 88.50]	0.1	81.50 [75.00, 87.88]	82.00 [77.38, 85.25]	81.50 [75.00, 88.00]	0.3
**Hypercholesterolaemia (%)**	2,416 (24.2)	34 (39.1)	2,382 (24.1)	0	86 (24.8)	3 (75.0)	83 (24.2)	0
**Total cholesterol (median [IQR])**	5.61 [4.86, 6.38]	5.23 [4.39, 6.10]	5.61 [4.87, 6.38]	4.3	5.51 [4.82, 6.40]	4.97 [4.50, 5.78]	5.51 [4.83, 6.40]	4.6
**HDL (median [IQR])**	1.38 [1.16, 1.65]	1.29 [1.13, 1.56]	1.38 [1.16, 1.65]	11.3	1.38 [1.16, 1.64]	1.48 [1.30, 1.65]	1.38 [1.16, 1.64]	9.2
**LDL (median [IQR])**	3.50 [2.92, 4.09]	3.22 [2.54, 3.81]	3.50 [2.92, 4.09]	4.6	3.39 [2.87, 4.04]	3.06 [2.71, 3.58]	3.40 [2.88, 4.04]	4.6
**Ever Smoked (%)**	4,132 (41.4)	50 (57.5)	4,082 (41.3)	0	161 (46.4)	2 (50.0)	159 (46.4)	0
**MET minutes per week for walking (median [IQR])**	693.00 [297.00, 1,386.00]	528.00 [255.75, 1,608.75]	693.00 [297.00, 1,386.00]	19.8	693.00 [297.00, 1,386.00]	569.25 [247.50, 952.88]	693.00 [297.00, 1,386.00]	19.3
**MET minutes per week for moderate activity (median [IQR])**	480.00 [120.00, 1,200.00]	480.00 [100.00, 1,680.00]	480.00 [120.00, 1,200.00]	19.8	480.00 [120.00, 1,200.00]	900.00 [640.00, 1,140.00]	480.00 [120.00, 1,200.00]	19.3
**MET minutes per week for vigorous activity (median [IQR])**	240.00 [0.00, 960.00]	0.00 [0.00, 820.00]	240.00 [0.00, 960.00]	19.8	320.00 [0.00, 960.00]	1,060.00 [540.00, 1,410.00]	320.00 [0.00, 960.00]	19.3
**Total MET minutes per week (median [IQR])**	1,773.00 [810.00, 3,452.50]	1,367.50 [478.50, 3,834.00]	1,776.50 [810.00, 3,450.00]	19.8	2,001.00 [922.50, 3,550.50]	2,529.25 [1,517.50, 3,412.88	1,969.00 [922.50, 3,550.50]	19.3
**Family heart disease (%)**	4,458 (44.7)	34 (39.1)	4,424 (44.8)	0	179 (51.6)	4 (100.0)	175 (51.0)	0
**CARDIAC DISEASES/OUTCOMES**								
**Cardiac problem (%)**	41 (0.4)	2 (2.3)	39 (0.4)	0	3 (0.9)	0 (0.0)	3 (0.9)	0
**Heart failure (%)**	182 (1.8)	74 (85.1)	108 (1.1)	0	9 (2.6)	2 (50.0)	7 (2.0)	0
**Cardiomyopathy (%)**	37 (0.4)	26 (29.9)	11 (0.1)	0	3 (0.9)	3 (75.0)	0 (0.0)	0
**Dilated cardiomyopathy (%)**	14 (0.1)	8 (9.2)	6 (0.1)	0	2 (0.6)	2 (50.0)	0 (0.0)	0
**Hypertrophic cardiomyopathy (%)**	8 (0.1)	7 (8.0)	1 (0.0)	0	1 (0.3)	1 (25.0)	0 (0.0)	0
**Ventricular arrhythmias (%)**	33 (0.3)	3 (3.4)	30 (0.3)	0	7 (2.0)	1 (25.0)	6 (1.7)	0
**Atrial arrhythmias (%)**	191 (1.9)	19 (21.8)	172 (1.7)	0	7 (2.0)	0 (0.0)	7 (2.0)	0
**Heart arrhythmia (%)**	54 (0.5)	2 (2.3)	52 (0.5)	0	6 (1.7)	1 (25.0)	5 (1.5)	0
**Chronic ischemic heart disease (%)**	725 (7.3)	0 (0.0)	725 (7.3)	0	35 (10.1)	0 (0.0)	35 (10.2)	0
**Acute myocardial infarction (%)**	298 (3.0)	1 (1.1)	297 (3.0)	0	15 (4.3)	0 (0.0)	15 (4.4)	0
**Cardiac arrest (%)**	34 (0.3)	1 (1.1)	33 (0.3)	0	0 (0.0)	0 (0.0)	0 (0.0)	0
**Angina pectoris (%)**	312 (3.1)	2 (2.3)	310 (3.1)	0	16 (4.6)	0 (0.0)	16 (4.7)	0
**Conduction disorders (%)**	151 (1.5)	10 (11.5)	141 (1.4)	0	8 (2.3)	0 (0.0)	8 (2.3)	0
**Valvular disease (%)**	241 (2.4)	23 (26.4)	218 (2.2)	0	11 (3.2)	1 (25.0)	10 (2.9)	0
**Congenital heart disease (%)**	28 (0.3)	3 (3.4)	25 (0.3)	0	2 (0.6)	0 (0.0)	2 (0.6)	0
**Pulmonary obstructive disease (%)**	494 (5.0)	24 (27.6)	470 (4.8)	0	25 (7.2)	0 (0.0)	25 (7.3)	0
**Cardiovascular death (%)**	181 (1.8)	13 (14.9)	168 (1.7)	0	11 (3.2)	0 (0.0)	11 (3.2)	0
**All-cause mortality (%)**	513 (5.1)	27 (31.0)	486 (4.9)	0	19 (5.5)	0 (0.0)	19 (5.5)	0
**ECG MEASUREMENTS**								
**n (%)**	1,062 (10.6)	4 (4.6)	1,058 (10.7)		32 (9.2)	0 (0.0)	32 (9.3)	
**P duration (median [IQR])**	100.00 [90.00, 108.00]	90.00 [82.00, 111.00]	100.00 [90.00, 108.00]	89.8	100.00 [93.50, 111.50]	NA	100.00 [93.50, 111.50]	90.8
**P axis (median [IQR])**	55.00 [40.25, 67.00]	47.00 [47.00, 47.00]	55.00 [40.00, 67.00]	93.1	54.00 [42.25, 61.50]	NA	54.00 [42.25, 61.50]	94.8
**PQ interval (median [IQR])**	160.00 [145.50, 178.00]	188.00 [188.00, 188.00]	160.00 [145.00, 178.00]	93.1	171.00 [147.00, 183.00]	NA	171.00 [147.00, 183.00]	94.8
**QRS duration (median [IQR])**	86.00 [80.00, 94.00]	93.00 [89.50, 97.00]	86.00 [80.00, 94.00]	89.4	88.00 [81.50, 96.00]	NA	88.00 [81.50, 96.00]	90.8
**R axis (median [IQR])**	34.00 [7.00, 58.00]	-48.00 [-48.00, -48.00]	34.00 [7.50, 58.00]	92.9	23.50 [-1.75, 50.00]	NA	23.50 [-1.75, 50.00]	94.8
**QTC interval (median [IQR])**	417.00 [402.00, 433.00]	511.00 [511.00, 511.00]	417.00 [402.00, 432.50]	92.9	429.50 [403.25, 440.00]	NA	429.50 [403.25, 440.00]	94.8
**T axis (median [IQR])**	40.00 [23.00, 55.25]	92.00 [92.00, 92.00]	40.00 [23.00, 55.00]	92.9	35.50 [20.25, 54.25]	NA	35.50 [20.25, 54.25]	94.8
**CMR MEASUREMENTS**								
**n (%)**	990 (9.9)	4 (4.6)	986 (10.0)		33 (9.5)	0 (0.0)	33 (9.6)	
**RVEDVi (median [IQR])**	80.18 [70.62, 90.27]	79.28 [79.28, 79.28]	80.19 [70.61, 90.27]	91	79.14 [73.73, 92.49]	NA	79.14 [73.73, 92.49]	91.1
**RVESVi (median [IQR])**	32.89 [27.38, 39.68]	20.93 [20.93, 20.93]	32.90 [27.42, 39.70]	91	35.16 [29.98, 38.70]	NA	35.16 [29.98, 38.70]	91.1
**RVSVi (median [IQR])**	46.55 [40.92, 52.84]	58.35 [58.35, 58.35]	46.53 [40.91, 52.81]	91	48.22 [41.97, 52.24]	NA	48.22 [41.97, 52.24]	91.1
**RVEF (median [IQR])**	58.38 [54.19, 62.76]	73.60 [73.60, 73.60]	58.37 [54.19, 62.74]	91	58.30 [53.30, 62.06]	NA	58.30 [53.30, 62.06]	91.1
**RVPER (median [IQR])**	388.72 [316.56, 465.82]	446.55 [446.55, 446.55]	388.66 [316.54, 465.97]	91	405.50 [291.73, 489.37]	NA	405.50 [291.73, 489.37]	91.1
**RVPFR (median [IQR])**	300.61 [245.17, 364.00]	373.24 [373.24, 373.24]	300.34 [245.12, 363.44]	91	302.85 [225.65, 375.82]	NA	302.85 [225.65, 375.82]	91.1
**RVPAFR (median [IQR])**	282.95 [222.68, 360.32]	547.72 [547.72, 547.72]	282.86 [222.56, 360.07]	91	274.74 [213.71, 343.90]	NA	274.74 [213.71, 343.90]	91.1
**LVEDVi (median [IQR])**	74.33 [66.34, 83.11]	64.68 [59.39, 69.33]	74.37 [66.38, 83.15]	91.9	80.77 [73.11, 88.68]	NA	80.77 [73.11, 88.68]	91.6
**LVESVi (median [IQR])**	30.02 [25.12, 35.70]	26.43 [25.24, 28.39]	30.02 [25.13, 35.72]	91.9	31.74 [25.91, 39.55]	NA	31.74 [25.91, 39.55]	91.6
**LVSVi (median [IQR])**	44.03 [39.34, 50.28]	38.25 [31.00, 44.09]	44.05 [39.37, 50.30]	91.9	46.82 [43.25, 50.82]	NA	46.82 [43.25, 50.82]	91.6
**LVEF (median [IQR])**	59.47 [55.29, 63.52]	59.14 [51.51, 63.31]	59.48 [55.29, 63.52]	91.9	59.69 [56.59, 66.23]	NA	59.69 [56.59, 66.23]	91.6
**LVPER (median [IQR])**	373.80 [302.29, 452.71]	284.40 [256.83, 364.56]	373.81 [302.44, 453.27]	91.9	407.32 [307.20, 455.45]	NA	407.32 [307.20, 455.45]	91.6
**LVPFR (median [IQR])**	321.31 [259.23, 385.16]	201.74 [189.85, 229.92]	321.49 [259.95, 385.63]	91.9	346.24 [290.79, 422.04]	NA	346.24 [290.79, 422.04]	91.6
**LVPAFR (median [IQR])**	233.66 [167.35, 306.30]	363.87 [208.97, 466.86]	233.50 [167.46, 305.08]	91.9	208.66 [158.63, 298.40]	NA	208.66 [158.63, 298.40]	91.6
**LVEDMi (median [IQR])**	41.88 [36.52, 48.62]	45.76 [34.02, 48.75]	41.85 [36.55, 48.61]	91.9	42.81 [36.04, 48.38]	NA	42.81 [36.04, 48.38]	91.6
**LVMVR (median [IQR])**	0.56 [0.50, 0.62]	0.70 [0.56, 0.70]	0.56 [0.50, 0.62]	91.9	0.55 [0.49, 0.60]	NA	0.55 [0.49, 0.60]	91.6
**LVEDV/RVEDV (median [IQR])**	0.93 [0.86, 1.03]	0.93 [0.93, 0.93]	0.93 [0.86, 1.03]	92.2	0.94 [0.90, 1.05]	NA	0.94 [0.90, 1.05]	91.6
**LVESV/RVESV (median [IQR])**	0.91 [0.80, 1.04]	1.15 [1.15, 1.15]	0.91 [0.80, 1.04]	92.2	0.91 [0.82, 1.00]	NA	0.91 [0.82, 1.00]	91.6
**peakEcc (median [IQR])**	-22.70 [-24.98, -20.43]	-21.60 [-21.61, -21.59]	-22.72 [-24.98, -20.42]	93.7	-22.87 [-26.90, -21.63]	NA	-22.87 [-26.90, -21.63]	94.2
**TPKEcc (median [IQR])**	331.31 [309.83, 354.66]	325.97 [321.27, 330.67]	331.31 [309.75, 354.68]	93.7	326.90 [318.45, 363.83]	NA	326.90 [318.45, 363.83]	94.2
**peakEll2Ch (median [IQR])**	-21.19 [-23.34, -18.93]	-23.89 [-23.89, -23.89]	-21.17 [-23.32, -18.93]	93.8	-21.37 [-23.84, -19.31]	NA	-21.37 [-23.84, -19.31]	93.9
**TPKEll2Ch (median [IQR])**	349.86 [321.84, 379.10]	388.50 [388.50, 388.50]	349.86 [321.81, 379.08]	93.9	346.80 [321.44, 370.50]	NA	346.80 [321.44, 370.50]	93.9
**peakEll4Ch (median [IQR])**	-23.30 [-25.97, -21.37]	-21.40 [-21.40, -21.40]	-23.30 [-25.99, -21.37]	93.9	-24.25 [-26.79, -21.39]	NA	-24.25 [-26.79, -21.39]	93.7
**TPKEll4Ch (median [IQR])**	357.53 [327.07, 397.64]	360.78 [360.78, 360.78]	357.30 [326.96, 397.81]	93.9	354.30 [328.00, 406.56]	NA	354.30 [328.00, 406.56]	93.9
**Wall thickness segment 1 (median [IQR])**	7.65 [6.81, 8.50]	7.04 [5.94, 8.14]	7.65 [6.81, 8.49]	93.2	7.05 [6.26, 8.57]	NA	7.05 [6.26, 8.57]	94.2
**Wall thickness segment 2 (median [IQR])**	6.75 [5.75, 7.91]	7.50 [6.70, 8.31]	6.75 [5.74, 7.90]	93.2	6.81 [5.24, 7.75]	NA	6.81 [5.24, 7.75]	94.2
**Wall thickness segment 3 (median [IQR])**	6.05 [5.17, 6.96]	7.60 [6.78, 8.42]	6.05 [5.17, 6.95]	93.2	5.58 [4.74, 7.16]	NA	5.58 [4.74, 7.16]	94.2
**Wall thickness segment 4 (median [IQR])**	6.54 [5.82, 7.22]	8.31 [7.66, 8.97]	6.54 [5.81, 7.21]	93.2	6.06 [5.49, 6.76]	NA	6.06 [5.49, 6.76]	94.2
**Wall thickness segment 5 (median [IQR])**	6.20 [5.62, 6.96]	6.33 [5.27, 7.39]	6.20 [5.62, 6.96]	93.2	5.96 [5.48, 6.40]	NA	5.96 [5.48, 6.40]	94.2
**Wall thickness segment 6 (median [IQR])**	6.55 [5.97, 7.31]	6.61 [5.42, 7.79]	6.55 [5.98, 7.31]	93.2	6.45 [6.15, 6.79]	NA	6.45 [6.15, 6.79]	94.2
**Wall thickness segment 7 (median [IQR])**	5.73 [5.28, 6.31]	5.48 [4.90, 6.07]	5.73 [5.29, 6.31]	93.2	5.52 [5.21, 6.06]	NA	5.52 [5.21, 6.06]	94.2
**Wall thickness segment 8 (median [IQR])**	7.01 [6.28, 7.76]	8.52 [7.77, 9.27]	7.01 [6.28, 7.75]	93.2	6.97 [6.44, 7.28]	NA	6.97 [6.44, 7.28]	94.2
**Wall thickness segment 9 (median [IQR])**	7.38 [6.48, 8.25]	9.47 [8.05, 10.89]	7.38 [6.48, 8.25]	93.2	6.96 [6.18, 7.46]	NA	6.96 [6.18, 7.46]	94.2
**Wall thickness segment 10 (median [IQR])**	6.23 [5.59, 6.96]	7.07 [6.08, 8.06]	6.23 [5.60, 6.96]	93.2	5.88 [5.33, 6.23]	NA	5.88 [5.33, 6.23]	94.2
**Wall thickness segment 11 (median [IQR])**	5.62 [5.09, 6.32]	5.57 [5.19, 5.95]	5.62 [5.10, 6.32]	93.2	5.42 [4.96, 5.81]	NA	5.42 [4.96, 5.81]	94.2
**Wall thickness segment 12 (median [IQR])**	5.60 [5.22, 6.25]	5.56 [5.53, 5.60]	5.60 [5.22, 6.26]	93.2	5.38 [5.17, 6.12]	NA	5.38 [5.17, 6.12]	94.2
**Wall thickness segment 13 (median [IQR])**	5.48 [5.10, 5.91]	8.01 [7.03, 9.00]	5.48 [5.10, 5.91]	93.2	5.38 [5.24, 5.80]	NA	5.38 [5.24, 5.80]	94.2
**Wall thickness segment 14 (median [IQR])**	6.00 [5.36, 6.66]	8.33 [6.87, 9.79]	6.00 [5.36, 6.66]	93.2	5.92 [5.41, 6.60]	NA	5.92 [5.41, 6.60]	94.2
**Wall thickness segment 15 (median [IQR])**	5.09 [4.48, 5.69]	7.21 [5.80, 8.63]	5.09 [4.48, 5.69]	93.2	5.12 [4.78, 5.37]	NA	5.12 [4.78, 5.37]	94.2
**Wall thickness segment 16 (median [IQR])**	5.26 [4.80, 5.72]	8.31 [6.87, 9.75]	5.26 [4.80, 5.72]	93.2	5.32 [4.86, 5.67]	NA	5.32 [4.86, 5.67]	94.2
**Global wall thickness (median [IQR])**	6.30 [5.73, 6.84]	7.33 [7.28, 7.38]	6.30 [5.72, 6.84]	93.2	6.07 [5.57, 6.29]	NA	6.07 [5.57, 6.29]	94.2
**Septal wall thickness (median [IQR])**	6.68 [5.88, 7.35]	8.28 [7.88, 8.69]	6.67 [5.88, 7.34]	93.2	6.43 [5.69, 7.01]	NA	6.43 [5.69, 7.01]	94.2
**Maximum wall thickness (median [IQR])**	8.10 [7.24, 9.02]	10.96 [10.29, 11.64]	8.09 [7.24, 9.01]	93.2	7.81 [6.97, 8.59]	NA	7.81 [6.97, 8.59]	94.2

Abbreviations:
ARVC: arrythmogenic right ventricular cardiomyopathy; BMI: body mass index; CMR: cardiac magnetic resonance imaging; DCM: dilated cardiomyopathy; ECG: Electrocardiography; EDVi: indexed end-diastolic volume; EDMi: indexed end-diastolic mass; EF: ejection fraction; ESVi: indexed end-systolic volume; G+: carriers of likely pathogenic and pathogenic variants associated with one of the cardiomyopathies; HCM: hypertrophic cardiomyopathy; HDL: high-density lipoprotein; IQR: interquartile range; LDL: low-density lipoprotein; LV: left ventricular; MET: metabolic equivalent of task; MVR: mass to volume ratio; PAFR: peak atrial filling rate; peakEcc: peak circumferential strain; peakEll2Ch: longitudinal strain analyzed in 2-chamber view; peakEll4Ch: longitudinal strain analyzed in 4-chamber view; PER: peak ejection rate; PFR: peak filling rate; RV: right ventricular; SVi: indexed stroke volume; TPKEcc: global time to peak circumferential strain; TPKEll2Ch: global time to longitudinal strain analyzed in 2-chamber view; TPKEll4Ch: global time to longitudinal strain analyzed in 4-chamber view.

Supplementary Table III: Extensive baseline table

	Controls G-				DCM G+			
	**Overall**	**Diagnosed**	**Non-Diagnosed**	**Missing**	**Overall**	**Diagnosed**	**Non-Diagnosed**	**Missing**
n (%)	9,972 (100)	87 (0.8)	9,885 (99.2)		800 (100)	25 (3.1)	775 (96.9)	
**Sex = Female (%)**	5,436 (54.5)	35 (40.2)	5,401 (54.6)	0	450 (56.2)	15 (60.0)	435 (56.1)	0
**Age (median [IQR])**	57.00 [49.00, 63.00]	62.00 [56.00, 66.00]	57.00 [49.00, 63.00]	0	58.00 [50.75, 64.00]	62.00 [53.00, 66.00]	57.00 [50.00, 63.00]	0
**Ethnicity (%)**				1				0.6
Asian	1,076 (10.9)	5 (5.8)	1071 (10.9)		8 (1.0)	1 (4.0)	7 (0.9)	
Black	164 (1.7)	3 (3.5)	161 (1.6)		12 (1.5)	1 (4.0)	11 (1.4)	
Chinese	56 (0.6)	1 (1.2)	55 (0.6)		2 (0.3)	0 (0.0)	2 (0.3)	
Mixed	132 (1.3)	2 (2.3)	130 (1.3)		4 (0.5)	0 (0.0)	4 (0.5)	
Other	160 (1.6)	1 (1.2)	159 (1.6)		9 (1.1)	0 (0.0)	9 (1.2)	
White	8,288 (83.9)	74 (86.0)	8,214 (83.9)		760 (95.6)	23 (92.0)	737 (95.7)	
CARDIOVASCULAR RISK FACTORS								
**BMI (median [IQR])**	26.73 [24.15, 29.82]	28.83 [25.33, 32.20]	26.71 [24.15, 29.80]	0.5	26.92 [24.06, 29.90]	27.69 [24.21, 30.90]	26.92 [24.06, 29.88]	0.4
**Diabetes (%)**	914 (9.2)	21 (24.1)	893 (9.0)	0	62 (7.8)	1 (4.0)	61 (7.9)	0
**Hypertension (%)**	3,420 (34.3)	60 (69.0)	3,360 (34.0)	0	287 (35.9)	18 (72.0)	269 (34.7)	0
**Mean systolic blood pressure (median [IQR])**	135.50 [124.00, 149.00]	144.00 [130.00, 155.50]	135.50 [124.00, 149.00]	0.1	136.00 [124.00, 149.00]	136.50 [129.50, 149.50]	135.75 [124.00, 149.00]	0.1
**Mean diastolic blood pressure (median [IQR])**	82.00 [75.00, 88.50]	84.00 [76.50, 91.50]	81.50 [75.00, 88.50]	0.1	81.50 [74.50, 89.00]	80.50 [77.00, 88.50]	81.50 [74.50, 89.00]	0.1
**Hypercholesterolaemia (%)**	2,416 (24.2)	34 (39.1)	2,382 (24.1)	0	211 (26.4)	13 (52.0)	198 (25.5)	0
**Total cholesterol (median [IQR])**	5.61 [4.86, 6.38]	5.23 [4.39, 6.10]	5.61 [4.87, 6.38]	4.3	5.61 [4.90, 6.33]	5.20 [4.79, 6.24]	5.62 [4.93, 6.34]	4.2
**HDL (median [IQR])**	1.38 [1.16, 1.65]	1.29 [1.13, 1.56]	1.38 [1.16, 1.65]	11.3	1.40 [1.17, 1.65]	1.47 [1.29, 1.69]	1.40 [1.17, 1.65]	11.9
**LDL (median [IQR])**	3.50 [2.92, 4.09]	3.22 [2.54, 3.81]	3.50 [2.92, 4.09]	4.6	3.46 [2.93, 4.09]	3.23 [2.65, 4.02]	3.47 [2.94, 4.09]	4.2
**Ever Smoked (%)**	4,132 (41.4)	50 (57.5)	4,082 (41.3)	0	371 (46.4)	11 (44.0)	360 (46.5)	0
**MET minutes per week for walking (median [IQR])**	693.00 [297.00, 1,386.00]	528.00 [255.75, 1,608.75]	693.00 [297.00, 1,386.00]	19.8	577.50 [255.75, 1,386.00]	478.50 [206.25, 767.25]	577.50 [264.00, 1,386.00]	21.1
**MET minutes per week for moderate activity (median [IQR])**	480.00 [120.00, 1,200.00]	480.00 [100.00, 1,680.00]	480.00 [120.00, 1,200.00]	19.8	480.00 [120.00, 1,200.00]	400.00 [50.00, 795.00]	480.00 [120.00, 1,200.00]	21.1
**MET minutes per week for vigorous activity (median [IQR])**	240.00 [0.00, 960.00]	0.00 [0.00, 820.00]	240.00 [0.00, 960.00]	19.8	240.00 [0.00, 960.00]	40.00 [0.00, 540.00]	240.00 [0.00, 960.00]	21.1
**Total MET minutes per week (median [IQR])**	1,773.00 [810.00, 3,452.50]	1,367.50 [478.50, 3,834.00]	1,776.50 [810.00, 3,450.00]	19.8	1,695.00 [783.75, 3,536.00]	1,050.50 [681.75, 2,846.38]	1,706.00 [795.00, 3,539.00]	21.1
**Family heart disease (%)**	4,458 (44.7)	34 (39.1)	4,424 (44.8)	0	380 (47.5)	9 (36.0)	371 (47.9)	0
CARDIAC DISEASES/OUTCOMES								
**Cardiac problem (%)**	41 (0.4)	2 (2.3)	39 (0.4)	0	3 (0.4)	0 (0.0)	3 (0.4)	0
**Heart failure (%)**	182 (1.8)	74 (85.1)	108 (1.1)	0	36 (4.5)	16 (64.0)	20 (2.6)	0
**Cardiomyopathy (%)**	37 (0.4)	26 (29.9)	11 (0.1)	0	22 (2.8)	16 (64.0)	6 (0.8)	0
**Dilated cardiomyopathy (%)**	14 (0.1)	8 (9.2)	6 (0.1)	0	9 (1.1)	7 (28.0)	2 (0.3)	0
**Hypertrophic cardiomyopathy (%)**	8 (0.1)	7 (8.0)	1 (0.0)	0	7 (0.9)	6 (24.0)	1 (0.1)	0
**Ventricular arrhythmias (%)**	33 (0.3)	3 (3.4)	30 (0.3)	0	13 (1.6)	5 (20.0)	8 (1.0)	0
**Atrial arrhythmias (%)**	191 (1.9)	19 (21.8)	172 (1.7)	0	34 (4.2)	6 (24.0)	28 (3.6)	0
**Heart arrhythmia (%)**	54 (0.5)	2 (2.3)	52 (0.5)	0	12 (1.5)	2 (8.0)	10 (1.3)	0
**Chronic ischemic heart disease (%)**	725 (7.3)	0 (0.0)	725 (7.3)	0	73 (9.1)	0 (0.0)	73 (9.4)	0
**Acute myocardial infarction (%)**	298 (3.0)	1 (1.1)	297 (3.0)	0	27 (3.4)	1 (4.0)	26 (3.4)	0
**Cardiac arrest (%)**	34 (0.3)	1 (1.1)	33 (0.3)	0	6 (0.8)	1 (4.0)	5 (0.6)	0
**Angina pectoris (%)**	312 (3.1)	2 (2.3)	310 (3.1)	0	30 (3.8)	0 (0.0)	30 (3.9)	0
**Conduction disorders (%)**	151 (1.5)	10 (11.5)	141 (1.4)	0	18 (2.2)	3 (12.0)	15 (1.9)	0
**Valvular disease (%)**	241 (2.4)	23 (26.4)	218 (2.2)	0	37 (4.6)	9 (36.0)	28 (3.6)	0
**Congenital heart disease (%)**	28 (0.3)	3 (3.4)	25 (0.3)	0	3 (0.4)	1 (4.0)	2 (0.3)	0
**Pulmonary obstructive disease (%)**	494 (5.0)	24 (27.6)	470 (4.8)	0	47 (5.9)	7 (28.0)	40 (5.2)	0
**Cardiovascular death (%)**	181 (1.8)	13 (14.9)	168 (1.7)	0	24 (3.0)	5 (20.0)	19 (2.5)	0
**All-cause mortality (%)**	513 (5.1)	27 (31.0)	486 (4.9)	0	56 (7.0)	8 (32.0)	48 (6.2)	0
ECG MEASUREMENTS								
**n (%)**	1,062 (10.6)	4 (4.6)	1,058 (10.7)		87 (10.9)	0 (0.0)	87 (11.2)	
**P duration (median [IQR])**	100.00 [90.00, 108.00]	90.00 [82.00, 111.00]	100.00 [90.00, 108.00]	89.8	99.00 [86.00, 106.00]	NA	99.00 [86.00, 106.00]	90
**P axis (median [IQR])**	55.00 [40.25, 67.00]	47.00 [47.00, 47.00]	55.00 [40.00, 67.00]	93.1	49.00 [36.50, 61.00]	NA	49.00 [36.50, 61.00]	92.6
**PQ interval (median [IQR])**	160.00 [145.50, 178.00]	188.00 [188.00, 188.00]	160.00 [145.00, 178.00]	93.1	164.00 [145.00, 176.00]	NA	164.00 [145.00, 176.00]	92.6
**QRS duration (median [IQR])**	86.00 [80.00, 94.00]	93.00 [89.50, 97.00]	86.00 [80.00, 94.00]	89.4	84.00 [78.00, 92.00]	NA	84.00 [78.00, 92.00]	89.1
**R axis (median [IQR])**	34.00 [7.00, 58.00]	-48.00 [-48.00, -48.00]	34.00 [7.50, 58.00]	92.9	26.00 [-3.50, 50.00]	NA	26.00 [-3.50, 50.00]	92.1
**QTC interval (median [IQR])**	417.00 [402.00, 433.00]	511.00 [511.00, 511.00]	417.00 [402.00, 432.50]	92.9	420.00 [404.00, 435.00]	NA	420.00 [404.00, 435.00]	92.1
**T axis (median [IQR])**	40.00 [23.00, 55.25]	92.00 [92.00, 92.00]	40.00 [23.00, 55.00]	92.9	42.00 [25.50, 57.00]	NA	42.00 [25.50, 57.00]	92.1
CMR MEASUREMENTS								
**n (%)**	990 (9.9)	4 (4.6)	986 (10.0)		87 (10.9)	0 (0.0)	87 (11.2)	
**RVEDVi (median [IQR])**	80.18 [70.62, 90.27]	79.28 [79.28, 79.28]	80.19 [70.61, 90.27]	91	76.54 [69.50, 84.81]	NA	76.54 [69.50, 84.81]	90.2
**RVESVi (median [IQR])**	32.89 [27.38, 39.68]	20.93 [20.93, 20.93]	32.90 [27.42, 39.70]	91	32.21 [27.10, 37.43]	NA	32.21 [27.10, 37.43]	90.2
**RVSVi (median [IQR])**	46.55 [40.92, 52.84]	58.35 [58.35, 58.35]	46.53 [40.91, 52.81]	91	44.50 [40.74, 51.29]	NA	44.50 [40.74, 51.29]	90.2
**RVEF (median [IQR])**	58.38 [54.19, 62.76]	73.60 [73.60, 73.60]	58.37 [54.19, 62.74]	91	59.31 [52.99, 62.59]	NA	59.31 [52.99, 62.59]	90.2
**RVPER (median [IQR])**	388.72 [316.56, 465.82]	446.55 [446.55, 446.55]	388.66 [316.54, 465.97]	91	361.23 [290.07, 443.94]	NA	361.23 [290.07, 443.94]	90.2
**RVPFR (median [IQR])**	300.61 [245.17, 364.00]	373.24 [373.24, 373.24]	300.34 [245.12, 363.44]	91	295.78 [220.42, 343.17]	NA	295.78 [220.42, 343.17]	90.2
**RVPAFR (median [IQR])**	282.95 [222.68, 360.32]	547.72 [547.72, 547.72]	282.86 [222.56, 360.07]	91	275.01 [224.94, 344.91]	NA	275.01 [224.94, 344.91]	90.2
**LVEDVi (median [IQR])**	74.33 [66.34, 83.11]	64.68 [59.39, 69.33]	74.37 [66.38, 83.15]	91.9	77.32 [68.06, 86.15]	NA	77.32 [68.06, 86.15]	91.4
**LVESVi (median [IQR])**	30.02 [25.12, 35.70]	26.43 [25.24, 28.39]	30.02 [25.13, 35.72]	91.9	31.69 [26.19, 39.84]	NA	31.69 [26.19, 39.84]	91.4
**LVSVi (median [IQR])**	44.03 [39.34, 50.28]	38.25 [31.00, 44.09]	44.05 [39.37, 50.30]	91.9	43.18 [37.50, 49.11]	NA	43.18 [37.50, 49.11]	91.4
**LVEF (median [IQR])**	59.47 [55.29, 63.52]	59.14 [51.51, 63.31]	59.48 [55.29, 63.52]	91.9	57.34 [52.60, 62.80]	NA	57.34 [52.60, 62.80]	91.4
**LVPER (median [IQR])**	373.80 [302.29, 452.71]	284.40 [256.83, 364.56]	373.81 [302.44, 453.27]	91.9	339.21 [258.97, 430.85]	NA	339.21 [258.97, 430.85]	91.4
**LVPFR (median [IQR])**	321.31 [259.23, 385.16]	201.74 [189.85, 229.92]	321.49 [259.95, 385.63]	91.9	314.20 [258.81, 366.77]	NA	314.20 [258.81, 366.77]	91.4
**LVPAFR (median [IQR])**	233.66 [167.35, 306.30]	363.87 [208.97, 466.86]	233.50 [167.46, 305.08]	91.9	253.35 [178.90, 330.36]	NA	253.35 [178.90, 330.36]	91.4
**LVEDMi (median [IQR])**	41.88 [36.52, 48.62]	45.76 [34.02, 48.75]	41.85 [36.55, 48.61]	91.9	42.96 [36.56, 46.70]	NA	42.96 [36.56, 46.70]	91.4
**LVMVR (median [IQR])**	0.56 [0.50, 0.62]	0.70 [0.56, 0.70]	0.56 [0.50, 0.62]	91.9	0.54 [0.49, 0.59]	NA	0.54 [0.49, 0.59]	91.4
**LVEDV/RVEDV (median [IQR])**	0.93 [0.86, 1.03]	0.93 [0.93, 0.93]	0.93 [0.86, 1.03]	92.2	1.00 [0.91, 1.08]	NA	1.00 [0.91, 1.08]	91.8
**LVESV/RVESV (median [IQR])**	0.91 [0.80, 1.04]	1.15 [1.15, 1.15]	0.91 [0.80, 1.04]	92.2	1.02 [0.89, 1.19]	NA	1.02 [0.89, 1.19]	91.8
**peakEcc (median [IQR])**	-22.70 [-24.98, -20.43]	-21.60 [-21.61, -21.59]	-22.72 [-24.98, -20.42]	93.7	-22.67 [-24.40, -19.13]	NA	-22.67 [-24.40, -19.13]	92.8
**TPKEcc (median [IQR])**	331.31 [309.83, 354.66]	325.97 [321.27, 330.67]	331.31 [309.75, 354.68]	93.7	334.71 [320.31, 360.48]	NA	334.71 [320.31, 360.48]	92.8
**peakEll2Ch (median [IQR])**	-21.19 [-23.34, -18.93]	-23.89 [-23.89, -23.89]	-21.17 [-23.32, -18.93]	93.8	-20.29 [-22.24, -17.98]	NA	-20.29 [-22.24, -17.98]	93
**TPKEll2Ch (median [IQR])**	349.86 [321.84, 379.10]	388.50 [388.50, 388.50]	349.86 [321.81, 379.08]	93.9	353.29 [331.00, 381.68]	NA	353.29 [331.00, 381.68]	93
**peakEll4Ch (median [IQR])**	-23.30 [-25.97, -21.37]	-21.40 [-21.40, -21.40]	-23.30 [-25.99, -21.37]	93.9	-22.30 [-24.57, -19.76]	NA	-22.30 [-24.57, -19.76]	93
**TPKEll4Ch (median [IQR])**	357.53 [327.07, 397.64]	360.78 [360.78, 360.78]	357.30 [326.96, 397.81]	93.9	354.80 [325.41, 392.55]	NA	354.80 [325.41, 392.55]	93.1
**Wall thickness segment 1 (median [IQR])**	7.65 [6.81, 8.50]	7.04 [5.94, 8.14]	7.65 [6.81, 8.49]	93.2	7.44 [6.78, 8.21]	NA	7.44 [6.78, 8.21]	93.4
**Wall thickness segment 2 (median [IQR])**	6.75 [5.75, 7.91]	7.50 [6.70, 8.31]	6.75 [5.74, 7.90]	93.2	6.03 [5.31, 7.39]	NA	6.03 [5.31, 7.39]	93.4
**Wall thickness segment 3 (median [IQR])**	6.05 [5.17, 6.96]	7.60 [6.78, 8.42]	6.05 [5.17, 6.95]	93.2	6.10 [4.85, 6.66]	NA	6.10 [4.85, 6.66]	93.4
**Wall thickness segment 4 (median [IQR])**	6.54 [5.82, 7.22]	8.31 [7.66, 8.97]	6.54 [5.81, 7.21]	93.2	6.57 [5.89, 6.99]	NA	6.57 [5.89, 6.99]	93.4
**Wall thickness segment 5 (median [IQR])**	6.20 [5.62, 6.96]	6.33 [5.27, 7.39]	6.20 [5.62, 6.96]	93.2	6.08 [5.57, 6.50]	NA	6.08 [5.57, 6.50]	93.4
**Wall thickness segment 6 (median [IQR])**	6.55 [5.97, 7.31]	6.61 [5.42, 7.79]	6.55 [5.98, 7.31]	93.2	6.58 [6.05, 7.01]	NA	6.58 [6.05, 7.01]	93.4
**Wall thickness segment 7 (median [IQR])**	5.73 [5.28, 6.31]	5.48 [4.90, 6.07]	5.73 [5.29, 6.31]	93.2	5.56 [5.28, 6.14]	NA	5.56 [5.28, 6.14]	93.4
**Wall thickness segment 8 (median [IQR])**	7.01 [6.28, 7.76]	8.52 [7.77, 9.27]	7.01 [6.28, 7.75]	93.2	6.90 [6.27, 7.47]	NA	6.90 [6.27, 7.47]	93.4
**Wall thickness segment 9 (median [IQR])**	7.38 [6.48, 8.25]	9.47 [8.05, 10.89]	7.38 [6.48, 8.25]	93.2	7.18 [6.64, 7.85]	NA	7.18 [6.64, 7.85]	93.4
**Wall thickness segment 10 (median [IQR])**	6.23 [5.59, 6.96]	7.07 [6.08, 8.06]	6.23 [5.60, 6.96]	93.2	6.28 [5.90, 6.89]	NA	6.28 [5.90, 6.89]	93.4
**Wall thickness segment 11 (median [IQR])**	5.62 [5.09, 6.32]	5.57 [5.19, 5.95]	5.62 [5.10, 6.32]	93.2	5.56 [5.07, 6.08]	NA	5.56 [5.07, 6.08]	93.4
**Wall thickness segment 12 (median [IQR])**	5.60 [5.22, 6.25]	5.56 [5.53, 5.60]	5.60 [5.22, 6.26]	93.2	5.50 [5.14, 6.02]	NA	5.50 [5.14, 6.02]	93.4
**Wall thickness segment 13 (median [IQR])**	5.48 [5.10, 5.91]	8.01 [7.03, 9.00]	5.48 [5.10, 5.91]	93.2	5.44 [5.13, 5.91]	NA	5.44 [5.13, 5.91]	93.4
**Wall thickness segment 14 (median [IQR])**	6.00 [5.36, 6.66]	8.33 [6.87, 9.79]	6.00 [5.36, 6.66]	93.2	5.90 [5.44, 6.28]	NA	5.90 [5.44, 6.28]	93.4
**Wall thickness segment 15 (median [IQR])**	5.09 [4.48, 5.69]	7.21 [5.80, 8.63]	5.09 [4.48, 5.69]	93.2	4.99 [4.56, 5.40]	NA	4.99 [4.56, 5.40]	93.4
**Wall thickness segment 16 (median [IQR])**	5.26 [4.80, 5.72]	8.31 [6.87, 9.75]	5.26 [4.80, 5.72]	93.2	5.13 [4.82, 5.67]	NA	5.13 [4.82, 5.67]	93.4
**Global wall thickness (median [IQR])**	6.30 [5.73, 6.84]	7.33 [7.28, 7.38]	6.30 [5.72, 6.84]	93.2	6.17 [5.90, 6.59]	NA	6.17 [5.90, 6.59]	93.4
**Septal wall thickness (median [IQR])**	6.68 [5.88, 7.35]	8.28 [7.88, 8.69]	6.67 [5.88, 7.34]	93.2	6.37 [6.00, 6.90]	NA	6.37 [6.00, 6.90]	93.4
**Maximum wall thickness (median [IQR])**	8.10 [7.24, 9.02]	10.96 [10.29, 11.64]	8.09 [7.24, 9.01]	93.2	8.01 [7.43, 8.66]	NA	8.01 [7.43, 8.66]	93.4

Abbreviations:
ARVC: arrythmogenic right ventricular cardiomyopathy; BMI: body mass index; CMR: cardiac magnetic resonance imaging; DCM: dilated cardiomyopathy; ECG: Electrocardiography; EDVi: indexed end-diastolic volume; EDMi: indexed end-diastolic mass; EF: ejection fraction; ESVi: indexed end-systolic volume; G+: carriers of likely pathogenic and pathogenic variants associated with one of the cardiomyopathies; HCM: hypertrophic cardiomyopathy; HDL: high-density lipoprotein; IQR: interquartile range; LDL: low-density lipoprotein; LV: left ventricular; MET: metabolic equivalent of task; MVR: mass to volume ratio; PAFR: peak atrial filling rate; peakEcc: peak circumferential strain; peakEll2Ch: longitudinal strain analyzed in 2-chamber view; peakEll4Ch: longitudinal strain analyzed in 4-chamber view; PER: peak ejection rate; PFR: peak filling rate; RV: right ventricular; SVi: indexed stroke volume; TPKEcc: global time to peak circumferential strain; TPKEll2Ch: global time to longitudinal strain analyzed in 2-chamber view; TPKEll4Ch: global time to longitudinal strain analyzed in 4-chamber view.

Supplementary Table III: Extensive baseline table

	Controls G-				HCM G+			
	**Overall**	**Diagnosed**	**Non-Diagnosed**	**Missing**	**Overall**	**Diagnosed**	**Non-Diagnosed**	**Missing**
n (%)	9,972 (100)	87 (0.8)	9,885 (99.2)		1,346 (100)	35 (2.6)	1,311 (97.4)	
**Sex = Female (%)**	5,436 (54.5)	35 (40.2)	5,401 (54.6)	0	720 (53.5)	20 (57.1)	700 (53.4)	0
**Age (median [IQR])**	57.00 [49.00, 63.00]	62.00 [56.00, 66.00]	57.00 [49.00, 63.00]	0	56.00 [49.00, 63.00]	59.00 [51.00, 66.00]	56.00 [49.00, 63.00]	0
**Ethnicity (%)**				1				1.3
Asian	1,076 (10.9)	5 (5.8)	1071 (10.9)		251 (18.9)	3 (8.6)	248 (19.2)	
Black	164 (1.7)	3 (3.5)	161 (1.6)		22 (1.7)	0 (0.0)	22 (1.7)	
Chinese	56 (0.6)	1 (1.2)	55 (0.6)		1 (0.1)	0 (0.0)	1 (0.1)	
Mixed	132 (1.3)	2 (2.3)	130 (1.3)		28 (2.1)	0 (0.0)	28 (2.2)	
Other	160 (1.6)	1 (1.2)	159 (1.6)		26 (2.0)	0 (0.0)	26 (2.0)	
White	8,288 (83.9)	74 (86.0)	8,214 (83.9)		1001 (75.3)	32 (91.4)	969 (74.9)	
**CARDIOVASCULAR RISK FACTORS**								
**BMI (median [IQR])**	26.73 [24.15, 29.82]	28.83 [25.33, 32.20]	26.71 [24.15, 29.80]	0.5	26.56 [23.88, 29.76]	26.54 [23.30, 31.10]	26.56 [23.90, 29.72]	1
**Diabetes (%)**	914 (9.2)	21 (24.1)	893 (9.0)	0	154 (11.4)	4 (11.4)	150 (11.4)	0
**Hypertension (%)**	3,420 (34.3)	60 (69.0)	3,360 (34.0)	0	475 (35.3)	23 (65.7)	452 (34.5)	0
**Mean systolic blood pressure (median [IQR])**	135.50 [124.00, 149.00]	144.00 [130.00, 155.50]	135.50 [124.00, 149.00]	0.1	135.00 [123.50, 148.50]	135.50 [125.00, 154.25]	135.00 [123.50, 148.00]	0.2
**Mean diastolic blood pressure (median [IQR])**	82.00 [75.00, 88.50]	84.00 [76.50, 91.50]	81.50 [75.00, 88.50]	0.1	81.50 [75.50, 89.00]	81.50 [72.50, 91.25]	81.50 [75.50, 89.00]	0.2
**Hypercholesterolaemia (%)**	2,416 (24.2)	34 (39.1)	2,382 (24.1)	0	369 (27.4)	12 (34.3)	357 (27.2)	0
**Total cholesterol (median [IQR])**	5.61 [4.86, 6.38]	5.23 [4.39, 6.10]	5.61 [4.87, 6.38]	4.3	5.60 [4.80, 6.38]	5.58 [4.73, 6.46]	5.60 [4.80, 6.37]	4.5
**HDL (median [IQR])**	1.38 [1.16, 1.65]	1.29 [1.13, 1.56]	1.38 [1.16, 1.65]	11.3	1.37 [1.14, 1.63]	1.32 [1.23, 1.58]	1.37 [1.14, 1.63]	12.1
**LDL (median [IQR])**	3.50 [2.92, 4.09]	3.22 [2.54, 3.81]	3.50 [2.92, 4.09]	4.6	3.48 [2.87, 4.09]	3.38 [2.67, 4.43]	3.48 [2.88, 4.07]	4.8
**Ever Smoked (%)**	4,132 (41.4)	50 (57.5)	4,082 (41.3)	0	543 (40.3)	12 (34.3)	531 (40.5)	0
**MET minutes per week for walking (median [IQR])**	693.00 [297.00, 1,386.00]	528.00 [255.75, 1,608.75]	693.00 [297.00, 1,386.00]	19.8	693.00 [307.72, 1,386.00]	495.00 [198.00, 1,386.00]	693.00 [330.00, 1,386.00]	19.6
**MET minutes per week for moderate activity (median [IQR])**	480.00 [120.00, 1,200.00]	480.00 [100.00, 1,680.00]	480.00 [120.00, 1,200.00]	19.8	480.00 [120.00, 1,200.00]	400.00 [160.00, 840.00]	480.00 [120.00, 1,200.00]	19.6
**MET minutes per week for vigorous activity (median [IQR])**	240.00 [0.00, 960.00]	0.00 [0.00, 820.00]	240.00 [0.00, 960.00]	19.8	160.00 [0.00, 930.00]	240.00 [0.00, 480.00]	160.00 [0.00, 960.00]	19.6
**Total MET minutes per week (median [IQR])**	1,773.00 [810.00, 3,452.50]	1,367.50 [478.50, 3,834.00]	1,776.50 [810.00, 3,450.00]	19.8	1,762.00 [848.12, 3,490.25]	1,253.00 [693.00, 3,426.00]	1,773.00 [853.00, 3,492.00]	19.6
**Family heart disease (%)**	4,458 (44.7)	34 (39.1)	4,424 (44.8)	0	623 (46.3)	15 (42.9)	608 (46.4)	0
**CARDIAC DISEASES/OUTCOMES**								
**Cardiac problem (%)**	41 (0.4)	2 (2.3)	39 (0.4)	0	5 (0.4)	0 (0.0)	5 (0.4)	0
**Heart failure (%)**	182 (1.8)	74 (85.1)	108 (1.1)	0	33 (2.5)	15 (42.9)	18 (1.4)	0
**Cardiomyopathy (%)**	37 (0.4)	26 (29.9)	11 (0.1)	0	27 (2.0)	21 (60.0)	6 (0.5)	0
**Dilated cardiomyopathy (%)**	14 (0.1)	8 (9.2)	6 (0.1)	0	1 (0.1)	1 (2.9)	0 (0.0)	0
**Hypertrophic cardiomyopathy (%)**	8 (0.1)	7 (8.0)	1 (0.0)	0	20 (1.5)	14 (40.0)	6 (0.5)	0
**Ventricular arrhythmias (%)**	33 (0.3)	3 (3.4)	30 (0.3)	0	8 (0.6)	4 (11.4)	4 (0.3)	0
**Atrial arrhythmias (%)**	191 (1.9)	19 (21.8)	172 (1.7)	0	32 (2.4)	6 (17.1)	26 (2.0)	0
**Heart arrhythmia (%)**	54 (0.5)	2 (2.3)	52 (0.5)	0	4 (0.3)	0 (0.0)	4 (0.3)	0
**Chronic ischemic heart disease (%)**	725 (7.3)	0 (0.0)	725 (7.3)	0	93 (6.9)	0 (0.0)	93 (7.1)	0
**Acute myocardial infarction (%)**	298 (3.0)	1 (1.1)	297 (3.0)	0	36 (2.7)	2 (5.7)	34 (2.6)	0
**Cardiac arrest (%)**	34 (0.3)	1 (1.1)	33 (0.3)	0	5 (0.4)	0 (0.0)	5 (0.4)	0
**Angina pectoris (%)**	312 (3.1)	2 (2.3)	310 (3.1)	0	56 (4.2)	0 (0.0)	56 (4.3)	0
**Conduction disorders (%)**	151 (1.5)	10 (11.5)	141 (1.4)	0	26 (1.9)	6 (17.1)	20 (1.5)	0
**Valvular disease (%)**	241 (2.4)	23 (26.4)	218 (2.2)	0	41 (3.0)	8 (22.9)	33 (2.5)	0
**Congenital heart disease (%)**	28 (0.3)	3 (3.4)	25 (0.3)	0	4 (0.3)	0 (0.0)	4 (0.3)	0
**Pulmonary obstructive disease (%)**	494 (5.0)	24 (27.6)	470 (4.8)	0	57 (4.2)	4 (11.4)	53 (4.0)	0
**Cardiovascular death (%)**	181 (1.8)	13 (14.9)	168 (1.7)	0	18 (1.3)	3 (8.6)	15 (1.1)	0
**All-cause mortality (%)**	513 (5.1)	27 (31.0)	486 (4.9)	0	62 (4.6)	6 (17.1)	56 (4.3)	0
**ECG MEASUREMENTS**								
**n (%)**	1,062 (10.6)	4 (4.6)	1,058 (10.7)		138 (10.3)	5 (14.3)	133 (10.1)	
**P duration (median [IQR])**	100.00 [90.00, 108.00]	90.00 [82.00, 111.00]	100.00 [90.00, 108.00]	89.8	100.00 [90.00, 108.00]	100.00 [86.00, 102.00]	100.00 [90.00, 108.00]	90
**P axis (median [IQR])**	55.00 [40.25, 67.00]	47.00 [47.00, 47.00]	55.00 [40.00, 67.00]	93.1	53.00 [36.00, 63.00]	70.00 [66.00, 70.00]	52.00 [36.00, 62.00]	92.9
**PQ interval (median [IQR])**	160.00 [145.50, 178.00]	188.00 [188.00, 188.00]	160.00 [145.00, 178.00]	93.1	161.00 [147.50, 172.50]	190.00 [186.00, 199.00]	160.00 [146.00, 172.00]	92.9
**QRS duration (median [IQR])**	86.00 [80.00, 94.00]	93.00 [89.50, 97.00]	86.00 [80.00, 94.00]	89.4	84.00 [80.00, 93.50]	100.00 [82.00, 104.00]	84.00 [80.00, 92.00]	89.7
**R axis (median [IQR])**	34.00 [7.00, 58.00]	-48.00 [-48.00, -48.00]	34.00 [7.50, 58.00]	92.9	39.00 [10.00, 52.75]	2.00 [-17.00, 24.00]	39.00 [11.50, 53.50]	92.7
**QTC interval (median [IQR])**	417.00 [402.00, 433.00]	511.00 [511.00, 511.00]	417.00 [402.00, 432.50]	92.9	414.50 [402.00, 429.00]	435.00 [428.50, 456.50]	414.00 [402.00, 428.50]	92.7
**T axis (median [IQR])**	40.00 [23.00, 55.25]	92.00 [92.00, 92.00]	40.00 [23.00, 55.00]	92.9	45.00 [31.25, 60.75]	49.00 [43.00, 96.00]	45.00 [30.50, 60.50]	92.7
**CMR MEASUREMENTS**								
**n (%)**	990 (9.9)	4 (4.6)	986 (10.0)		134 (10.0)	4 (11.4)	130 (9.9)	
**RVEDVi (median [IQR])**	80.18 [70.62, 90.27]	79.28 [79.28, 79.28]	80.19 [70.61, 90.27]	91	77.30 [67.73, 90.71]	79.56 [77.34, 90.20]	77.12 [67.27, 90.71]	90.4
**RVESVi (median [IQR])**	32.89 [27.38, 39.68]	20.93 [20.93, 20.93]	32.90 [27.42, 39.70]	91	31.72 [26.22, 37.33]	34.80 [30.85, 43.04]	31.40 [26.20, 37.28]	90.4
**RVSVi (median [IQR])**	46.55 [40.92, 52.84]	58.35 [58.35, 58.35]	46.53 [40.91, 52.81]	91	46.03 [40.64, 54.04]	47.41 [45.02, 51.27]	45.77 [40.64, 54.04]	90.4
**RVEF (median [IQR])**	58.38 [54.19, 62.76]	73.60 [73.60, 73.60]	58.37 [54.19, 62.74]	91	59.56 [54.73, 63.95]	56.21 [51.68, 60.89]	59.56 [54.80, 63.99]	90.5
**RVPER (median [IQR])**	388.72 [316.56, 465.82]	446.55 [446.55, 446.55]	388.66 [316.54, 465.97]	91	389.50 [310.19, 475.79]	391.94 [296.40, 448.92]	389.50 [310.19, 475.79]	90.4
**RVPFR (median [IQR])**	300.61 [245.17, 364.00]	373.24 [373.24, 373.24]	300.34 [245.12, 363.44]	91	285.88 [232.85, 336.38]	363.12 [334.18, 386.47]	278.68 [230.83, 334.67]	90.4
**RVPAFR (median [IQR])**	282.95 [222.68, 360.32]	547.72 [547.72, 547.72]	282.86 [222.56, 360.07]	91	299.05 [236.43, 365.16]	263.66 [225.50, 297.24]	300.15 [236.43, 366.42]	90.4
**LVEDVi (median [IQR])**	74.33 [66.34, 83.11]	64.68 [59.39, 69.33]	74.37 [66.38, 83.15]	91.9	74.50 [64.57, 84.89]	85.13 [83.78, 87.08]	72.35 [64.32, 84.59]	91.2
**LVESVi (median [IQR])**	30.02 [25.12, 35.70]	26.43 [25.24, 28.39]	30.02 [25.13, 35.72]	91.9	30.31 [24.10, 35.20]	43.58 [39.30, 47.19]	29.37 [24.09, 34.83]	91.2
**LVSVi (median [IQR])**	44.03 [39.34, 50.28]	38.25 [31.00, 44.09]	44.05 [39.37, 50.30]	91.9	44.17 [38.21, 49.98]	44.56 [42.26, 45.10]	44.07 [38.21, 50.19]	91.2
**LVEF (median [IQR])**	59.47 [55.29, 63.52]	59.14 [51.51, 63.31]	59.48 [55.29, 63.52]	91.9	59.55 [55.96, 63.53]	51.02 [47.77, 53.26]	59.73 [56.25, 63.62]	91.2
**LVPER (median [IQR])**	373.80 [302.29, 452.71]	284.40 [256.83, 364.56]	373.81 [302.44, 453.27]	91.9	340.46 [266.40, 459.49]	350.17 [311.46, 380.66]	340.46 [264.60, 460.79]	91.2
**LVPFR (median [IQR])**	321.31 [259.23, 385.16]	201.74 [189.85, 229.92]	321.49 [259.95, 385.63]	91.9	320.26 [252.40, 371.17]	315.79 [273.86, 360.98]	320.26 [248.52, 371.17]	91.2
**LVPAFR (median [IQR])**	233.66 [167.35, 306.30]	363.87 [208.97, 466.86]	233.50 [167.46, 305.08]	91.9	241.27 [165.31, 316.89]	213.93 [135.78, 272.60]	241.27 [167.29, 321.53]	91.2
**LVEDMi (median [IQR])**	41.88 [36.52, 48.62]	45.76 [34.02, 48.75]	41.85 [36.55, 48.61]	91.9	42.87 [35.12, 49.47]	49.63 [46.84, 53.17]	42.41 [34.89, 49.27]	91.2
**LVMVR (median [IQR])**	0.56 [0.50, 0.62]	0.70 [0.56, 0.70]	0.56 [0.50, 0.62]	91.9	0.56 [0.50, 0.64]	0.58 [0.55, 0.61]	0.56 [0.50, 0.64]	91.2
**LVEDV/RVEDV (median [IQR])**	0.93 [0.86, 1.03]	0.93 [0.93, 0.93]	0.93 [0.86, 1.03]	92.2	0.94 [0.86, 1.03]	1.07 [0.99, 1.08]	0.94 [0.86, 0.99]	91.3
**LVESV/RVESV (median [IQR])**	0.91 [0.80, 1.04]	1.15 [1.15, 1.15]	0.91 [0.80, 1.04]	92.2	0.90 [0.82, 1.05]	1.28 [1.14, 1.30]	0.90 [0.82, 1.02]	91.3
**peakEcc (median [IQR])**	-22.70 [-24.98, -20.43]	-21.60 [-21.61, -21.59]	-22.72 [-24.98, -20.42]	93.7	-22.82 [-24.98, -20.60]	-20.32 [-20.51, -20.27]	-22.91 [-25.19, -20.88]	93.5
**TPKEcc (median [IQR])**	331.31 [309.83, 354.66]	325.97 [321.27, 330.67]	331.31 [309.75, 354.68]	93.7	332.34 [308.14, 354.02]	347.73 [320.45, 364.22]	331.96 [308.15, 353.59]	93.5
**peakEll2Ch (median [IQR])**	-21.19 [-23.34, -18.93]	-23.89 [-23.89, -23.89]	-21.17 [-23.32, -18.93]	93.8	-20.99 [-23.36, -19.01]	-20.63 [-20.99, -20.19]	-21.54 [-23.50, -18.88]	93.5
**TPKEll2Ch (median [IQR])**	349.86 [321.84, 379.10]	388.50 [388.50, 388.50]	349.86 [321.81, 379.08]	93.9	355.54 [320.85, 381.68]	403.20 [386.90, 420.80]	353.10 [320.40, 379.60]	93.5
**peakEll4Ch (median [IQR])**	-23.30 [-25.97, -21.37]	-21.40 [-21.40, -21.40]	-23.30 [-25.99, -21.37]	93.9	-23.82 [-26.50, -21.90]	-20.87 [-22.20, -19.79]	-24.05 [-26.93, -22.28]	94.1
**TPKEll4Ch (median [IQR])**	357.53 [327.07, 397.64]	360.78 [360.78, 360.78]	357.30 [326.96, 397.81]	93.9	350.13 [318.54, 395.01]	403.22 [378.52, 407.61]	349.20 [318.54, 390.16]	94.1
**Wall thickness segment 1 (median [IQR])**	7.65 [6.81, 8.50]	7.04 [5.94, 8.14]	7.65 [6.81, 8.49]	93.2	7.59 [6.74, 8.63]	9.35 [9.26, 9.44]	7.59 [6.72, 8.55]	92.9
**Wall thickness segment 2 (median [IQR])**	6.75 [5.75, 7.91]	7.50 [6.70, 8.31]	6.75 [5.74, 7.90]	93.2	7.10 [5.91, 8.26]	9.50 [8.42, 10.57]	7.06 [5.87, 8.21]	92.9
**Wall thickness segment 3 (median [IQR])**	6.05 [5.17, 6.96]	7.60 [6.78, 8.42]	6.05 [5.17, 6.95]	93.2	6.23 [5.07, 7.49]	7.99 [7.50, 8.47]	6.22 [5.04, 7.39]	92.9
**Wall thickness segment 4 (median [IQR])**	6.54 [5.82, 7.22]	8.31 [7.66, 8.97]	6.54 [5.81, 7.21]	93.2	6.49 [5.86, 6.99]	7.25 [7.12, 7.38]	6.47 [5.85, 6.92]	92.9
**Wall thickness segment 5 (median [IQR])**	6.20 [5.62, 6.96]	6.33 [5.27, 7.39]	6.20 [5.62, 6.96]	93.2	6.03 [5.57, 6.65]	6.36 [6.00, 6.72]	6.03 [5.57, 6.64]	92.9
**Wall thickness segment 6 (median [IQR])**	6.55 [5.97, 7.31]	6.61 [5.42, 7.79]	6.55 [5.98, 7.31]	93.2	6.56 [5.94, 7.13]	6.72 [6.45, 6.98]	6.56 [5.93, 7.09]	92.9
**Wall thickness segment 7 (median [IQR])**	5.73 [5.28, 6.31]	5.48 [4.90, 6.07]	5.73 [5.29, 6.31]	93.2	5.75 [5.22, 6.20]	6.03 [5.92, 6.13]	5.75 [5.21, 6.19]	92.9
**Wall thickness segment 8 (median [IQR])**	7.01 [6.28, 7.76]	8.52 [7.77, 9.27]	7.01 [6.28, 7.75]	93.2	6.89 [6.17, 7.50]	8.76 [7.66, 9.86]	6.89 [6.16, 7.49]	92.9
**Wall thickness segment 9 (median [IQR])**	7.38 [6.48, 8.25]	9.47 [8.05, 10.89]	7.38 [6.48, 8.25]	93.2	7.21 [6.38, 8.22]	8.99 [7.76, 10.21]	7.21 [6.38, 8.20]	92.9
**Wall thickness segment 10 (median [IQR])**	6.23 [5.59, 6.96]	7.07 [6.08, 8.06]	6.23 [5.60, 6.96]	93.2	6.18 [5.45, 7.05]	6.48 [6.17, 6.78]	6.18 [5.44, 7.00]	92.9
**Wall thickness segment 11 (median [IQR])**	5.62 [5.09, 6.32]	5.57 [5.19, 5.95]	5.62 [5.10, 6.32]	93.2	5.53 [5.04, 6.33]	5.75 [5.62, 5.88]	5.53 [5.02, 6.37]	92.9
**Wall thickness segment 12 (median [IQR])**	5.60 [5.22, 6.25]	5.56 [5.53, 5.60]	5.60 [5.22, 6.26]	93.2	5.59 [5.10, 6.11]	5.52 [5.32, 5.73]	5.59 [5.08, 6.13]	92.9
**Wall thickness segment 13 (median [IQR])**	5.48 [5.10, 5.91]	8.01 [7.03, 9.00]	5.48 [5.10, 5.91]	93.2	5.49 [5.10, 5.94]	5.21 [4.61, 5.82]	5.49 [5.12, 5.93]	92.9
**Wall thickness segment 14 (median [IQR])**	6.00 [5.36, 6.66]	8.33 [6.87, 9.79]	6.00 [5.36, 6.66]	93.2	5.95 [5.38, 6.60]	6.05 [5.36, 6.74]	5.95 [5.38, 6.58]	92.9
**Wall thickness segment 15 (median [IQR])**	5.09 [4.48, 5.69]	7.21 [5.80, 8.63]	5.09 [4.48, 5.69]	93.2	4.99 [4.45, 5.69]	5.30 [5.05, 5.54]	4.99 [4.44, 5.68]	92.9
**Wall thickness segment 16 (median [IQR])**	5.26 [4.80, 5.72]	8.31 [6.87, 9.75]	5.26 [4.80, 5.72]	93.2	5.23 [4.75, 5.68]	5.32 [4.77, 5.87]	5.23 [4.76, 5.65]	92.9
**Global wall thickness (median [IQR])**	6.30 [5.73, 6.84]	7.33 [7.28, 7.38]	6.30 [5.72, 6.84]	93.2	6.30 [5.74, 6.92]	7.06 [6.95, 7.17]	6.28 [5.70, 6.87]	92.9
**Septal wall thickness (median [IQR])**	6.68 [5.88, 7.35]	8.28 [7.88, 8.69]	6.67 [5.88, 7.34]	93.2	6.65 [6.13, 7.61]	8.26 [7.96, 8.55]	6.62 [6.12, 7.52]	92.9
**Maximum wall thickness (median [IQR])**	8.10 [7.24, 9.02]	10.96 [10.29, 11.64]	8.09 [7.24, 9.01]	93.2	8.19 [7.46, 9.65]	11.54 [11.49, 11.59]	8.16 [7.45, 9.58]	92.9

Abbreviations:
ARVC: arrythmogenic right ventricular cardiomyopathy; BMI: body mass index; CMR: cardiac magnetic resonance imaging; DCM: dilated cardiomyopathy; ECG: Electrocardiography; EDVi: indexed end-diastolic volume; EDMi: indexed end-diastolic mass; EF: ejection fraction; ESVi: indexed end-systolic volume; G+: carriers of likely pathogenic and pathogenic variants associated with one of the cardiomyopathies; HCM: hypertrophic cardiomyopathy; HDL: high-density lipoprotein; IQR: interquartile range; LDL: low-density lipoprotein; LV: left ventricular; MET: metabolic equivalent of task; MVR: mass to volume ratio; PAFR: peak atrial filling rate; peakEcc: peak circumferential strain; peakEll2Ch: longitudinal strain analyzed in 2-chamber view; peakEll4Ch: longitudinal strain analyzed in 4-chamber view; PER: peak ejection rate; PFR: peak filling rate; RV: right ventricular; SVi: indexed stroke volume; TPKEcc: global time to peak circumferential strain; TPKEll2Ch: global time to longitudinal strain analyzed in 2-chamber view; TPKEll4Ch: global time to longitudinal strain analyzed in 4-chamber view.

Supplementary Table III: Extensive baseline table								
	Controls G-				strict HCM G+			
	Overall	Diagnosed	Non-Diagnosed	Missing	Overall	Diagnosed	Non-Diagnosed	Missing
n (%)	9,972 (100)	87 (0.8)	9,885 (99.2)		801 (100)	32 (4.0)	769 (96.0)	
**Sex = Female (%)**	5,436 (54.5)	35 (40.2)	5,401 (54.6)	0	445 (55.6)	19 (59.4)	426 (55.4)	0
**Age (median [IQR])**	57.00 [49.00, 63.00]	62.00 [56.00, 66.00]	57.00 [49.00, 63.00]	0	58.00 [50.00, 63.00]	60.00 [52.75, 66.00]	58.00 [50.00, 63.00]	0
**Ethnicity (%)**				1				0.6
Asian	1,076 (10.9)	5 (5.8)	1071 (10.9)		14 (1.8)	0 (0.0)	14 (1.8)	
Black	164 (1.7)	3 (3.5)	161 (1.6)		18 (2.3)	0 (0.0)	18 (2.4)	
Chinese	56 (0.6)	1 (1.2)	55 (0.6)		1 (0.1)	0 (0.0)	1 (0.1)	
Mixed	132 (1.3)	2 (2.3)	130 (1.3)		6 (0.8)	0 (0.0)	6 (0.8)	
Other	160 (1.6)	1 (1.2)	159 (1.6)		3 (0.4)	0 (0.0)	3 (0.4)	
White	8,288 (83.9)	74 (86.0)	8,214 (83.9)		754 (94.7)	32 (100.0)	722 (94.5)	
CARDIOVASCULAR RISK FACTORS								
**BMI (median [IQR])**	26.73 [24.15, 29.82]	28.83 [25.33, 32.20]	26.71 [24.15, 29.80]	0.5	26.56 [23.84, 29.85]	26.25 [23.37, 30.57]	26.58 [23.85, 29.79]	0.4
**Diabetes (%)**	914 (9.2)	21 (24.1)	893 (9.0)	0	60 (7.5)	3 (9.4)	57 (7.4)	0
**Hypertension (%)**	3,420 (34.3)	60 (69.0)	3,360 (34.0)	0	291 (36.3)	21 (65.6)	270 (35.1)	0
**Mean systolic blood pressure (median [IQR])**	135.50 [124.00, 149.00]	144.00 [130.00, 155.50]	135.50 [124.00, 149.00]	0.1	136.00 [123.50, 150.50]	138.50 [128.12, 156.50]	136.00 [123.50, 150.00]	0.1
**Mean diastolic blood pressure (median [IQR])**	82.00 [75.00, 88.50]	84.00 [76.50, 91.50]	81.50 [75.00, 88.50]	0.1	81.50 [75.00, 89.00]	83.00 [74.88, 92.12]	81.50 [75.00, 89.00]	0.1
**Hypercholesterolaemia (%)**	2,416 (24.2)	34 (39.1)	2,382 (24.1)	0	195 (24.3)	11 (34.4)	184 (23.9)	0
**Total cholesterol (median [IQR])**	5.61 [4.86, 6.38]	5.23 [4.39, 6.10]	5.61 [4.87, 6.38]	4.3	5.63 [4.86, 6.42]	5.75 [5.03, 6.51]	5.63 [4.85, 6.41]	4.6
**HDL (median [IQR])**	1.38 [1.16, 1.65]	1.29 [1.13, 1.56]	1.38 [1.16, 1.65]	11.3	1.40 [1.18, 1.68]	1.33 [1.24, 1.64]	1.40 [1.18, 1.68]	13
**LDL (median [IQR])**	3.50 [2.92, 4.09]	3.22 [2.54, 3.81]	3.50 [2.92, 4.09]	4.6	3.50 [2.90, 4.11]	3.47 [2.98, 4.46]	3.50 [2.90, 4.10]	4.9
**Ever Smoked (%)**	4,132 (41.4)	50 (57.5)	4,082 (41.3)	0	361 (45.1)	11 (34.4)	350 (45.5)	0
**MET minutes per week for walking (median [IQR])**	693.00 [297.00, 1,386.00]	528.00 [255.75, 1,608.75]	693.00 [297.00, 1,386.00]	19.8	693.00 [330.00, 1,386.00]	495.00 [214.50, 1,386.00]	693.00 [330.00, 1,386.00]	18
**MET minutes per week for moderate activity (median [IQR])**	480.00 [120.00, 1,200.00]	480.00 [100.00, 1,680.00]	480.00 [120.00, 1,200.00]	19.8	480.00 [160.00, 1,200.00]	400.00 [140.00, 900.00]	480.00 [160.00, 1,290.00]	18
**MET minutes per week for vigorous activity (median [IQR])**	240.00 [0.00, 960.00]	0.00 [0.00, 820.00]	240.00 [0.00, 960.00]	19.8	240.00 [0.00, 960.00]	240.00 [0.00, 540.00]	240.00 [0.00, 960.00]	18
**Total MET minutes per week (median [IQR])**	1,773.00 [810.00, 3,452.50]	1,367.50 [478.50, 3,834.00]	1,776.50 [810.00, 3,450.00]	19.8	1,895.00 [924.00, 3,626.00]	1,253.00 [711.50, 3,606.00]	1,942.50 [925.50, 3,622.50]	18
**Family heart disease (%)**	4,458 (44.7)	34 (39.1)	4,424 (44.8)	0	389 (48.6)	14 (43.8)	375 (48.8)	0
CARDIAC DISEASES/OUTCOMES								
**Cardiac problem (%)**	41 (0.4)	2 (2.3)	39 (0.4)	0	4 (0.5)	0 (0.0)	4 (0.5)	0
**Heart failure (%)**	182 (1.8)	74 (85.1)	108 (1.1)	0	25 (3.1)	13 (40.6)	12 (1.6)	0
**Cardiomyopathy (%)**	37 (0.4)	26 (29.9)	11 (0.1)	0	25 (3.1)	20 (62.5)	5 (0.7)	0
**Dilated cardiomyopathy (%)**	14 (0.1)	8 (9.2)	6 (0.1)	0	0 (0.0)	0 (0.0)	0 (0.0)	0
**Hypertrophic cardiomyopathy (%)**	8 (0.1)	7 (8.0)	1 (0.0)	0	19 (2.4)	14 (43.8)	5 (0.7)	0
**Ventricular arrhythmias (%)**	33 (0.3)	3 (3.4)	30 (0.3)	0	8 (1.0)	4 (12.5)	4 (0.5)	0
**Atrial arrhythmias (%)**	191 (1.9)	19 (21.8)	172 (1.7)	0	25 (3.1)	6 (18.8)	19 (2.5)	0
**Heart arrhythmia (%)**	54 (0.5)	2 (2.3)	52 (0.5)	0	52 (6.5)	0 (0.0)	52 (6.8)	0
**Chronic ischemic heart disease (%)**	725 (7.3)	0 (0.0)	725 (7.3)	0	22 (2.7)	2 (6.2)	20 (2.6)	0
**Acute myocardial infarction (%)**	298 (3.0)	1 (1.1)	297 (3.0)	0	4 (0.5)	0 (0.0)	4 (0.5)	0
**Cardiac arrest (%)**	34 (0.3)	1 (1.1)	33 (0.3)	0	15 (1.9)	5 (15.6)	10 (1.3)	0
**Angina pectoris (%)**	312 (3.1)	2 (2.3)	310 (3.1)	0	29 (3.6)	8 (25.0)	21 (2.7)	0
**Conduction disorders (%)**	151 (1.5)	10 (11.5)	141 (1.4)	0	3 (0.4)	0 (0.0)	3 (0.4)	0
**Valvular disease (%)**	241 (2.4)	23 (26.4)	218 (2.2)	0	36 (4.5)	3 (9.4)	33 (4.3)	0
**Congenital heart disease (%)**	28 (0.3)	3 (3.4)	25 (0.3)	0	3 (0.4)	0 (0.0)	3 (0.4)	0
**Pulmonary obstructive disease (%)**	494 (5.0)	24 (27.6)	470 (4.8)	0	32 (4.0)	0 (0.0)	32 (4.2)	0
**Cardiovascular death (%)**	181 (1.8)	13 (14.9)	168 (1.7)	0	10 (1.2)	3 (9.4)	7 (0.9)	0
**All-cause mortality (%)**	513 (5.1)	27 (31.0)	486 (4.9)	0	45 (5.6)	5 (15.6)	40 (5.2)	0
ECG MEASUREMENTS								
**n (%)**	1,062 (10.6)	4 (4.6)	1,058 (10.7)		85 (10.6)	5 (15.6)	80 (10.4)	0
**P duration (median [IQR])**	100.00 [90.00, 108.00]	90.00 [82.00, 111.00]	100.00 [90.00, 108.00]	89.8	100.00 [90.00, 106.00]	100.00 [86.00, 102.00]	100.00 [90.50, 106.00]	89.6
**P axis (median [IQR])**	55.00 [40.25, 67.00]	47.00 [47.00, 47.00]	55.00 [40.00, 67.00]	93.1	50.00 [36.00, 65.50]	70.00 [66.00, 70.00]	48.00 [35.75, 64.25]	92.1
**PQ interval (median [IQR])**	160.00 [145.50, 178.00]	188.00 [188.00, 188.00]	160.00 [145.00, 178.00]	93.1	164.00 [145.00, 174.00]	190.00 [186.00, 199.00]	163.00 [143.50, 172.00]	92.1
**QRS duration (median [IQR])**	86.00 [80.00, 94.00]	93.00 [89.50, 97.00]	86.00 [80.00, 94.00]	89.4	86.00 [80.00, 92.00]	100.00 [82.00, 104.00]	85.00 [80.00, 92.00]	89.4
**R axis (median [IQR])**	34.00 [7.00, 58.00]	-48.00 [-48.00, -48.00]	34.00 [7.50, 58.00]	92.9	38.50 [9.75, 55.00]	2.00 [-17.00, 24.00]	39.00 [13.00, 55.00]	92
**QTC interval (median [IQR])**	417.00 [402.00, 433.00]	511.00 [511.00, 511.00]	417.00 [402.00, 432.50]	92.9	415.00 [401.75, 429.25]	435.00 [428.50, 456.50]	414.00 [401.00, 429.00]	92
**T axis (median [IQR])**	40.00 [23.00, 55.25]	92.00 [92.00, 92.00]	40.00 [23.00, 55.00]	92.9	45.00 [30.75, 61.25]	49.00 [43.00, 96.00]	45.00 [30.00, 61.00]	92
CMR MEASUREMENTS								
**n (%)**	990 (9.9)	4 (4.6)	986 (10.0)		84 (10.5)	4 (12.5)	80 (10.4)	0
**RVEDVi (median [IQR])**	80.18 [70.62, 90.27]	79.28 [79.28, 79.28]	80.19 [70.61, 90.27]	91	77.46 [68.84, 93.43]	79.56 [77.34, 90.20]	77.39 [67.84, 93.43]	89.9
**RVESVi (median [IQR])**	32.89 [27.38, 39.68]	20.93 [20.93, 20.93]	32.90 [27.42, 39.70]	91	31.40 [26.87, 37.04]	34.80 [30.85, 43.04]	31.35 [26.22, 36.21]	89.9
**RVSVi (median [IQR])**	46.55 [40.92, 52.84]	58.35 [58.35, 58.35]	46.53 [40.91, 52.81]	91	47.18 [41.46, 55.77]	47.41 [45.02, 51.27]	47.18 [41.46, 55.77]	89.9
**RVEF (median [IQR])**	58.38 [54.19, 62.76]	73.60 [73.60, 73.60]	58.37 [54.19, 62.74]	91	59.99 [56.00, 63.95]	56.21 [51.67, 60.89]	59.99 [56.46, 64.04]	90
**RVPER (median [IQR])**	388.72 [316.56, 465.82]	446.55 [446.55, 446.55]	388.66 [316.54, 465.97]	91	398.00 [333.00, 478.60]	391.95 [296.40, 448.95]	398.00 [333.00, 478.60]	89.9
**RVPFR (median [IQR])**	300.61 [245.17, 364.00]	373.24 [373.24, 373.24]	300.34 [245.12, 363.44]	91	294.17 [243.48, 338.50]	363.15 [334.20, 386.48]	278.70 [243.13, 334.41]	89.9
**RVPAFR (median [IQR])**	282.95 [222.68, 360.32]	547.72 [547.72, 547.72]	282.86 [222.56, 360.07]	91	302.92 [251.30, 369.30]	263.65 [225.50, 297.22]	307.70 [254.50, 378.30]	89.9
**LVEDVi (median [IQR])**	74.33 [66.34, 83.11]	64.68 [59.39, 69.33]	74.37 [66.38, 83.15]	91.9	78.88 [67.38, 87.06]	85.13 [83.78, 87.08]	77.40 [66.41, 87.06]	90.9
**LVESVi (median [IQR])**	30.02 [25.12, 35.70]	26.43 [25.24, 28.39]	30.02 [25.13, 35.72]	91.9	31.28 [25.63, 37.04]	43.58 [39.30, 47.19]	31.22 [25.36, 35.84]	90.9
**LVSVi (median [IQR])**	44.03 [39.34, 50.28]	38.25 [31.00, 44.09]	44.05 [39.37, 50.30]	91.9	45.49 [39.66, 51.78]	44.56 [42.26, 45.10]	45.74 [39.66, 52.12]	90.9
**LVEF (median [IQR])**	59.47 [55.29, 63.52]	59.14 [51.51, 63.31]	59.48 [55.29, 63.52]	91.9	58.64 [55.31, 62.35]	51.02 [47.77, 53.27]	58.91 [55.57, 62.38]	90.9
**LVPER (median [IQR])**	373.80 [302.29, 452.71]	284.40 [256.83, 364.56]	373.81 [302.44, 453.27]	91.9	351.29 [253.70, 463.30]	350.15 [311.48, 380.62]	351.29 [253.00, 486.30]	90.9
**LVPFR (median [IQR])**	321.31 [259.23, 385.16]	201.74 [189.85, 229.92]	321.49 [259.95, 385.63]	91.9	336.70 [260.80, 380.00]	315.75 [273.85, 360.95]	336.70 [259.70, 383.50]	90.9
**LVPAFR (median [IQR])**	233.66 [167.35, 306.30]	363.87 [208.97, 466.86]	233.50 [167.46, 305.08]	91.9	254.63 [181.10, 323.00]	213.90 [135.76, 272.58]	254.63 [182.20, 323.97]	90.9
**LVEDMi (median [IQR])**	41.88 [36.52, 48.62]	45.76 [34.02, 48.75]	41.85 [36.55, 48.61]	91.9	45.21 [37.68, 50.86]	49.64 [46.84, 53.17]	44.67 [37.47, 49.86]	90.9
**LVMVR (median [IQR])**	0.56 [0.50, 0.62]	0.70 [0.56, 0.70]	0.56 [0.50, 0.62]	91.9	0.57 [0.50, 0.65]	0.58 [0.55, 0.61]	0.57 [0.50, 0.65]	90.9
**LVEDV/RVEDV (median [IQR])**	0.93 [0.86, 1.03]	0.93 [0.93, 0.93]	0.93 [0.86, 1.03]	92.2	0.95 [0.86, 1.05]	1.07 [0.99, 1.08]	0.95 [0.86, 1.03]	91
**LVESV/RVESV (median [IQR])**	0.91 [0.80, 1.04]	1.15 [1.15, 1.15]	0.91 [0.80, 1.04]	92.2	0.96 [0.86, 1.10]	1.27 [1.14, 1.30]	0.96 [0.86, 1.09]	91
**peakEcc (median [IQR])**	-22.70 [-24.98, -20.43]	-21.60 [-21.61, -21.59]	-22.72 [-24.98, -20.42]	93.7	-22.59 [-24.30, -20.60]	-20.32 [-20.51, -20.27]	-22.78 [-24.41, -20.88]	92.1
**TPKEcc (median [IQR])**	331.31 [309.83, 354.66]	325.97 [321.27, 330.67]	331.31 [309.75, 354.68]	93.7	332.34 [309.34, 354.95]	347.70 [320.45, 364.20]	331.96 [309.94, 354.92]	92.1
**peakEll2Ch (median [IQR])**	-21.19 [-23.34, -18.93]	-23.89 [-23.89, -23.89]	-21.17 [-23.32, -18.93]	93.8	-20.32 [-22.79, -18.60]	-20.63 [-20.98, -20.18]	-20.28 [-22.92, -18.55]	92.1
**TPKEll2Ch (median [IQR])**	349.86 [321.84, 379.10]	388.50 [388.50, 388.50]	349.86 [321.81, 379.08]	93.9	364.20 [334.60, 384.50]	403.20 [386.90, 420.80]	362.52 [333.25, 382.95]	92.1
**peakEll4Ch (median [IQR])**	-23.30 [-25.97, -21.37]	-21.40 [-21.40, -21.40]	-23.30 [-25.99, -21.37]	93.9	-23.56 [-26.34, -21.77]	-20.87 [-22.20, -19.78]	-23.57 [-26.50, -22.22]	93
**TPKEll4Ch (median [IQR])**	357.53 [327.07, 397.64]	360.78 [360.78, 360.78]	357.30 [326.96, 397.81]	93.9	350.13 [323.33, 395.18]	403.25 [378.53, 407.65]	348.81 [323.33, 390.17]	93
**Wall thickness segment 1 (median [IQR])**	7.65 [6.81, 8.50]	7.04 [5.94, 8.14]	7.65 [6.81, 8.49]	93.2	8.15 [7.16, 9.17]	9.35 [9.26, 9.44]	7.93 [6.97, 9.11]	94.1
**Wall thickness segment 2 (median [IQR])**	6.75 [5.75, 7.91]	7.50 [6.70, 8.31]	6.75 [5.74, 7.90]	93.2	7.21 [5.77, 8.72]	9.50 [8.42, 10.57]	7.12 [5.70, 8.57]	94.1
**Wall thickness segment 3 (median [IQR])**	6.05 [5.17, 6.96]	7.60 [6.78, 8.42]	6.05 [5.17, 6.95]	93.2	6.42 [5.21, 7.97]	7.99 [7.50, 8.47]	6.37 [5.20, 7.96]	94.1
**Wall thickness segment 4 (median [IQR])**	6.54 [5.82, 7.22]	8.31 [7.66, 8.97]	6.54 [5.81, 7.21]	93.2	6.54 [6.09, 7.27]	7.25 [7.12, 7.38]	6.49 [6.07, 7.26]	94.1
**Wall thickness segment 5 (median [IQR])**	6.20 [5.62, 6.96]	6.33 [5.27, 7.39]	6.20 [5.62, 6.96]	93.2	6.25 [5.59, 6.76]	6.36 [6.00, 6.72]	6.25 [5.59, 6.75]	94.1
**Wall thickness segment 6 (median [IQR])**	6.55 [5.97, 7.31]	6.61 [5.42, 7.79]	6.55 [5.98, 7.31]	93.2	6.64 [6.16, 7.46]	6.72 [6.45, 6.98]	6.64 [6.15, 7.52]	94.1
**Wall thickness segment 7 (median [IQR])**	5.73 [5.28, 6.31]	5.48 [4.90, 6.07]	5.73 [5.29, 6.31]	93.2	5.75 [5.35, 6.33]	6.03 [5.92, 6.13]	5.74 [5.32, 6.37]	94.1
**Wall thickness segment 8 (median [IQR])**	7.01 [6.28, 7.76]	8.52 [7.77, 9.27]	7.01 [6.28, 7.75]	93.2	7.13 [6.27, 7.59]	8.76 [7.66, 9.86]	7.13 [6.18, 7.54]	94.1
**Wall thickness segment 9 (median [IQR])**	7.38 [6.48, 8.25]	9.47 [8.05, 10.89]	7.38 [6.48, 8.25]	93.2	7.60 [6.77, 8.32]	8.99 [7.76, 10.21]	7.60 [6.89, 8.29]	94.1
**Wall thickness segment 10 (median [IQR])**	6.23 [5.59, 6.96]	7.07 [6.08, 8.06]	6.23 [5.60, 6.96]	93.2	6.47 [5.87, 7.10]	6.48 [6.17, 6.78]	6.47 [5.88, 7.12]	94.1
**Wall thickness segment 11 (median [IQR])**	5.62 [5.09, 6.32]	5.57 [5.19, 5.95]	5.62 [5.10, 6.32]	93.2	5.68 [5.33, 6.54]	5.75 [5.62, 5.88]	5.68 [5.32, 6.69]	94.1
**Wall thickness segment 12 (median [IQR])**	5.60 [5.22, 6.25]	5.56 [5.53, 5.60]	5.60 [5.22, 6.26]	93.2	5.64 [5.25, 6.00]	5.53 [5.32, 5.73]	5.64 [5.32, 6.02]	94.1
**Wall thickness segment 13 (median [IQR])**	5.48 [5.10, 5.91]	8.01 [7.03, 9.00]	5.48 [5.10, 5.91]	93.2	5.59 [5.19, 5.93]	5.21 [4.61, 5.82]	5.59 [5.22, 5.91]	94.1
**Wall thickness segment 14 (median [IQR])**	6.00 [5.36, 6.66]	8.33 [6.87, 9.79]	6.00 [5.36, 6.66]	93.2	5.94 [5.49, 6.61]	6.05 [5.36, 6.74]	5.94 [5.53, 6.55]	94.1
**Wall thickness segment 15 (median [IQR])**	5.09 [4.48, 5.69]	7.21 [5.80, 8.63]	5.09 [4.48, 5.69]	93.2	5.16 [4.60, 5.73]	5.30 [5.05, 5.54]	5.16 [4.58, 5.70]	94.1
**Wall thickness segment 16 (median [IQR])**	5.26 [4.80, 5.72]	8.31 [6.87, 9.75]	5.26 [4.80, 5.72]	93.2	5.23 [5.01, 5.93]	5.32 [4.77, 5.87]	5.23 [5.02, 5.91]	94.1
**Global wall thickness (median [IQR])**	6.30 [5.73, 6.84]	7.33 [7.28, 7.38]	6.30 [5.72, 6.84]	93.2	6.51 [5.85, 7.17]	7.06 [6.95, 7.17]	6.44 [5.79, 7.17]	94.1
**Septal wall thickness (median [IQR])**	6.68 [5.88, 7.35]	8.28 [7.88, 8.69]	6.67 [5.88, 7.34]	93.2	6.84 [6.18, 8.03]	8.26 [7.96, 8.55]	6.74 [6.17, 8.00]	94.1
**Maximum wall thickness (median [IQR])**	8.10 [7.24, 9.02]	10.96 [10.29, 11.64]	8.09 [7.24, 9.01]	93.2	8.54 [7.65, 10.15]	11.54 [11.49, 11.59]	8.47 [7.59, 9.94]	94.1

Abbreviations:
ARVC: arrythmogenic right ventricular cardiomyopathy; BMI: body mass index; CMR: cardiac magnetic resonance imaging; DCM: dilated cardiomyopathy; ECG: Electrocardiography; EDVi: indexed end-diastolic volume; EDMi: indexed end-diastolic mass; EF: ejection fraction; ESVi: indexed end-systolic volume; G+: carriers of likely pathogenic and pathogenic variants associated with one of the cardiomyopathies; HCM: hypertrophic cardiomyopathy; HDL: high-density lipoprotein; IQR: interquartile range; LDL: low-density lipoprotein; LV: left ventricular; MET: metabolic equivalent of task; MVR: mass to volume ratio; PAFR: peak atrial filling rate; peakEcc: peak circumferential strain; peakEll2Ch: longitudinal strain analyzed in 2-chamber view; peakEll4Ch: longitudinal strain analyzed in 4-chamber view; PER: peak ejection rate; PFR: peak filling rate; RV: right ventricular; SVi: indexed stroke volume; TPKEcc: global time to peak circumferential strain; TPKEll2Ch: global time to longitudinal strain analyzed in 2-chamber view; TPKEll4Ch: global time to longitudinal strain analyzed in 4-chamber view.

Supplementary Table IV: Detailed information of all included SNPs

SNP	GRCh37	Gene	rsID	Accession ClinVar	Canonical SPDI	N ARVC	N DCM	N HCM	MAF	Origin	Molecular Consequence	Amino acid change	Nucleotide change
11:47332274:D:25	11:47353825	*MYBPC3*	rs36212066	VCV000177677	NC_000011.10:47332274:GAGAGGGAGGG	NA	NA	303	7.66E-04	VKGL	NA	NA	3628-41_3628-17del
1:201359245:G:A	1:201328373	*TNNT2*	rs121964857	VCV000012411	NC_000001.11:201359244:G:A	NA	NA	242	6.03E-04	VKGL	Missense	Arg278Cys	862C>T
11:47342698:G:A	11:47364249	*MYBPC3*	rs375882485	VCV000042540	NC_000011.10:47342697:G:A	NA	NA	88	2.19E-04	VKGL	Missense	Arg502Trp	1504C>T
12:32802557:C:G	12:32955491	*PKP2*	rs193922674	VCV000006756	NC_000012.12:32802556:C:G	46	NA	NA	1.15E-04	ClinVar	Splice acceptor	NA	2014-1G>C
7:128856810:G:A	7:128496864	*FLNC*	rs778922568	VCV000472173	NC_000007.14:128856809:G:A	NA	**42**	NA	1.05E-04	VKGL	Missense	Gly2484Ser	7450G>A
12:32802499:D:5	12:32955434-32955438	*PKP2*	rs397517021	VCV000689321	NC_000012.12:32802499:GGTGTG:G	40	NA	NA	9.97E-05	ClinVar	Frameshift	His689fs	2066_2070del
11:47350077:C:T	11:47371628	*MYBPC3*	rs397516050	VCV000042752	NC_000011.10:47350076:C:T	NA	NA	34	8.47E-05	VKGL	Missense	Gly148Arg	442G>A
14:23417573:A:G	14:23886782	*MYH7*	rs727503244	VCV000164289	NC_000014.9:23417572:A:G	NA	34	34	8.47E-05	VKGL	Missense	Leu1428Ser	4283T>C
2:219425699:C:A	2:220290421	*DES*	rs121913005	VCV000626715	NC_000002.12:219425698:C:A	NA	**31**	NA	7.73E-05	VKGL	Missense	Thr442Asn	1325C>A
11:47342574:T:A	11:47364125	*MYBPC3*	rs397515916	VCV000042556	NC_000011.10:47342573:T:A	NA	NA	28	6.98E-05	ClinVar	NA	NA	1624+4A>T
12:32878134:C:T	12:33031068	*PKP2*	rs1085307949	VCV000427088	NC_000012.12:32878133:C:T	28	NA	NA	6.98E-05	VKGL	Missense	Ser249Asn	746G>A
11:47343314:C:T	11:47364865	*MYBPC3*	NA	VCV000188544	NC_000011.10:47343313:C:T	NA	NA	26	6.48E-05	ClinVar	NA	NA	1224-52G>A
14:23414007:C:T	14:23883216	*MYH7*	rs753392652	VCV000378215	NC_000014.9:23414006:C:T	NA	NA	24	6.23E-05	ClinVar	Synonymous	Ala1885=	5655G>A
14:23422292:G:A	14:23891501	*MYH7*	rs45611033	VCV000177753	NC_000014.9:23422291:G:A	NA	24	24	5.98E-05	ClinVar	Missense	Arg1045Cys	3133C>T
17:41757751:C:A	17:39914003	*JUP*	r200327969	VCV000180376	NC_000017.11:41757750:C:A	24	NA	NA	5.98E-05	VKGL	Missense	Val603Leu	1807G>T
11:47343117:G:A	11:47364668	*MYBPC3*	rs368770848	VCV000042516	NC_000011.10:47343116:G:A	NA	NA	23	5.73E-05	VKGL	Missense	Arg419Cys	1255C>T
11:19188286:A:G	11:19209833	*CSRP3*	rs104894205	VCV000008778	NC_000011.10:19188285:A:G	NA	NA	21	5.23E-05	VKGL	Missense	Leu44Pro	131T>C
11:47342578:C:G	11:47364129	*MYBPC3*	rs121909374	VCV000008608	NC_000011.10:47342577:C:G	NA	NA	21	5.23E-05	ClinVar	Missense	Glu542Gln	1624G>C
11:47346276:C:T	11:47367827	*MYBPC3*	rs397515881	VCV000042499	NC_000011.10:47346275:C:T	NA	NA	20	4.98E-05	VKGL	Missense	Gly341Ser	1021G>A
2:178528273:C:T	2:179393000	*TTN*	rs112188483	VCV000196723	NC_000002.12:178528272:C:T	NA	20	NA	4.98E-05	ClinVar	Splice donor	NA	107377+1G>A
14:23428957:C:T	14:23898166	*MYH7*	rs397516106	VCV000177921	NC_000014.9:23428956:C:T	NA	NA	19	4.74E-05	VKGL	Missense	Asp469Asn	1405G>A
3:38550326:C:T	3:38591817	*SCN5A*	rs762981322	VCV000201549	NC_000003.12:38550325:C:T	NA	19	NA	4.74E-05	VKGL	Missense	Val2015Met	6043G>A
12:32896580:D:4	12:33049514	*PKP2*	rs397516997	VCV000045028	NC_000012.12:32896580:CTGTCTG:CTG	18	NA	NA	4.49E-05	VKGL	Frameshift	Thr50fs	148_151del
11:47351507:T:C	11:47373058	*MYBPC3*	rs376395543	VCV000042644	NC_000011.10:47351506:T:C	NA	NA	17	4.24E-05	ClinVar	Splice acceptor	NA	26-2A>G
2:178579702:G:A	2:179444429	*TTN*	rs574660186	VCV000180573	NC_000002.12:178579701:G:A	NA	17	NA	4.24E-05	ClinVar	Nonsense	Arg22499Ter	67495C>T
11:47337534:C:T	11:47359085	*MYBPC3*	rs2856655	VCV000008617	NC_000011.10:47337533:C:T	NA	NA	16	3.99E-05	ClinVar	Missense	Arg820Gln	2459G>A
18:31089461:C:T	18:28669424	*DSC2*	rs758527425	VCV000372720	NC_000018.10:31089460:C:T	15	NA	NA	3.74E-05	VKGL	Missense	Arg203His	608G>A
2:178613938:C:T	2:179478665	*TTN*	rs869312070	VCV000223309	NC_000002.12:178613937:C:T	NA	15	NA	3.74E-05	ClinVar	Splice acceptor	NA	22151-1G>A
14:23427746:T:C	14:23896955	*MYH7*	rs727504238	VCV000177625	NC_000014.9:23427745:T:C	NA	14	14	3.49E-05	ClinVar	Missense	His576Arg	1727A>G
1:156135268:C:T	1:156105059	*LMNA*	rs59885338	VCV000014498	NC_000001.11:156135267:C:T	NA	13	NA	3.24E-05	VKGL	Missense	Arg298Cys	892C>T
11:47337729:I:1	11:47359280	*MYBPC3*	rs397515963	VCV000042619	NC_000011.10:47337729:C:CC	NA	NA	13	3.24E-05	ClinVar	Frameshift	Trp792fs	2373dup
14:23425316:C:T	14:23894525	*MYH7*	rs3218716	VCV000042901	NC_000014.9:23425315:C:T	NA	13	13	3.24E-05	ClinVar	Missense	Ala797Thr	2389G>A
18:31087815:T:C	18:28667778	*DSC2*	rs397514042	VCV000016850	NC_000018.10:31087814:T:C	13	NA	NA	3.24E-05	VKGL	Splice acceptor	NA	631-2A>G
19:55154821:G:A	19:55666189	*TNNI3*	rs730881068	VCV000181575	NC_000019.10:55154820:G:A	NA	13	13	3.24E-05	VKGL	Nonsense	Arg98Ter	292C>T
19:55154094:C:T	19:55665462	*TNNI3*	rs397516354	VCV000043389	NC_000019.10:55154093:C:T	NA	12	12	2.99E-05	ClinVar	Missense	Arg162Gln	485G>A
3:38562422:C:A	3:38603913	*SCN5A*	rs199473220	VCV000067838	NC_000003.12:38562421:C:A	NA	12	NA	2.99E-05	ClinVar	Missense	Gly1318Val	3953G>T
10:119669881:C:T	10:121429393	*BAG3*	rs387906874	VCV000030396	NC_000010.11:119669880:C:T	NA	11	NA	2.74E-05	VKGL	Missense	Arg71Trp	211C>T
2:178777234:D:5	2:179641961	*TTN*	rs756433029	VCV000202501	NC_000002.12:178777234:TTTCATTTCA:TT	NA	11	NA	2.74E-05	VKGL	Frameshift	Met1575fs	4724_4728del
1:156134823:C:T	1:156104614	*LMNA*	rs370134870	VCV000264626	NC_000001.11:156134822:C:T	NA	10	NA	2.49E-05	VKGL	Missense	Arg220Cys	658C>T
11:47342718:C:T	11:47364269	*MYBPC3*	rs200411226	VCV000164113	NC_000011.10:47342717:C:T	NA	NA	10	2.49E-05	ClinVar	Missense	Arg495Gln	1484G>A
11:47348541:C:G	11:47370092	*MYBPC3*	rs397516068	VCV000042784	NC_000011.10:47348540:C:G	NA	NA	10	2.49E-05	ClinVar	Missense	Val219Leu	655G>C
2:178620285:G:T	2:179485012	*TTN*	rs368200299	VCV000223308	NC_000002.12:178620284:G:T	NA	10	NA	2.49E-05	ClinVar	Nonsense	Cys15412Ter	46236C>A
3:38613773:G:A	3:38655264	*SCN5A*	rs199473072	VCV000068032	NC_000003.12:38613772:G:A	NA	10	NA	2.49E-05	ClinVar	Missense	Arg225Trp	673C>T
6:7585760:C:G	6:7585993	*DSP*	NA	VCV000924608	NC_000006.12:7585759:C:G	NA	10	NA	2.49E-05	VKGL	Missense	Ser2833Cys	8498C>G
11:47342854:G:A	11:47364405	*MYBPC3*	rs730880540	VCV000180935	NC_000011.10:47342853:G:A	NA	NA	9	2.24E-05	VKGL	Missense	Ser478Leu	1433C>T
11:47348424:C:T	11:47369975	*MYBPC3*	rs397516074	VCV000042792	NC_000011.10:47348423:C:T	NA	NA	9	2.24E-05	ClinVar	Missense	Glu258Lys	772G>A
12:110913140:G:A	12:111350944	*MYL2*	rs397516404	VCV000043471	NC_000012.12:110913139:G:A	NA	NA	9	2.24E-05	VKGL	Missense	Arg120Trp	358C>T
19:55154095:G:A	19:55665463	*TNNI3*	rs368861241	VCV000161396	NC_000019.10:55154094:G:A	NA	NA	9	2.24E-05	ClinVar	Missense	Arg162Trp	484C>T
2:178534401:A:G	2:179399128	*TTN*	rs375159973	VCV000405075	NC_000002.12:178534400:A:G	NA	9	NA	2.24E-05	VKGL	Missense	Trp34072Arg	102214T>C
2:178579850:T:G	2:179444577	*TTN*	rs753948675	VCV000242425	NC_000002.12:178579849:T:G	NA	9	NA	2.24E-05	ClinVar	Splice acceptor	NA	67349-2A>C
1:156135956:G:A	1:156105747	*LMNA*	rs59301204	VCV000048098	NC_000001.11:156135955:G:A	NA	8	NA	1.99E-05	VKGL	Missense	Arg331Gln	992G>A
1:201364327:G:A	1:201333455	*TNNT2*	rs483352832	VCV000132943	NC_000001.11:201364326:G:A	NA	8	7	1.99E-05	VKGL	Missense	Arg154Trp	460C>T
1:201365620:D:2	1:201334748	*TNNT2*	NA	VCV000925600	NC_000001.11:201365620:CTCTCTCTC:CTCT	NA	8	8	1.99E-05	VKGL	Frameshift	Arg94fs	282_283del
11:47342719:G:C	11:47364270	*MYBPC3*	rs397515905	VCV000042537	NC_000011.10:47342718:G:C	NA	NA	8	1.99E-05	ClinVar	Missense	Arg495Gly	1483C>G
12:110919133:C:T	12:111356937	*MYL2*	rs104894368	VCV000014065	NC_000012.12:110919132:C:T	NA	NA	8	1.99E-05	ClinVar	Missense	Glu22Lys	64G>A
12:32878545:T:A	12:33031479	*PKP2*	rs786204389	VCV000188654	NC_000012.12:32878544:T:A	8	NA	NA	1.99E-05	ClinVar	Splice acceptor	NA	337-2A>T
2:178531668:G:A	2:179396395	*TTN*	rs991187915	VCV000667024	NC_000002.12:178531667:G:A	NA	8	NA	1.99E-05	ClinVar	Nonsense	Gln34983Ter	104947C>T
3:38551513:G:A	3:38593004	*SCN5A*	rs199473282	VCV000067932	NC_000003.12:38551512:G:A	NA	8	NA	1.99E-05	ClinVar	Missense	Thr1619Met	4856C>T
3:52453993:G:A	3:52488009	*TNNC1*	rs267607125	VCV000012443	NC_000003.12:52453992:G:A	NA	7	7	1.75E-05	ClinVar	Missense	Ala8Val	23C>T

SNP	GRCh37	Gene	rsID	Accession ClinVar	Canonical SPDI	N ARVC	N DCM	N HCM	MAF	Origin	Molecular Consequence	Amino acid change	Nucleotide change
11:47346379:C:T	11:47367930	*MYBPC3*	rs397516083	VCV000042807	NC_000011.10:47346378:C:T	NA	NA	7	1.74E-05	ClinVar	NA	NA	927-9G>A
2:178562716:G:A	2:179427443	*TTN*	NA	VCV000864799	NC_000002.12:178562715:G:A	NA	7	NA	1.74E-05	ClinVar	Nonsense	Arg27806Ter	83416C>T
1:236727715:C:T	1:236891015	*ACTN2*	rs1253211384	VCV000660714	NC_000001.11:236727714:C:T	NA	**6**	NA	1.50E-05	VKGL	Nonsense	Arg192Ter	574C>T
11:47339792:T:C	11:47361343	*MYBPC3*	rs397515937	VCV000042585	NC_000011.10:47339791:T:C	NA	NA	6	1.50E-05	ClinVar	Splice acceptor	NA	1928-2A>G
12:32796108:C:T	12:32949042	*PKP2*	rs111517471	VCV000006757	NC_000012.12:32796107:C:T	6	NA	NA	1.50E-05	ClinVar	Splice donor	NA	2357+1G>A
12:32822616:I:1	12:32975550-32975551	*PKP2*	rs397517010	VCV000045047	NC_000012.12:32822616:A:AA	6	NA	NA	1.50E-05	ClinVar	Frameshift	Val564fs	1689dup
12:32878981:A:T	12:33031915	*PKP2*	rs763639737	VCV000202026	NC_000012.12:32878980:A:T	6	NA	NA	1.50E-05	ClinVar	Nonsense	Leu92Ter	275T>A
14:23416057:G:A	14:23885266	*MYH7*	rs397516232	VCV000043043	NC_000014.9:23416056:G:A	NA	NA	6	1.50E-05	VKGL	Missense	Arg1634Cys	4900C>T
14:23424840:G:A	14:23894049	*MYH7*	rs138049878	VCV000161326	NC_000014.9:23424839:G:A	NA	6	6	1.50E-05	ClinVar	Missense	Arg870Cys	2608C>T
14:23428631:C:T	14:23897840	*MYH7*	rs121913651	VCV000014119	NC_000014.9:23428630:C:T	NA	NA	6	1.50E-05	VKGL	Missense	Glu483Lys	1447G>A
18:31070724:A:G	18:28650690	*DSC2*	rs1064793731	VCV000419220	NC_000018.10:31070723:A:G	6	NA	NA	1.50E-05	ClinVar	Splice donor	NA	2250+2T>C
2:178582209:C:G	2:179446936	*TTN*	rs1553627403	VCV000466651	NC_000002.12:178582208:C:G	NA	6	NA	1.50E-05	ClinVar	Splice acceptor	NA	66161-1G>C
2:178684990:C:T	2:179549717	*TTN*	rs371725574	VCV000194146	NC_000002.12:178684989:C:T	NA	6	NA	1.50E-05	VKGL	Splice acceptor	NA	32471-1G>A
20:44160293:G:A	20:42788933	*JPH2*	rs387906898	VCV000030457	NC_000020.11:44160292:G:A	NA	NA	6	1.50E-05	ClinVar	Missense	Ser165Phe	494C>T
11:19188281:T:G	11:19209828	*CSRP3*	rs137852765	VCV000008781	NC_000011.10:19188280:T:G	NA	NA	5	1.25E-05	VKGL	Missense	Ser46Arg	136A>C
11:47333189:C:G	11:47354740	*MYBPC3*	rs373746463	VCV000042707	NC_000011.10:47333188:C:G	NA	NA	5	1.25E-05	ClinVar	NA	NA	3330+5G>T
11:47341219:C:T	11:47362770	*MYBPC3*	rs368482358	VCV000180951	NC_000011.10:47341218:C:T	NA	NA	5	1.25E-05	VKGL	Missense	Val606Ile	1816G>A
11:47341991:C:T	11:47363542	*MYBPC3*	rs727503195	VCV000164098	NC_000011.10:47341990:C:T	NA	NA	5	1.25E-05	VKGL	Missense	Arg597Gln	1790G>A
11:47342611:C:G	11:47364162	*MYBPC3*	rs397515912	VCV000042550	NC_000011.10:47342610:C:G	NA	NA	5	1.25E-05	ClinVar	Missense	Gly531Arg	1591G>C
14:23417598:G:A	14:23886807	*MYH7*	rs145213771	VCV000043003	NC_000014.9:23417597:G:A	NA	5	5	1.25E-05	ClinVar	Missense	Arg1420Trp	4258C>T
14:23418304:G:A	14:23887513	*MYH7*	rs45451303	VCV000178082	NC_000014.9:23418303:G:A	NA	5	5	1.25E-05	VKGL	Missense	Arg1359Cys	4075C>T
14:23424112:T:C	14:23893321	*MYH7*	rs267606908	VCV000014125	NC_000014.9:23424111:T:C	NA	5	5	1.25E-05	ClinVar	Missense	Asp906Gly	2717A>G
14:23429037:C:T	14:23898246	*MYH7*	rs730880870	VCV000181342	NC_000014.9:23429036:C:T	NA	5	5	1.25E-05	VKGL	Missense	Arg442His	1325G>A
19:55156638:I:1	19:55668006	*TNNI3*	rs772607683	VCV000419596	NC_000019.10:55156638:TTTTTT:TTTTTTT	NA	5	5	1.25E-05	VKGL	Frameshift	Ser39fs	114dup
2:178560865:G:A	2:179425592	*TTN*	NA	VCV000853671	NC_000002.12:178560864:G:A	NA	5	NA	1.25E-05	ClinVar	Nonsense	Arg28423Ter	85267C>T
2:178574530:G:A	2:179439257	*TTN*	rs397517689	VCV000047301	NC_000002.12:178574529:G:A	NA	5	NA	1.25E-05	ClinVar	Nonsense	Arg23868Ter	71602C>T
2:178589849:G:A	2:179454576	*TTN*	rs72646846	VCV000047175	NC_000002.12:178589848:G:A	NA	5	NA	1.25E-05	ClinVar	Nonsense	Arg20626Ter	61876C>T
2:178767782:G:A	2:179632509	*TTN*	rs146572907	VCV000282852	NC_000002.12:178767781:G:A	NA	5	NA	1.25E-05	VKGL	Nonsense	Arg3150Ter	9448C>T
2:219423821:G:A	2:220288543	*DES*	rs112224037	VCV000639517	NC_000002.12:219423820:G:A	5	5	NA	1.25E-05	ClinVar	Splice donor	NA	1288+1G>A
6:7569211:G:A	6:7569444	*DSP*	NA	VCV000956247	NC_000006.12:7569210:G:A	5	5	NA	1.25E-05	VKGL	Missense	Cys482Tyr	1445G>A
6:7579922:I:7	6:7580155-7580156	*DSP*	rs1554108152	VCV000199923	NC_000006.12:7579922:GAAAATCGA:GAA	5	NA	NA	1.25E-05	ClinVar	Frameshift	Asp1248fs	3735_3741dup
1:156134454:C:T	1:156104245	*LMNA*	rs267607626	VCV000066906	NC_000001.11:156134453:C:T	NA	4	NA	9.97E-06	VKGL	Missense	Arg189Trp	565C>T
1:156136311:C:T	1:156106102	*LMNA*	rs1064793731	VCV000242002	NC_000001.11:156136310:C:T	NA	4	NA	9.97E-06	VKGL	Missense	Arg419Cys	1255C>T
1:201361317:A:C	1:201330445	*TNNT2*	rs730881110	VCV000181645	NC_000001.11:201361316:A:C	NA	4	4	9.97E-06	VKGL	Missense	Phe258Val	772T>G
10:110812459:C:T	10:112572217	*RBM20*	rs794729150	VCV000202065	NC_000010.11:110812458:C:T	NA	4	NA	9.97E-06	VKGL	Nonsense	Arg688Ter	2062C>T
11:47337544:G:A	11:47359095	*MYBPC3*	rs727503188	VCV000164078	NC_000011.10:47337543:G:A	NA	NA	4	9.97E-06	ClinVar	Missense	Arg817Trp	2449C>T
11:47342750:C:T	11:47364301	*MYBPC3*	rs375347534	VCV000042533	NC_000011.10:47342749:C:T	NA	NA	4	9.97E-06	VKGL	Na	NA	1458-6G>A
12:110911176:C:G	12:111348980	*MYL2*	rs199474813	VCV000031768	NC_000012.12:110911175:C:G	NA	NA	4	9.97E-06	ClinVar	Splice acceptor	NA	403-1G>C
12:32878217:G:T	12:33031151	*PKP2*	rs767987619	VCV000201976	NC_000012.12:32878216:G:T	4	NA	NA	9.97E-06	ClinVar	Nonsense	Tyr221Ter	663C>A
14:23415651:C:T	14:23884860	*MYH7*	rs193922390	VCV000036642	NC_000014.9:23415650:C:T	NA	4	4	9.97E-06	ClinVar	Missense	Arg1712Gln	5135G>A
14:23418348:C:T	14:23887557	*MYH7*	rs797045097	VCV000208597	NC_000014.9:23418347:C:T	NA	NA	4	9.97E-06	VKGL	Missense	Arg1344Gln	4031G>A
14:23424876:G:A	14:23894085	*MYH7*	rs2754158	VCV000164324	NC_000014.9:23424875:G:A	NA	4	4	9.97E-06	ClinVar	Missense	Arg858Cys	2572C>T
18:31086694:G:A	18:28666657	*DSC2*	rs397517404	VCV000222557	NC_000018.10:31086693:G:A	4	NA	NA	9.97E-06	VKGL	Missense	Thr275Met	824C>T
18:31498254:G:A	18:29078217	*DSG2*	rs1021457619	VCV000657863	NC_000018.10:31498253:G:A	4	NA	NA	9.97E-06	ClinVar	Missense	Met1Ile	3G>A
19:55151881:C:T	19:55663249	*TNNI3*	rs104894727	VCV000012422	NC_000019.10:55151880:C:T	NA	NA	4	9.97E-06	ClinVar	Missense	Asp196Asn	586G>A
2:178539559:G:A	2:179404286	*TTN*	rs869312085	VCV000223329	NC_000002.12:178539558:G:A	NA	4	NA	9.97E-06	ClinVar	Nonsense	Arg32836Ter	98506C>T
2:178584726:G:A	2:179449453	*TTN*	rs1432889079	VCV000466649	NC_000002.12:178584725:G:A	NA	4	NA	9.97E-06	ClinVar	Nonsense	Arg21639Ter	64915C>T
2:178590170:G:A	2:179454897	*TTN*	NA	VCV000202397	NC_000002.12:178590169:G:A	NA	4	NA	9.97E-06	ClinVar	Nonsense	Arg20519Ter	61555C>T
2:219418784:G:T	2:220283506	*DES*	rs62636490	VCV000804737	NC_000002.12:219418783:G:T	4	4	NA	9.97E-06	ClinVar	Nonsense	Glu108Ter	322G>T
3:38597737:C:T	3:38639228	*SCN5A*	rs199473153	VCV000067723	NC_000003.12:38597736:C:T	NA	4	NA	9.97E-06	ClinVar	Missense	Gly752Arg	2254G>A
3:38603999:G:A	3:38645490	*SCN5A*	rs1417036453	VCV000517279	NC_000003.12:38603998:G:A	NA	4	NA	9.97E-06	ClinVar	Nonsense	Arg535Ter	1603C>T
6:118558947:G:A	6:118880110	*PLN*	rs754782171	VCV000202037	NC_000006.12:118558946:G:A	NA	4	NA	9.97E-06	VKGL	Missense	Arg9His	26G>A
6:7565521:G:A	6:7565754	*DSP*	rs727504443	VCV000178282	NC_000006.12:7565520:G:A	4	4	NA	9.97E-06	ClinVar	Splice donor	NA	939+1G>A
6:7583758:C:T	6:7583991	*DSP*	rs141026028	VCV000199903	NC_000006.12:7583757:C:T	4	4	NA	9.97E-06	ClinVar	Nonsense	Arg2166Ter	6496C>T
6:7585028:D:4	6:7585261	*DSP*	NA	VCV000923199	NC_000006.12:7585028:AGTAAGTAAG:AG	4	NA	NA	9.97E-06	VKGL	Frameshift	Ser2591fs	7773_7776del
12:32843181:C:T	12:32996115	*PKP2*	rs1332615728	VCV000640418	NC_000012.12:32843180:C:T	3	NA	NA	7.50E-06	ClinVar	Splice donor	NA	1379-1976G>A
1:201359636:C:T	1:201328764	*TNNT2*	rs121964861	VCV000012417	NC_000001.11:201359635:C:T	NA	3	NA	7.48E-06	ClinVar	Missense	Asp280Asn	838G>A
1:201361970:A:G	1:201331098	*TNNT2*	rs863225120	VCV000217496	NC_000001.11:201361969:A:G	NA	NA	3	7.48E-06	ClinVar	Missense	Ile221Thr	662T>C
11:47332075:G:A	11:47353626	*MYBPC3*	rs397516042	VCV000042744	NC_000011.10:47332074:G:A	NA	NA	3	7.48E-06	ClinVar	Nonsense	Arg1271Ter	3811C>T
11:47341204:C:T	11:47362755	*MYBPC3*	rs397515937	VCV000180955	NC_000011.10:47341203:C:T	NA	NA	3	7.48E-06	VKGL	Missense	Glu611Lys	1831G>A

SNP	GRCh37	Gene	rsID	Accession ClinVar	Canonical SPDI	N ARVC	N DCM	N HCM	MAF	Origin	Molecular Consequence	Amino acid change	Nucleotide change
11:47342611:C:T	11:47364162	*MYBPC3*	rs397515912	VCV000164109	NC_000011.10:47342610:C:T	NA	NA	3	7.48E-06	VKGL	Missense	Gly531Arg	1591G>A
11:47348486:T:G	11:47370037	*MYBPC3*	rs397516070	VCV000042787	NC_000011.10:47348485:T:G	NA	NA	3	7.48E-06	ClinVar	Missense	Tyr237Ser	710A>C
12:32850907:G:A	12:33003841	*PKP2*	rs372827156	VCV000045016	NC_000012.12:32850906:G:A	3	NA	NA	7.48E-06	ClinVar	Nonsense	Arg413Ter	1237C>T
14:23424817:C:A	14:23894026	*MYH7*	rs1060505018	VCV000417718	NC_000014.9:23424816:C:A	NA	NA	3	7.48E-06	ClinVar	Missense	Met877Ile	2631G>T
14:23424839:C:T	14:23894048	*MYH7*	rs36211715	VCV000014120	NC_000014.9:23424838:C:T	NA	3	3	7.48E-06	ClinVar	Missense	Arg870His	2609G>A
14:23424854:T:A	14:23894063	*MYH7*	rs758891557	VCV000454358	NC_000014.9:23424853:T:A	NA	NA	3	7.48E-06	ClinVar	Missense	Lys865Met	2594A>T
14:23425814:G:A	14:23895023	*MYH7*	rs121913630	VCV000014095	NC_000014.9:23425813:G:A	NA	3	3	7.48E-06	ClinVar	Missense	Arg723Cys	2167C>T
14:23427723:C:T	14:23896932	*MYH7*	rs121913626	VCV000042862	NC_000014.9:23427722:C:T	NA	3	3	7.48E-06	ClinVar	Missense	Gly584Ser	1750G>A
14:23429038:G:A	14:23898247	*MYH7*	rs148808089	VCV000177897	NC_000014.9:23429037:G:A	NA	3	3	7.48E-06	ClinVar	Missense	Arg442Cys	1324C>T
14:23429850:C:T	14:23899059	*MYH7*	rs397516088	VCV000042820	NC_000014.9:23429849:C:T	NA	3	3	7.48E-06	ClinVar	Missense	Ala355Thr	1063G>A
18:31074908:C:G	18:28654874	*DSC2*	NA	VCV000860937	NC_000018.10:31074907:C:G	3	NA	NA	7.48E-06	ClinVar	Splice acceptor	NA	1664-1G>C
18:31521213:I:1	18:29101176-29101177	*DSG2*	rs781532110	VCV000280230	NC_000018.10:31521213:TT:TTT	3	NA	NA	7.48E-06	ClinVar	Frameshift	Gly166fs	495dup
18:31521233:G:T	18:29101196	*DSG2*	rs199926617	VCV000577605	NC_000018.10:31521232:G:T	3	NA	NA	7.48E-06	VKGL	Missense	Leu171Phe	513G>T
18:31524549:T:A	18:29104512	*DSG2*	rs869025388	VCV000222562	NC_000018.10:31524548:T:A	3	NA	NA	7.48E-06	ClinVar	Missense	Asp264Glu	792T>A
18:31524744:I:1	18:29104707-29104708	*DSG2*	rs759944835	VCV000639905	NC_000018.10:31524744:A:AA	3	NA	NA	7.48E-06	ClinVar	Frameshift	Thr291fs	871dup
18:31541191:A:G	18:29121154	*DSG2*	rs397514038	VCV000016817	NC_000018.10:31541190:A:G	3	NA	NA	7.48E-06	ClinVar	Splice acceptor	NA	1880-2A>G
2:178546041:G:A	2:179410768	*TTN*	rs753334568	VCV000132137	NC_000002.12:178546040:G:A	NA	3	NA	7.48E-06	ClinVar	Missense	Pro31732Leu	95195C>T
2:178548460:G:A	2:179413187	*TTN*	rs72648250	VCV000223326	NC_000002.12:178548459:G:A	NA	3	NA	7.48E-06	ClinVar	Nonsense	Arg31056Ter	93166C>T
2:178563493:C:A	2:179428220	*TTN*	rs779874042	VCV000202416	NC_000002.12:178563492:C:A	NA	3	NA	7.48E-06	ClinVar	Nonsense	Glu27547Ter	82639G>T
2:178569267:C:T	2:179433994	*TTN*	rs756552975	VCV000379555	NC_000002.12:178569266:C:T	NA	3	NA	7.48E-06	ClinVar	Nonsense	Trp25622Ter	76865G>A
2:178569478:G:A	2:179434205	*TTN*	rs545954490	VCV000404828	NC_000002.12:178569477:G:A	NA	3	NA	7.48E-06	ClinVar	Nonsense	Arg25552Ter	76654C>T
2:178570804:G:A	2:179435531	*TTN*	rs794729382	VCV000202521	NC_000002.12:178570803:G:A	NA	3	NA	7.48E-06	ClinVar	Nonsense	Arg25110Ter	75328C>T
2:178575970:G:A	2:179440697	*TTN*	rs781540455	VCV000202402	NC_000002.12:178575969:G:A	NA	3	NA	7.48E-06	ClinVar	Nonsense	Arg23388Ter	70162C>T
2:178585291:G:A	2:179450018	*TTN*	rs768345594	VCV000223315	NC_000002.12:178585290:G:A	NA	3	NA	7.48E-06	ClinVar	Nonsense	Arg21485Ter	64453C>T
2:178588700:G:A	2:179453427	*TTN*	rs368452607	VCV000202518	NC_000002.12:178588699:G:A	NA	3	NA	7.48E-06	ClinVar	Nonsense	Arg21009Ter	63025C>T
2:178593338:G:A	2:179458065	*TTN*	rs1553649171	VCV000466646	NC_000002.12:178593337:G:A	NA	3	NA	7.48E-06	ClinVar	Nonsense	Arg19624Ter	58870C>T
2:178609756:G:A	2:179474483	*TTN*	NA	VCV001066907	NC_000002.12:178609755:G:A	NA	3	NA	7.48E-06	VKGL	Nonsense	Arg17223Ter	51667C>T
2:178733497:G:A	2:179598224	*TTN*	rs372277017	VCV000130662	NC_000002.12:178733496:G:A	NA	3	NA	7.48E-06	VKGL	Nonsense	Arg5266Ter	15796C>T
2:219421560:G:A	2:220286282	*DES*	rs1262288015	VCV000626714	NC_000002.12:219421559:G:A	NA	**3**	NA	7.48E-06	VKGL	Missense	Arg415Gln	1244G>A
2:219423817:C:T	2:220288539	*DES*	rs150974575	VCV000177872	NC_000002.12:219423816:C:T	3	3	NA	7.48E-06	ClinVar	Nonsense	Arg429Ter	1285C>T
6:118558994:C:T	6:118880157	*PLN*	rs761056344	VCV000202040	NC_000006.12:118558993:C:T	NA	3	NA	7.48E-06	VKGL	Missense	Arg25Cys	73C>T
6:7580388:C:T	6:7580621	*DSP*	rs770873593	VCV000199884	NC_000006.12:7580387:C:T	3	3	NA	7.48E-06	ClinVar	Nonsense	Arg1400Ter	4198C>T
6:7580547:C:T	6:7580780	*DSP*	rs1561698750	VCV000576091	NC_000006.12:7580546:C:T	3	3	NA	7.48E-06	ClinVar	Nonsense	Gln1453Ter	4357C>T
7:128845989:G:C	7:128486043	*FLNC*	rs781135153	VCV000420146	NC_000007.14:128845988:G:C	NA	**3**	NA	7.48E-06	ClinVar	Splice acceptor	NA	3791-1G>C
7:128846444:C:T	7:128486498	*FLNC*	NA	VCV000842060	NC_000007.14:128846443:C:T	NA	**3**	NA	7.48E-06	ClinVar	Nonsense	Arg1370Ter	4108C>T
12:32802499:G:A	12:32955433	*PKP2*	rs121434421	VCV000006755	NC_000012.12:32802498:G:A	2	NA	NA	4.99E-06	ClinVar	Nonsense	Arg691Ter	2071C>T
1:156135913:G:A	1:156105704	*LMNA*	rs56816490	VCV000048093	NC_000001.11:156135912:G:A	NA	2	NA	4.98E-06	ClinVar	Missense	Glu317Lys	949G>A
1:201359217:C:T	1:201328345	*TNNT2*	rs727504247	VCV000177636	NC_000001.11:201359216:C:T	NA	2	2	4.98E-06	ClinVar	Nonsense	Trp297Ter	890G>A
1:201363352:C:A	1:201332480	*TNNT2*	rs730881097	VCV000181612	NC_000001.11:201363351:C:A	NA	2	2	4.98E-06	VKGL	Missense	Ala182Ser	544G>T
1:77926827:G:T	1:78392512	*NEXN*	rs771262904	VCV000599095	NC_000001.11:77926826:G:T	NA	2	NA	4.98E-06	ClinVar	Nonsense	Glu267Ter	799G>T
1:77942736:C:G	1:78408421	*NEXN*	rs794729086	VCV000201935	NC_000001.11:77942735:C:G	NA	2	NA	4.98E-06	ClinVar	Missense	Phe645Leu	1935C>G
10:119670037:C:T	10:121429549	*BAG3*	rs387906875	VCV000030397	NC_000010.11:119670036:C:T	NA	2	NA	4.98E-06	ClinVar	Nonsense	Arg123Ter	367C>T
10:119676479:C:T	10:121435991	*BAG3*	rs869248137	VCV000228322	NC_000010.11:119676478:C:T	NA	2	NA	4.98E-06	ClinVar	Nonsense	Arg309Ter	925C>T
11:47332105:C:T	11:47353656	*MYBPC3*	rs730880141	VCV000180414	NC_000011.10:47332104:C:T	NA	NA	2	4.98E-06	VKGL	Missense	Glu1261Lys	3781G>A
11:47332894:D:3	11:47354445	*MYBPC3*	rs730880674	VCV000181102	NC_000011.10:47332894:AGTAGTAG:AGTA	NA	NA	2	4.98E-06	VKGL	NA	Tyr1136del	3404ACT[1]
11:47333552:C:T	11:47355103	*MYBPC3*	rs587782958	VCV000155808	NC_000011.10:47333551:C:T	NA	NA	2	4.98E-06	ClinVar	NA	NA	3190+5G>A
11:47335041:C:T	11:47356592	*MYBPC3*	rs397515991	VCV000042666	NC_000011.10:47335040:C:T	NA	NA	2	4.98E-06	VKGL	Splice donor	NA	2905+1G>A
11:47335120:G:A	11:47356671	*MYBPC3*	rs387907267	VCV000037039	NC_000011.10:47335119:G:A	NA	NA	2	4.98E-06	ClinVar	Nonsense	Arg943Ter	2827C>T
11:47335165:D:2	11:47356716	*MYBPC3*	rs727504265	VCV000177660	NC_000011.10:47335165:TGTGTG:TGTG	NA	NA	2	4.98E-06	ClinVar	Frameshift	Thr927fs	2780_2781del
11:47336003:D:1	11:47357554	*MYBPC3*	rs397515979	VCV000181083	NC_000011.10:47336003:GGGGGG:GGGG	NA	NA	2	4.98E-06	ClinVar	Frameshift	Ser871fs	2610del
11:47342096:G:A	11:47363647	*MYBPC3*	rs730880694	VCV000656085	NC_000011.10:47342095:G:A	NA	NA	2	4.98E-06	VKGL	Missense	Ala562Val	1685C>T
11:47342697:C:T	11:47364248	*MYBPC3*	rs397515907	VCV000042541	NC_000011.10:47342696:C:T	NA	NA	2	4.98E-06	ClinVar	Missense	Arg502Gln	1505G>A
11:47347856:C:T	11:47369407	*MYBPC3*	rs397516073	VCV000042791	NC_000011.10:47347855:C:T	NA	NA	2	4.98E-06	ClinVar	Splice donor	NA	821+1G>A
11:47352622:C:T	11:47374173	*MYBPC3*	rs113709679	VCV000810740	NC_000011.10:47352621:C:T	NA	NA	2	4.98E-06	ClinVar	Splice donor	NA	25+1G>A
12:32802402:C:T	12:32955336	*PKP2*	rs794729116	VCV000202005	NC_000012.12:32802401:C:T	2	NA	NA	4.98E-06	ClinVar	Splice donor	NA	2167+1G>A
12:32821502:C:A	12:32974436	*PKP2*	rs397517015	VCV000045054	NC_000012.12:32821501:C:A	2	NA	NA	4.98E-06	ClinVar	Nonsense	Glu623Ter	1867G>T
12:32822502:I:19	12:32822502-32822503	*PKP2*	rs1555142971	VCV000523703	NC_000012.12:32822502:AATACTTTTGTTG	2	NA	NA	4.98E-06	ClinVar	Nonsense	Gly602Ter	1785_1803dup
12:32868965:G:A	12:33021899	*PKP2*	rs397516986	VCV000045010	NC_000012.12:32868964:G:A	2	NA	NA	4.98E-06	ClinVar	Nonsense	Gln378Ter	1132C>T
14:23415385:G:A	14:23884594	*MYH7*	rs727505294	VCV000180024	NC_000014.9:23415384:G:A	NA	2	2	4.98E-06	VKGL	Missense	Thr1760Met	5279C>T
14:23417670:G:A	14:23886879	*MYH7*	rs730880793	VCV000181246	NC_000014.9:23417669:G:A	NA	NA	2	4.98E-06	VKGL	Missense	Arg1396Trp	4186C>T

SNP	GRCh37	Gene	rsID	Accession ClinVar	Canonical SPDI	N ARVC	N DCM	N HCM	MAF	Origin	Molecular Consequence	Amino acid change	Nucleotide change
14:23418244:C:T	14:23887453	*MYH7*	rs397516202	VCV000042993	NC_000014.9:23418243:C:T	NA	2	2	4.98E-06	ClinVar	Missense	Ala1379Thr	4135G>A
14:23418313:C:T	14:23887522	*MYH7*	rs727503246	VCV000164294	NC_000014.9:23418312:C:T	NA	NA	2	4.98E-06	ClinVar	Missense	Glu1356Lys	4066G>A
14:23424107:G:C	14:23893316	*MYH7*	rs121913631	VCV000014097	NC_000014.9:23424106:G:C	NA	2	2	4.98E-06	ClinVar	Missense	Leu908Val	2722C>G
14:23424909:T:C	14:23894118	*MYH7*	rs727504310	VCV000177757	NC_000014.9:23424908:T:C	NA	2	2	4.98E-06	ClinVar	Missense	Lys847Glu	2539A>G
14:23426045:C:T	14:23895254	*MYH7*	rs886039030	VCV000264068	NC_000014.9:23426044:C:T	NA	2	2	4.98E-06	ClinVar	Missense	Arg694His	2081G>A
14:23429255:C:T	14:23898464	*MYH7*	rs730880868	VCV000181339	NC_000014.9:23429254:C:T	NA	2	2	4.98E-06	VKGL	Missense	Val411Ile	1231G>A
14:23431602:C:T	14:23900811	*MYH7*	rs397516264	VCV000043100	NC_000014.9:23431601:C:T	NA	2	2	4.98E-06	ClinVar	Missense	Asp239Asn	715G>A
14:23431790:G:A	14:23900999	*MYH7*	rs397516259	VCV000181315	NC_000014.9:23431789:G:A	NA	NA	2	4.98E-06	VKGL	Missense	Arg204Cys	610C>T
15:63060899:G:A	15:63353098	*TPM1*	rs104894503	VCV000012456	NC_000015.10:63060898:G:A	NA	2	2	4.98E-06	ClinVar	Missense	Asp175Asn	523G>A
15:63061723:G:A	15:63353922	*TPM1*	rs199476315	VCV000031882	NC_000015.10:63061722:G:A	NA	NA	2	4.98E-06	ClinVar	Missense	Glu192Lys	574G>A
15:63061751:C:T	15:63353950	*TPM1*	rs730881141	VCV000181668	NC_000015.10:63061750:C:T	NA	NA	2	4.98E-06	VKGL	Missense	Thr201Met	602C>T
18:31519867:G:A	18:29099830	*DSG2*	rs121913006	VCV000016810	NC_000018.10:31519866:G:A	2	NA	NA	4.98E-06	ClinVar	Missense	Arg49His	146G>A
18:31524752:I:1	18:29104715-29104716	*DSG2*	rs1187924885	VCV000691669	NC_000018.10:31524752:AAAA:AAAAA	2	NA	NA	4.98E-06	ClinVar	Frameshift	Val295fs	882dup
18:31545783:T:G	18:29125746	*DSG2*	NA	VCV000943833	NC_000018.10:31545782:T:G	2	NA	NA	4.98E-06	ClinVar	Nonsense	Tyr799Ter	2397T>G
19:55154082:G:A	19:55665450	*TNNI3*	rs727504242	VCV000177630	NC_000019.10:55154081:G:A	NA	NA	2	4.98E-06	ClinVar	Missense	Ser166Phe	497C>T
19:55154145:C:T	19:55665513	*TNNI3*	rs397516349	VCV000043384	NC_000019.10:55154144:C:T	NA	2	2	4.98E-06	ClinVar	Missense	Arg145Gln	434G>A
19:55154146:G:A	19:55665514	*TNNI3*	rs104894724	VCV000012426	NC_000019.10:55154145:G:A	NA	2	2	4.98E-06	ClinVar	Missense	Arg145Trp	433C>T
2:178531788:G:A	2:179396515	*TTN*	NA	VCV000954597	NC_000002.12:178531787:G:A	NA	2	NA	4.98E-06	ClinVar	Nonsense	Arg34943Ter	104827C>T
2:178531962:G:A	2:179396689	*TTN*	rs1057518003	VCV000372824	NC_000002.12:178531961:G:A	NA	2	NA	4.98E-06	ClinVar	Nonsense	Arg34885Ter	104653C>T
2:178532670:G:A	2:179397397	*TTN*	rs995029896	VCV000570433	NC_000002.12:178532669:G:A	NA	2	NA	4.98E-06	ClinVar	Nonsense	Arg34649Ter	103945C>T
2:178532910:T:A	2:179397637	*TTN*	rs1553490574	VCV000499641	NC_000002.12:178532909:T:A	NA	2	NA	4.98E-06	ClinVar	Nonsense	Lys34569Ter	103705A>T
2:178534092:G:A	2:179398819	*TTN*	rs752697861	VCV000464497	NC_000002.12:178534091:G:A	NA	2	NA	4.98E-06	ClinVar	Nonsense	Arg34175Ter	102523C>T
2:178535508:G:A	2:179400235	*TTN*	rs766265889	VCV000625156	NC_000002.12:178535507:G:A	NA	2	NA	4.98E-06	ClinVar	Nonsense	Arg33703Ter	101107C>T
2:178535729:C:T	2:179400456	*TTN*	rs1260821931	VCV000488972	NC_000002.12:178535728:C:T	NA	2	NA	4.98E-06	ClinVar	Nonsense	Trp33629Ter	100886G>A
2:178542263:C:G	2:179406990	*TTN*	rs727505319	VCV000180058	NC_000002.12:178542262:C:G	NA	2	NA	4.98E-06	ClinVar	Splice donor	NA	97492+1G>C
2:178547518:D:5	2:179412245	*TTN*	rs769488730	VCV000202493	NC_000002.12:178547518:TTTAATTT:TTT	NA	2	NA	4.98E-06	VKGL	Frameshift	p.Ile31368fs	94103_94107del
2:178568057:G:T	2:179432784	*TTN*	rs1553597198	VCV000535021	NC_000002.12:178568056:G:T	NA	2	NA	4.98E-06	ClinVar	Missense	Tyr26025Ter	78075C>A
2:178575154:G:A	2:179439881	*TTN*	rs1553612386	VCV000466655	NC_000002.12:178575153:G:A	NA	2	NA	4.98E-06	ClinVar	Nonsense	Arg23660Ter	70978C>T
2:178576691:G:A	2:179441418	*TTN*	rs878854328	VCV000238830	NC_000002.12:178576690:G:A	NA	2	NA	4.98E-06	ClinVar	Nonsense	Arg23185Ter	69553C>T
2:178592916:D:2	2:179457644-179457645	*TTN*	rs752948913	VCV000419310	NC_000002.12:178592916:AG:	NA	2	NA	4.98E-06	ClinVar	Frameshift	Pro19734fs	59201_59202del
2:178609289:G:A	2:179474016	*TTN*	rs926741242	VCV000405082	NC_000002.12:178609288:G:A	NA	2	NA	4.98E-06	ClinVar	Nonsense	Arg17341Ter	52021C>T
2:178612430:G:A	2:179477157	*TTN*	NA	VCV000862652	NC_000002.12:178612429:G:A	NA	2	NA	4.98E-06	ClinVar	Nonsense	Gln16699Ter	50095C>T
2:178617857:G:A	2:179482584	*TTN*	rs751746401	VCV000264517	NC_000002.12:178617856:G:A	NA	2	NA	4.98E-06	ClinVar	Nonsense	Arg15832Ter	47494C>T
2:178740646:G:T	2:179605373	*TTN*	rs370912401	VCV000047827	NC_000002.12:178740645:G:T	NA	2	NA	4.98E-06	VKGL	Nonsense	Ser4196Ter	12587C>A
2:178766474:G:A	2:179631201	*TTN*	rs757836789	VCV000288998	NC_000002.12:178766473:G:A	NA	2	NA	4.98E-06	VKGL	Nonsense	Arg3204Ter	9610C>T
2:219418497:C:T	2:220283219	*DES*	rs267607495	VCV000066412	NC_000002.12:219418496:C:T	2	2	NA	4.98E-06	ClinVar	Missense	Ser12Phe	35C>T
3:38566426:C:T	3:38607917	*SCN5A*	rs137854618	VCV000009401	NC_000003.12:38566425:C:T	NA	2	NA	4.98E-06	ClinVar	Missense	Asp1274Asn	3820G>A
6:7568443:C:T	6:7568676	*DSP*	rs397516915	VCV000044856	NC_000006.12:7568442:C:T	2	2	NA	4.98E-06	ClinVar	Nonsense	Arg425Ter	1273C>T
6:7579527:C:T	6:7579760	*DSP*	rs746877365	VCV000405247	NC_000006.12:7579526:C:T	2	2	NA	4.98E-06	ClinVar	Nonsense	Arg1113Ter	3337C>T
6:7579995:C:T	6:7580228	*DSP*	rs767643821	VCV000199881	NC_000006.12:7579994:C:T	2	2	NA	4.98E-06	ClinVar	Nonsense	Arg1269Ter	3805C>T
6:7582690:C:T	6:7582923	*DSP*	rs397516946	VCV000044928	NC_000006.12:7582689:C:T	2	NA	NA	4.98E-06	ClinVar	Nonsense	Gln1810Ter	5428C>T
7:128844045:C:T	7:128484099	*FLNC*	rs886037830	VCV000267288	NC_000007.14:128844044:C:T	NA	**2**	NA	4.98E-06	ClinVar	Nonsense	Arg991Ter	2971C>T
7:128846136:C:T	7:128486190	*FLNC*	rs766330686	VCV000579589	NC_000007.14:128846135:C:T	NA	**2**	NA	4.98E-06	ClinVar	Nonsense	Arg1313Ter	3937C>T
3:46860702:C:T	3:46902192	*MYL3*	rs199474703	VCV000031777	NC_000003.12:46860701:C:T	NA	NA	1	2.49E-06	ClinVar	Missense	Arg94His	281G>A
6:7584359:G:A	6:7584592	*DSP*	rs387906618	VCV000029672	NC_000006.12:7584358:G:A	NA	1	NA	2.49E-06	ClinVar	Missense	Arg2366His	7097G>A
2:178774206:C:T	2:179638933	*TTN*	NA	VCV000873434	NC_000002.12:178774205:C:T	NA	1	NA	2.49E-06	VKGL	Splice donor	NA	7057+1G>A
1:156134458:G:A	1:156104249	*LMNA*	rs267607571	VCV000066910	NC_000001.11:156134457:G:A	NA	1	NA	2.49E-06	ClinVar	Missense	Arg190Gln	569G>A
1:201361989:G:A	1:201331117	*TNNT2*	rs45586240	VCV000180554	NC_000001.11:201361988:G:A	NA	1	1	2.49E-06	VKGL	Missense	Arg215Trp	643C>T
1:201362016:G:A	1:201331144	*TNNT2*	NA	VCV000181625	NC_000001.11:201362015:G:A	NA	1	1	2.49E-06	ClinVar	Missense	Arg206Trp	616C>T
1:201363349:G:A	1:201332477	*TNNT2*	rs727503512	VCV000228409	NC_000001.11:201363348:G:A	NA	1	1	2.49E-06	ClinVar	Missense	Arg183Trp	547C>T
1:201365291:C:A	1:201334419	*TNNT2*	rs397516457	VCV000043629	NC_000001.11:201365290:C:A	NA	1	1	2.49E-06	ClinVar	Missense	Arg104Leu	311G>T
1:201365638:A:T	1:201334766	*TNNT2*	rs121964855	VCV000012408	NC_000001.11:201365637:A:T	NA	1	1	2.49E-06	ClinVar	Missense	Ile89Asn	266T>A
10:110812573:C:T	10:112572331	*RBM20*	rs1393804220	VCV000538028	NC_000010.11:110812572:C:T	NA	1	NA	2.49E-06	VKGL	Nonsense	Arg726Ter	2176C>T
11:19188245:A:C	11:19209792	*CSRP3*	rs104894204	VCV000008777	NC_000011.10:19188244:A:C	NA	NA	1	2.49E-06	ClinVar	Missense	Cys58Gly	172T>G
11:47332189:G:A	11:47353740	*MYBPC3*	rs397516037	VCV000042735	NC_000011.10:47332188:G:A	NA	NA	1	2.49E-06	ClinVar	Nonsense	Gln1233Ter	3697C>T
11:47333923:T:C	11:47355474	*MYBPC3*	rs727503177	VCV000164052	NC_000011.10:47333922:T:C	NA	NA	1	2.49E-06	VKGL	Missense	Gln998Arg	2993A>G
11:47335081:D:2	11:47356632	*MYBPC3*	rs397515990	VCV000042663	NC_000011.10:47335081:AG:	NA	NA	1	2.49E-06	ClinVar	Frameshift	Pro955fs	2864_2865del
11:47337535:G:A	11:47359086	*MYBPC3*	rs775404728	VCV000195850	NC_000011.10:47337534:G:A	NA	NA	1	2.49E-06	VKGL	Missense	Arg820Trp	2458C>T
11:47341230:G:A	11:47362781	*MYBPC3*	rs730880551	VCV000180950	NC_000011.10:47341229:G:A	NA	NA	1	2.49E-06	ClinVar	Missense	Thr602Ile	1805C>T
11:47342882:D:1	11:47364433	*MYBPC3*	rs886037900	VCV000254153	NC_000011.10:47342882:CCCC:CCC	NA	NA	1	2.49E-06	VKGL	Frameshift	Gln469fs	1404del

SNP	GRCh37	Gene	rsID	Accession ClinVar	Canonical SPDI	N ARVC	N DCM	N HCM	MAF	Origin	Molecular Consequence	Amino acid change	Nucleotide change
11:47343019:A:G	11:47364570	*MYBPC3*	rs397515897	VCV000042525	NC_000011.10:47343018:A:G	NA	NA	1	2.49E-06	ClinVar	Splice donor	NA	1351+2T>C
11:47343147:T:C	11:47364698	*MYBPC3*	rs730880531	VCV000180925	NC_000011.10:47343146:T:C	NA	NA	1	2.49E-06	ClinVar	Splice acceptor	NA	1227-2A>G
11:47343342:C:T	11:47364893	*MYBPC3*	rs1025692267	VCV000693982	NC_000011.10:47343341:C:T	NA	NA	1	2.49E-06	ClinVar	NA	NA	1224-80G>A
11:47343505:G:A	11:47365056	*MYBPC3*	rs727504329	VCV000177796	NC_000011.10:47343504:G:A	NA	NA	1	2.49E-06	ClinVar	Nonsense	Gln404Ter	1210C>T
11:47346217:C:G	11:47367768	*MYBPC3*	rs730880632	VCV000181057	NC_000011.10:47346216:C:G	NA	NA	1	2.49E-06	ClinVar	Missense	Lys360Asn	1080G>C
11:47347661:G:T	11:47369212	*MYBPC3*	rs371711564	VCV000454335	NC_000011.10:47347660:G:T	NA	NA	1	2.49E-06	VKGL	Synonymous	Arg281=	841C>A
11:47347854:C:A	11:47369405	*MYBPC3*	rs727503213	VCV000228869	NC_000011.10:47347853:C:A	NA	NA	1	2.49E-06	VKGL	NA	NA	821+3G>T
12:32821487:D:1	12:32974421	*PKP2*	rs764817683	VCV000202022	NC_000012.12:32821487:GGGG:GGG	1	NA	NA	2.49E-06	VKGL	Frameshift	Lys628fs	1881del
12:32824163:C:G	12:32977097	*PKP2*	rs78897684	VCV000201989	NC_000012.12:32824162:C:G	1	NA	NA	2.49E-06	ClinVar	Splice acceptor	NA	1557-1G>C
12:32850771:D:4	12:33003705	*PKP2*	rs397516993	VCV000045020	NC_000012.12:32850771:TTTGTTT:TTT	1	NA	NA	2.49E-06	VKGL	Nonsense	Lys456_Gln457insTer	1369_1372del
12:32869034:G:A	12:33021968	*PKP2*	rs754912778	VCV000201977	NC_000012.12:32869033:G:A	1	NA	NA	2.49E-06	ClinVar	Nonsense	Arg355Ter	1063C>T
12:32878426:G:A	12:33031360	*PKP2*	NA	VCV000927573	NC_000012.12:32878425:G:A	1	NA	NA	2.49E-06	VKGL	Missense	Pro152Ser	454C>T
12:32878512:C:T	12:33031446	*PKP2*	rs760576804	VCV000196395	NC_000012.12:32878511:C:T	1	NA	NA	2.49E-06	ClinVar	Nonsense	Trp123Ter	368G>A
12:32879021:G:A	12:33031955	*PKP2*	rs121434420	VCV000006754	NC_000012.12:32879020:G:A	1	NA	NA	2.49E-06	ClinVar	Nonsense	Arg79Ter	235C>T
14:23415652:G:A	14:23884861	*MYH7*	rs121913650	VCV000014118	NC_000014.9:23415651:G:A	NA	1	1	2.49E-06	ClinVar	Missense	Arg1712Trp	5134C>T
14:23416129:C:T	14:23885338	*MYH7*	rs730880810	VCV000312894	NC_000014.9:23416128:C:T	NA	1	1	2.49E-06	VKGL	Missense	Glu1610Lys	4828G>A
14:23417174:G:A	14:23886383	*MYH7*	rs45544633	VCV000164284	NC_000014.9:23417173:G:A	NA	1	1	2.49E-06	ClinVar	Missense	Arg1500Trp	4498C>T
14:23417209:T:C	14:23886418	*MYH7*	NA	VCV000920179	NC_000014.9:23417208:T:C	NA	1	1	2.49E-06	VKGL	Missense	Tyr1488Cys	4463A>G
14:23418337:C:T	14:23887546	*MYH7*	rs1275262402	VCV000524974	NC_000014.9:23418336:C:T	NA	1	1	2.49E-06	VKGL	Missense	Glu1348Lys	4042G>A
14:23422267:C:T	14:23891476	*MYH7*	rs587782962	VCV000155814	NC_000014.9:23422266:C:T	NA	1	1	2.49E-06	ClinVar	Missense	Arg1053Gln	3158G>A
14:23423966:C:T	14:23893175	*MYH7*	rs886039204	VCV000264608	NC_000014.9:23423965:C:T	NA	NA	1	2.49E-06	VKGL	Missense	Asp955Asn	2863G>A
14:23426046:G:A	14:23895255	*MYH7*	rs727504240	VCV000177627	NC_000014.9:23426045:G:A	NA	NA	1	2.49E-06	VKGL	Missense	Arg694Cys	2080C>T
14:23427840:C:T	14:23897049	*MYH7*	rs564101364	VCV000264607	NC_000014.9:23427839:C:T	NA	1	1	2.49E-06	VKGL	Missense	Asp545Asn	1633G>A
14:23429279:G:A	14:23898488	*MYH7*	rs3218714	VCV000014102	NC_000014.9:23429278:G:A	NA	1	1	2.49E-06	ClinVar	Missense	Arg403Trp	1207C>T
14:23431426:A:G	14:23900635	*MYH7*	rs397516269	VCV000043106	NC_000014.9:23431425:A:G	NA	1	1	2.49E-06	ClinVar	Missense	Ile263Thr	788T>C
14:23431611:C:T	14:23900820	*MYH7*	rs397516261	VCV000043096	NC_000014.9:23431610:C:T	NA	1	1	2.49E-06	VKGL	Missense	Val236Ile	706G>A
15:34791163:C:T	15:35083364	*ACTC1*	rs121912673	VCV000018323	NC_000015.10:34791162:C:T	NA	1	1	2.49E-06	VKGL	Missense	Arg314His	941G>A
18:31070776:G:A	18:28650742	*DSC2*	rs769022411	VCV000568186	NC_000018.10:31070775:G:A	1	NA	NA	2.49E-06	ClinVar	Nonsense	Gln734Ter	2200C>T
18:31498297:G:A	18:29078260	*DSG2*	rs1568098570	VCV000567764	NC_000018.10:31498296:G:A	1	NA	NA	2.49E-06	ClinVar	Splice donor	NA	45+1G>A
18:31521244:G:C	18:29101207	*DSG2*	rs553299589	VCV000188450	NC_000018.10:31521243:G:C	1	NA	NA	2.49E-06	ClinVar	Splice donor	NA	523+1G>C
18:31522250:G:A	18:29102213	*DSG2*	rs750176752	VCV000410373	NC_000018.10:31522249:G:A	1	NA	NA	2.49E-06	ClinVar	Splice donor	NA	690+1G>A
18:31524554:A:G	18:29104517	*DSG2*	rs121913011	VCV000016815	NC_000018.10:31524553:A:G	1	NA	NA	2.49E-06	ClinVar	Missense	Asn266Ser	797A>G
18:31538849:C:T	18:29118812	*DSG2*	rs794728086	VCV000199810	NC_000018.10:31538848:C:T	1	NA	NA	2.49E-06	ClinVar	Nonsense	Gln584Ter	1750C>T
18:31538922:I:1	18:29118885-29118886	*DSG2*	rs1039633976	VCV000585217	NC_000018.10:31538922:GGG:GGGG	1	NA	NA	2.49E-06	ClinVar	Frameshift	Leu610fs	1826dup
19:55154157:C:T	19:55665525	*TNNI3*	rs397516347	VCV000043381	NC_000019.10:55154156:C:T	NA	1	1	2.49E-06	ClinVar	Missense	Arg141Gln	422G>A
2:178528367:G:A	2:179393094	*TTN*	rs1477669354	VCV000640886	NC_000002.12:178528366:G:A	NA	1	NA	2.49E-06	ClinVar	Nonsense	Arg35762Ter	107284C>T
2:178528797:G:A	2:179393524	*TTN*	rs565675340	VCV000242530	NC_000002.12:178528796:G:A	NA	1	NA	2.49E-06	ClinVar	Nonsense	Arg35652Ter	106954C>T
2:178529959:C:T	2:179394686	*TTN*	rs760915007	VCV000617573	NC_000002.12:178529958:C:T	NA	1	NA	2.49E-06	ClinVar	Splice donor	NA	106531+1G>A
2:178532100:G:A	2:179396827	*TTN*	rs1553488049	VCV000535030	NC_000002.12:178532099:G:A	NA	1	NA	2.49E-06	ClinVar	Nonsense	Arg34839Ter	104515C>T
2:178532202:G:A	2:179396929	*TTN*	rs750519430	VCV000290707	NC_000002.12:178532201:G:A	NA	1	NA	2.49E-06	VKGL	Nonsense	Arg34805Ter	104413C>T
2:178532844:G:A	2:179397571	*TTN*	NA	VCV000934781	NC_000002.12:178532843:G:A	NA	1	NA	2.49E-06	ClinVar	Nonsense	Arg34591Ter	103771C>T
2:178534619:C:T	2:179399346	*TTN*	rs869312068	VCV000223304	NC_000002.12:178534618:C:T	NA	1	NA	2.49E-06	ClinVar	Nonsense	Trp33999Ter	101996G>A
2:178535790:G:A	2:179400517	*TTN*	rs1057518195	VCV000373074	NC_000002.12:178535789:G:A	NA	1	NA	2.49E-06	ClinVar	Nonsense	Arg33609Ter	100825C>T
2:178536357:C:A	2:179401084	*TTN*	rs374920916	VCV000654634	NC_000002.12:178536356:C:A	NA	1	NA	2.49E-06	ClinVar	Nonsense	Glu33464Ter	100390G>T
2:178544357:G:A	2:179409084	*TTN*	NA	VCV001067228	NC_000002.12:178544356:G:A	NA	1	NA	2.49E-06	VKGL	Nonsense	Arg31958Ter	95872C>T
2:178546102:A:G	2:179410829	*TTN*	rs869320740	VCV000132133	NC_000002.12:178546101:A:G	NA	1	NA	2.49E-06	ClinVar	Missense	Cys31712Arg	95134T>C
2:178546476:G:A	2:179411203	*TTN*	rs869311212	VCV000223389	NC_000002.12:178546475:G:A	NA	1	NA	2.49E-06	ClinVar	Nonsense	Arg31619Ter	94855C>T
2:178549309:G:A	2:179414036	*TTN*	rs794729301	VCV000202424	NC_000002.12:178549308:G:A	NA	1	NA	2.49E-06	ClinVar	Nonsense	Arg30773Ter	92317C>T
2:178553039:C:T	2:179417766	*TTN*	rs1060500457	VCV000404812	NC_000002.12:178553038:C:T	NA	1	NA	2.49E-06	ClinVar	Nonsense	Trp29954Ter	89861G>A
2:178554094:G:A	2:179418821	*TTN*	rs886038916	VCV000263764	NC_000002.12:178554093:G:A	NA	1	NA	2.49E-06	ClinVar	Nonsense	Arg29673Ter	89017C>T
2:178559309:A:T	2:179424036	*TTN*	rs397517735	VCV000047458	NC_000002.12:178559308:A:T	NA	1	NA	2.49E-06	ClinVar	Splice donor	NA	86821+2T>A
2:178560055:I:1	2:179424782-179424783	*TTN*	rs1285329277	VCV000519013	NC_000002.12:178560055:TTTTTT:TTTTTTT	NA	1	NA	2.49E-06	ClinVar	Frameshift	Ser28693fs	86076dup
2:178560364:G:A	2:179425091	*TTN*	rs748689777	VCV000488732	NC_000002.12:178560363:G:A	NA	1	NA	2.49E-06	ClinVar	Nonsense	Arg28590Ter	85768C>T
2:178563607:G:A	2:179428334	*TTN*	rs1575649368	VCV000667023	NC_000002.12:178563606:G:A	NA	1	NA	2.49E-06	ClinVar	Nonsense	Arg27509Ter	82525C>T
2:178563892:G:A	2:179428619	*TTN*	rs766840243	VCV000202415	NC_000002.12:178563891:G:A	NA	1	NA	2.49E-06	ClinVar	Nonsense	Arg27414Ter	82240C>T
2:178566838:G:A	2:179431565	*TTN*	rs774411587	VCV000466659	NC_000002.12:178566837:G:A	NA	1	NA	2.49E-06	ClinVar	Nonsense	Arg26432Ter	79294C>T
2:178570158:C:T	2:179434885	*TTN*	rs1553602546	VCV000518953	NC_000002.12:178570157:C:T	NA	1	NA	2.49E-06	ClinVar	Nonsense	Trp25325Ter	75974G>A
2:178570882:G:A	2:179435609	*TTN*	rs794729286	VCV000202406	NC_000002.12:178570881:G:A	NA	1	NA	2.49E-06	ClinVar	Nonsense	Arg25084Ter	75250C>T
2:178573462:D:1	2:179438190	*TTN*	rs727504531	VCV000178908	NC_000002.12:178573462:A:	NA	1	NA	2.49E-06	ClinVar	Frameshift	Asp24224fs	72669del
2:178577762:C:T	2:179442489	*TTN*	NA	VCV000958293	NC_000002.12:178577761:C:T	NA	1	NA	2.49E-06	ClinVar	Nonsense	Trp22888Ter	68664G>A

SNP	GRCh37	Gene	rsID	Accession ClinVar	Canonical SPDI	N ARVC	N DCM	N HCM	MAF	Origin	Molecular Consequence	Amino acid change	Nucleotide change
2:178584552:G:A	2:179449279	*TTN*	rs794729280	VCV000202399	NC_000002.12:178584551:G:A	NA	1	NA	2.49E-06	ClinVar	Nonsense	Arg21667Ter	64999C>T
2:178587418:C:T	2:179452145	*TTN*	NA	VCV000948116	NC_000002.12:178587417:C:T	NA	1	NA	2.49E-06	ClinVar	Splice acceptor	NA	63794-1G>A
2:178590230:G:A	2:179454957	*TTN*	rs869312112	VCV000223377	NC_000002.12:178590229:G:A	NA	1	NA	2.49E-06	ClinVar	Nonsense	Arg20499Ter	61495C>T
2:178593566:A:G	2:179458293	*TTN*	rs869312054	VCV000223287	NC_000002.12:178593565:A:G	NA	1	NA	2.49E-06	ClinVar	Splice donor	NA	58732+2T>C
2:178594198:G:A	2:179458925	*TTN*	rs768073446	VCV000432196	NC_000002.12:178594197:G:A	NA	1	NA	2.49E-06	VKGL	Nonsense	Arg19399Ter	58195C>T
2:178595636:G:A	2:179460363	*TTN*	NA	VCV000839183	NC_000002.12:178595635:G:A	NA	1	NA	2.49E-06	ClinVar	Nonsense	Arg19240Ter	57718C>T
2:178597751:G:A	2:179462478	*TTN*	rs72646831	VCV000047121	NC_000002.12:178597750:G:A	NA	1	NA	2.49E-06	ClinVar	Nonsense	Arg19111Ter	57331C>T
2:178599145:C:T	2:179463872	*TTN*	rs397517624	VCV000047113	NC_000002.12:178599144:C:T	NA	1	NA	2.49E-06	ClinVar	Splice donor	NA	56647+1G>A
2:178601739:G:A	2:179466466	*TTN*	NA	VCV000958943	NC_000002.12:178601738:G:A	NA	1	NA	2.49E-06	ClinVar	Nonsense	Arg18451Ter	55351C>T
2:178604269:G:A	2:179468996	*TTN*	rs747236787	VCV000579797	NC_000002.12:178604268:G:A	NA	1	NA	2.49E-06	ClinVar	Nonsense	Arg18140Ter	54418C>T
2:178605110:G:A	2:179469837	*TTN*	rs1553682168	VCV000466638	NC_000002.12:178605109:G:A	NA	1	NA	2.49E-06	ClinVar	Nonsense	Arg18023Ter	54067C>T
2:178605552:G:A	2:179470279	*TTN*	rs753333359	VCV000534995	NC_000002.12:178605551:G:A	NA	1	NA	2.49E-06	ClinVar	Nonsense	Arg17915Ter	53743C>T
2:178608700:I:4	2:179473427-179473428	*TTN*	rs794729323	VCV000202450	NC_000002.12:178608700:TCAATCA:TCAAT	NA	1	NA	2.49E-06	ClinVar	Nonsense	Glu17437delinsAspTer	52307_52310dup
2:178610089:C:T	2:179474816	*TTN*	rs761807131	VCV000202384	NC_000002.12:178610088:C:T	NA	1	NA	2.49E-06	ClinVar	Splice donor	NA	51436+1G>A
2:178610090:G:A	2:179474817	*TTN*	rs906494713	VCV000691694	NC_000002.12:178610089:G:A	NA	1	NA	2.49E-06	ClinVar	Nonsense	Gln17146Ter	51436C>T
2:178612115:G:A	2:179476842	*TTN*	rs754866489	VCV000202379	NC_000002.12:178612114:G:A	NA	1	NA	2.49E-06	ClinVar	Nonsense	Arg16766Ter	50296C>T
2:178612355:G:A	2:179477082	*TTN*	rs794729265	VCV000202378	NC_000002.12:178612354:G:A	NA	1	NA	2.49E-06	ClinVar	Nonsense	Arg16724Ter	50170C>T
2:178614226:G:A	2:179478953	*TTN*	rs570046043	VCV000636978	NC_000002.12:178614225:G:A	NA	1	NA	2.49E-06	ClinVar	Nonsense	Arg16391Ter	49171C>T
2:178622684:G:A	2:179487411	*TTN*	rs727505350	VCV000180102	NC_000002.12:178622683:G:A	NA	1	NA	2.49E-06	VKGL	Nonsense	Arg14967Ter	44899C>T
2:178629441:G:A	2:179494168	*TTN*	rs770767998	VCV000223369	NC_000002.12:178629440:G:A	NA	1	NA	2.49E-06	VKGL	Nonsense	Arg14762Ter	44284C>T
2:178630250:G:A	2:179494977	*TTN*	rs140743001	VCV000202367	NC_000002.12:178630249:G:A	NA	1	NA	2.49E-06	VKGL	Nonsense	Arg14758Ter	44272C>T
2:178678746:C:T	2:179543473	*TTN*	rs1389908421	VCV000522785	NC_000002.12:178678745:C:T	NA	1	NA	2.49E-06	ClinVar	Splice donor	NA	33826+1G>A
2:178740125:G:A	2:179604852	*TTN*	rs267607158	VCV000012657	NC_000002.12:178740124:G:A	NA	1	NA	2.49E-06	ClinVar	Nonsense	Gln4370Ter	13108C>T
2:178770483:D:2	2:179635211-179635212	*TTN*	rs869312037	VCV000223266	NC_000002.12:178770483:CACA:CA	NA	1	NA	2.49E-06	ClinVar	Frameshift	Ala2770fs	8307_8308del
2:178786129:T:A	2:179650856	*TTN*	rs1554023044	VCV000518936	NC_000002.12:178786128:T:A	NA	1	NA	2.49E-06	ClinVar	Nonsense	Lys697Ter	2089A>T
2:219418463:A:G	2:220283185	*DES*	rs1057523274	VCV000388926	NC_000002.12:219418462:A:G	1	1	NA	2.49E-06	ClinVar	Missense	Met1Val	1A>G
6:118559037:T:G	6:118880200	*PLN*	rs111033560	VCV000013637	NC_000006.12:118559036:T:G	1	**1**	NA	2.49E-06	ClinVar	Nonsense	Leu39Ter	116T>G
6:7555797:C:T	6:7556030	*DSP*	rs768521444	VCV000451211	NC_000006.12:7555796:C:T	1	1	NA	2.49E-06	ClinVar	Nonsense	Arg84Ter	250C>T
6:7558155:C:T	6:7558388	*DSP*	NA	VCV000853704	NC_000006.12:7558154:C:T	1	1	NA	2.49E-06	ClinVar	Nonsense	Arg105Ter	313C>T
6:7559281:C:T	6:7559514	*DSP*	rs397516943	VCV000044922	NC_000006.12:7559280:C:T	1	1	NA	2.49E-06	ClinVar	Nonsense	Arg160Ter	478C>T
6:7568448:I:1	6:7568681-7568682	*DSP*	rs1561687796	VCV000565816	NC_000006.12:7568448:AAAA:AAAAA	1	NA	NA	2.49E-06	ClinVar	Frameshift	Ile428fs	1282dup
6:7568458:G:T	6:7568691	*DSP*	NA	VCV000857798	NC_000006.12:7568457:G:T	1	1	NA	2.49E-06	ClinVar	Nonsense	Glu430Ter	1288G>T
6:7568521:C:G	6:7568754	*DSP*	NA	VCV000948761	NC_000006.12:7568520:C:G	NA	1	NA	2.49E-06	ClinVar	Missense	Arg451Gly	1351C>G
6:7571554:C:T	6:7571787	*DSP*	rs876657638	VCV000228253	NC_000006.12:7571553:C:T	1	1	NA	2.49E-06	ClinVar	Nonsense	Gln625Ter	1873C>T
6:7574797:T:C	6:7575030	*DSP*	rs774514264	VCV000388661	NC_000006.12:7574796:T:C	1	1	NA	2.49E-06	ClinVar	Splice donor	NA	2436+2T>C
6:7579385:C:G	6:7579618	*DSP*	rs886039178	VCV000264512	NC_000006.12:7579384:C:G	1	1	NA	2.49E-06	ClinVar	Nonsense	Tyr1065Ter	3195C>G
6:7580370:C:T	6:7580603	*DSP*	rs140474226	VCV000162505	NC_000006.12:7580369:C:T	1	1	NA	2.49E-06	ClinVar	Nonsense	Gln1394Ter	4180C>T
6:7580721:C:T	6:7580954	*DSP*	rs397516940	VCV000044914	NC_000006.12:7580720:C:T	1	1	NA	2.49E-06	ClinVar	Nonsense	Gln1511Ter	4531C>T
6:7581402:C:T	6:7581635	*DSP*	rs794728124	VCV000199890	NC_000006.12:7581401:C:T	1	1	NA	2.49E-06	ClinVar	Nonsense	Arg1738Ter	5212C>T
6:7583740:C:T	6:7583973	*DSP*	rs777573018	VCV000199902	NC_000006.12:7583739:C:T	1	1	NA	2.49E-06	ClinVar	Nonsense	Arg2160Ter	6478C>T
6:7584904:C:T	6:7585137	*DSP*	NA	VCV000984931	NC_000006.12:7584903:C:T	1	1	NA	2.49E-06	ClinVar	Nonsense	Arg2548Ter	7642C>T
7:128841304:C:T	7:128481358	*FLNC*	rs770606675	VCV000421215	NC_000007.14:128841303:C:T	NA	**1**	NA	2.49E-06	ClinVar	Nonsense	Arg650Ter	1948C>T
7:128846396:C:T	7:128486450	*FLNC*	rs138193236	VCV000070588	NC_000007.14:128846395:C:T	NA	**1**	NA	2.49E-06	ClinVar	Nonsense	Arg1354Ter	4060C>T
7:128853750:C:T	7:128493804	*FLNC*	rs1186464414	VCV000539432	NC_000007.14:128853749:C:T	NA	**1**	NA	2.49E-06	VKGL	Missense	Arg2133Cys	6397C>T
7:128854661:C:T	7:128494715	*FLNC*	rs748416758	VCV000478129	NC_000007.14:128854660:C:T	NA	**1**	NA	2.49E-06	ClinVar	Nonsense	Arg2326Ter	6976C>T

Abbreviations:
ARVC: arrhythmogenic right ventricular cardiomyopathy; DCM: dilated cardiomyopathy; HCM: hypertrophic cardiomyopathy; MAF: minor allele frequency; N: number of carriers.

Supplementary Table V: Prevalence of all genes per cardiomyopathy				
**Cardiomyopathy**	**Gene**	**N**	**Proportion**	**Prevalence**
**ARVC**	*DES*	15	4.3	7.48E-05
	DSC2	42	12.1	2.09E-04
	DSG2	31	8.9	1.55E-04
	DSP	49	14.1	2.44E-04
	JUP	24	6.9	1.20E-04
	PKP2	185	53.3	9.22E-04
	PLN	1	0.3	4.98E-06
**DCM**	*ACTC1*	1	0.1	4.98E-06
	ACTN2	6	0.8	2.99E-05
	BAG3	15	1.9	7.48E-05
	DES	49	6.1	2.44E-04
	DSP	49	6.1	2.44E-04
	FLNC	56	7.0	2.79E-04
	LMNA	42	5.3	2.09E-04
	MYH7	158	19.8	7.87E-04
	NEXN	4	0.5	1.99E-05
	PLN	8	1.0	3.99E-05
	RBM20	5	0.6	2.49E-05
	SCN5A	59	7.4	2.94E-04
	TNNC1	7	0.9	3.49E-05
	TNNI3	35	4.4	1.74E-04
	TNNT2	32	4.0	1.59E-04
	TPM1	2	0.3	9.97E-06
	TTN	272	34.0	1.36E-03
**HCM**	*ACTC1*	1	0.1	4.98E-06
	CSRP3	27	2.0	1.35E-04
	JPH2	6	0.4	2.99E-05
	MYBPC3	723	53.6	3.60E-03
	MYH7	232	17.2	1.16E-03
	MYL2	21	1.6	1.05E-04
	MYL3	1	0.1	4.98E-06
	TNNC1	7	0.5	3.49E-05
	TNNI3	50	3.7	2.49E-04
	TNNT2	274	20.3	1.37E-03
	TPM1	6	0.4	2.99E-05

Abbreviations:
ACTC1: Actin Alpha Cardiac Muscle 1; ACTN2: Alpha-actinin 2;
ARVC: Arrhythmogenic right ventricular cardiomyopathy; BAG3: BAG Cochaperone 3;
CSRP3: Cysteine And Glycine Rich Protein 3; DCM: Dilated cardiomyopathy; DES: Desmin;
DSC2: Desmocollin 2; DSG2: Desmoglein 2; DSG2: Desmoglein 2; DSP: desmoplakin;
FLNC: Filamin-C; HCM: Hypertrophic cardiomyopathy; JPH2: Junctophilin 2;
JUP: Junction Plakoglobin; LMNA: Lamin A/C; MYBPC3: Myosin Binding Protein C3;
MYH7: Myosin Heavy Chain 7; MYL2: Myosin Light Chain 2; MYL3: Myosin Light Chain 3;
N: Number of individuals; NEXN: Nexilin F-Actin Binding Protein; PKP2: Plakophilin 2;
PLN: phospholamban; RBM20: RNA Binding Motif Protein 20;
SCN5A: Sodium Voltage-Gated Channel Alpha Subunit 5;
TNNC1: Troponin C1, Slow Skeletal And Cardiac Type; TNNI3: Troponin I3, Cardiac Type;
TNNT2: Troponin T2, Cardiac Type; TPM1: Tropomyosin 1; TTN: Titin.

Supplementary Table VI: Prevalence of variants associated with the inherited cardiomyopathies			
**Cardiomyopathy**	**Prevalence with overlapping genes**	**Prevalence without overlapping genes**	**Previously reported prevalence range**
ARVC	1:578	1:712	1:143 - 1:1,706[13-15]
DCM	1:251	1:289 (ARVC overlap) / 1:354 (HCM overlap)	1:33 - 1:526[16, 17]
HCM	1:149	1:260	1:164[19]

Abbreviations:
ARVC: arrhythmogenic right ventricular cardiomyopathy; DCM: dilated cardiomyopathy; HCM: hypertrophic cardiomyopathy.

Supplementary Table VII: Results of Fisher's Exact tests

	ARVC G+ vs G-				DCM G+ vs G-				HCM G+ vs G-			
	OR	95% LCI	95% UCI	p-value	OR	95% LCI	95% UCI	p-value	OR	95% LCI	95% UCI	p-value
CARDIOVASCULAR RISK FACTORS												
Diabetes	1.112	0.755	1.592	0.570	0.833	0.626	1.091	0.200	1.280	1.061	1.537	0.008
Hypertension	0.962	0.760	1.212	0.774	1.072	0.919	1.248	0.374	1.045	0.925	1.179	0.482
Hypercholesterolaemia	1.031	0.795	1.326	0.799	1.120	0.946	1.322	0.185	1.181	1.036	1.345	0.011
Ever Smoked	1.223	0.981	1.525	0.068	1.222	1.055	1.416	0.007	0.956	0.849	1.075	0.461
Family heart disease	1.318	1.058	1.643	0.012	1.119	0.966	1.296	0.130	1.066	0.949	1.197	0.280
CARDIAC DISEASE/OUTCOME												
Cardiac problem	2.112	0.416	6.672	0.183	0.912	0.180	2.868	1.000	0.903	0.278	2.289	1.000
Heart failure*	1.432	0.639	2.812	0.305	2.534	1.708	3.671	5.05E-06	1.352	0.899	1.977	0.135
Cardiomyopathy*	2.341	0.460	7.452	0.150	7.590	4.242	13.283	6.94E-11	5.495	3.206	9.306	8.76E-10
Phenotype positive†	1.325	0.351	3.547	0.550	3.664	2.236	5.813	4.88E-07	3.033	1.979	4.560	5.76E-07
Dilated cardiomyopathy*	4.122	0.453	18.056	0.099	8.090	3.078	20.130	2.08E-05	0.529	0.013	3.481	1.000
Hypertrophic cardiomyopathy*	3.599	0.081	26.974	0.265	10.989	3.383	34.746	4.60E-05	18.775	7.903	49.429	3.41E-13
Ventricular arrhythmias	6.198	2.297	14.376	3.27E-04	4.974	2.392	9.752	1.93E-05	1.801	0.717	3.987	0.143
Atrial arrhythmias	1.054	0.415	2.239	0.841	2.273	1.518	3.314	8.18E-05	1.247	0.826	1.830	0.250
Heart arrhythmia	3.231	1.128	7.573	0.015	2.796	1.356	5.321	0.003	0.547	0.144	1.488	0.310
Chronic ischemic heart disease*	1.431	0.971	2.052	0.059	1.281	0.981	1.652	0.058	0.947	0.748	1.186	0.695
Acute myocardial infarction	1.467	0.801	2.494	0.151	1.134	0.730	1.697	0.519	0.892	0.610	1.270	0.606
Cardiac arrest	0.000	0.000	3.299	0.630	2.209	0.756	5.343	0.118	1.090	0.332	2.808	0.804
Angina pectoris	1.497	0.835	2.506	0.120	1.206	0.795	1.772	0.344	1.344	0.987	1.803	0.049
Conduction disorders	1.535	0.645	3.137	0.260	1.497	0.859	2.464	0.136	1.281	0.807	1.960	0.242
Valvular disease	1.322	0.645	2.439	0.373	1.958	1.335	2.801	4.54E-04	1.269	0.883	1.782	0.163
Congenital heart disease	2.059	0.237	8.219	0.267	1.337	0.260	4.342	0.499	1.059	0.269	3.033	0.788
Pulmonary obstructive disease	1.490	0.940	2.266	0.078	1.198	0.860	1.634	0.240	0.848	0.629	1.125	0.280
Cardiovascular death	1.771	0.860	3.286	0.100	1.673	1.038	2.588	0.030	0.733	0.423	1.197	0.268
All-cause mortality	1.068	0.629	1.713	0.712	1.388	1.023	1.852	0.032	0.890	0.668	1.169	0.428

* Used to define P+, therefore not included in some tests.
† Defined as diagnosis of cardiomyopathy, DCM, HCM or heart failure, in absence of chronic ischemic heart disease.

Abbreviations:
ARVC: arrhythmogenic right ventricular cardiomyopathy; DCM: dilated cardiomyopathy;
G+: carriers of likely pathogenic and pathogenic variants associated with one of the cardiomyopathies; HCM: hypertrophic cardiomyopathy;
LCI: lower limit confidence interval; UCI: upper limit confidence interval.

Supplementary Table VII: Results of Fisher's Exact tests

	strict HCM G+ vs G-				ARVC G+P- vs G-P-				DCM G+P- vs G-P-			
	OR	95% LCI	95% UCI	p-value	OR	95% LCI	95% UCI	p-value	OR	95% LCI	95% UCI	p-value
**CARDIOVASCULAR RISK FACTORS**												
Diabetes	0.802	0.601	1.055	0.124	1.108	0.749	1.594	0.566	0.860	0.645	1.130	0.296
Hypertension	1.093	0.938	1.273	0.246	0.954	0.752	1.205	0.728	1.032	0.882	1.207	0.694
Hypercholesterolaemia	1.006	0.846	1.193	0.932	1.006	0.772	1.298	0.949	1.081	0.909	1.281	0.361
Ever Smoked	1.160	1.000	1.344	0.048	1.228	0.983	1.533	0.066	1.233	1.062	1.432	0.006
Family heart disease	1.168	1.008	1.352	0.035	1.286	1.031	1.605	0.024	1.134	0.976	1.316	0.099
**CARDIAC DISEASE/OUTCOME**												
Cardiac problem	1.216	0.315	3.369	0.575	2.227	0.438	7.061	0.166	0.981	0.194	3.097	1.000
Heart failure*	1.733	1.086	2.661	0.015	NA	NA	NA	NA	NA	NA	NA	NA
Cardiomyopathy*	8.647	4.962	14.841	4.40E-13	NA	NA	NA	NA	NA	NA	NA	NA
Phenotype positive†	4.727	3.028	7.216	8.15E-11	NA	NA	NA	NA	NA	NA	NA	NA
Dilated cardiomyopathy*	0.000	0.000	3.756	0.619	NA	NA	NA	NA	NA	NA	NA	NA
Hypertrophic cardiomyopathy*	30.258	12.586	79.971	3.68E-16	NA	NA	NA	NA	NA	NA	NA	NA
Ventricular arrhythmias	3.038	1.208	6.738	0.010	5.846	1.976	14.398	0.001	3.426	1.352	7.682	0.005
Atrial arrhythmias	1.650	1.035	2.530	0.025	1.176	0.462	2.504	0.672	2.117	1.357	3.194	0.001
Heart arrhythmia	0.690	0.138	2.136	0.798	2.797	0.866	7.018	0.042	2.471	1.115	4.941	0.013
Chronic ischemic heart disease*	0.885	0.649	1.187	0.477	NA	NA	NA	NA	NA	NA	NA	NA
Acute myocardial infarction	0.917	0.562	1.425	0.828	1.476	0.806	2.511	0.149	1.121	0.715	1.689	0.586
Cardiac arrest	1.467	0.377	4.128	0.527	0.000	0.000	3.415	0.628	1.938	0.589	5.017	0.195
Angina pectoris	1.288	0.860	1.873	0.175	1.511	0.843	2.531	0.117	1.244	0.819	1.828	0.244
Conduction disorders	1.241	0.674	2.126	0.454	1.650	0.693	3.379	0.165	1.364	0.740	2.342	0.274
Valvular disease	1.517	0.987	2.253	0.045	1.332	0.624	2.527	0.351	1.662	1.072	2.491	0.017
Congenital heart disease	1.335	0.259	4.337	0.499	2.313	0.264	9.341	0.229	1.020	0.117	4.105	1.000
Pulmonary obstructive disease	0.903	0.620	1.279	0.611	1.575	0.993	2.398	0.040	1.090	0.762	1.522	0.600
Cardiovascular death	0.684	0.321	1.294	0.328	1.916	0.929	3.562	0.054	1.454	0.849	2.358	0.153
All-cause mortality	1.097	0.783	1.505	0.562	1.134	0.668	1.820	0.611	1.277	0.920	1.738	0.123

* Used to define P+, therefore not included in some tests.
† Defined as diagnosis of cardiomyopathy, DCM, HCM or heart failure, in absence of chronic ischemic heart disease.

Abbreviations:
ARVC: arrhythmogenic right ventricular cardiomyopathy; DCM: dilated cardiomyopathy;
G+: carriers of likely pathogenic and pathogenic variants associated with one of the cardiomyopathies; HCM: hypertrophic cardiomyopathy;
LCI: lower limit confidence interval; UCI: upper limit confidence interval.

Supplementary Table VII: Results of Fisher's Exact tests

	HCM G+P- vs G-P-				strict HCM G+P- vs G-P-			
	**OR**	**95% LCI**	**95% UCI**	**p-value**	**OR**	**95% LCI**	**95% UCI**	**p-value**
**CARDIOVASCULAR RISK FACTORS**								
**Diabetes**	1.301	1.075	1.566	0.006	0.806	0.599	1.067	0.148
**Hypertension**	1.022	0.903	1.155	0.733	1.051	0.897	1.228	0.528
**Hypercholesterolaemia**	1.179	1.032	1.344	0.014	0.991	0.830	1.179	0.965
**Ever Smoked**	0.968	0.859	1.090	0.591	1.187	1.022	1.380	0.023
**Family heart disease**	1.068	0.949	1.200	0.274	1.175	1.012	1.364	0.032
**CARDIAC DISEASE/OUTCOME**								
**Cardiac problem**	0.967	0.297	2.460	1.000	1.320	0.342	3.673	0.550
**Heart failure***	NA	NA	NA	NA	NA	NA	NA	NA
**Cardiomyopathy***	NA	NA	NA	NA	NA	NA	NA	NA
**Phenotype positive†**	NA	NA	NA	NA	NA	NA	NA	NA
**Dilated cardiomyopathy***	NA	NA	NA	NA	NA	NA	NA	NA
**Hypertrophic cardiomyopathy***	NA	NA	NA	NA	NA	NA	NA	NA
**Ventricular arrhythmias**	1.005	0.257	2.859	1.000	1.718	0.438	4.892	0.306
**Atrial arrhythmias**	1.143	0.723	1.741	0.504	1.431	0.836	2.319	0.156
**Heart arrhythmia**	0.579	0.152	1.577	0.403	0.741	0.148	2.296	0.797
**Chronic ischemic heart disease***	NA	NA	NA	NA	NA	NA	NA	NA
**Acute myocardial infarction**	0.860	0.581	1.235	0.487	0.862	0.516	1.366	0.583
**Cardiac arrest**	1.143	0.348	2.954	0.799	1.561	0.401	4.405	0.339
**Angina pectoris**	1.378	1.011	1.849	0.038	1.341	0.894	1.950	0.136
**Conduction disorders**	1.071	0.632	1.724	0.713	0.910	0.425	1.734	1.000
**Valvular disease**	1.145	0.765	1.665	0.486	1.245	0.751	1.966	0.313
**Congenital heart disease**	1.207	0.305	3.503	0.770	1.545	0.298	5.079	0.451
**Pulmonary obstructive disease**	0.844	0.619	1.130	0.266	0.898	0.606	1.291	0.659
**Cardiovascular death**	0.669	0.365	1.141	0.163	0.531	0.210	1.125	0.105
**All-cause mortality**	0.863	0.638	1.148	0.338	1.061	0.742	1.481	0.729

* Used to define P+, therefore not included in some tests.
† Defined as diagnosis of cardiomyopathy, DCM, HCM or heart failure, in absence of chronic ischemic heart disease.

Abbreviations:
ARVC: arrhythmogenic right ventricular cardiomyopathy; DCM: dilated cardiomyopathy;
G+: carriers of likely pathogenic and pathogenic variants associated with one of the cardiomyopathies; HCM: hypertrophic cardiomyopathy;
LCI: lower limit confidence interval; UCI: upper limit confidence interval.

Supplementary Table VIII: P-values of Mann-Whitney U tests				
	ARVC G+ vs G-	DCM G+ vs G-	HCM G+ vs G-	strict HCM* G+ vs G-
**CARDIOVASCULAR RISK FACTORS**				
BMI	0.990	0.812	0.271	0.621
Mean systolic blood pressure	0.774	0.901	0.258	0.508
Mean diastolic blood pressure	0.341	0.734	0.997	0.776
Total cholesterol	0.369	0.844	0.242	0.666
HDL	0.941	0.357	0.076	0.161
LDL	0.214	0.898	0.484	0.778
MET minutes per week for walking	0.545	0.055	0.233	0.017
MET minutes per week for moderate activity	0.950	0.913	0.578	0.155
MET minutes per week for vigorous activity	0.352	0.963	0.350	0.589
Total MET minutes per week	0.278	0.619	0.980	0.052
**ECG MEASUREMENTS**				
P duration	NA	NA	NA	NA
P axis	NA	NA	NA	NA
PQ interval	NA	NA	NA	NA
QRS duration	NA	NA	NA	NA
R axis	NA	NA	NA	NA
QTC interval	NA	NA	NA	NA
T axis	NA	NA	NA	NA
**CMR MEASUREMENTS**				
RVEDVi	NA	NA	NA	NA
RVESVi	NA	NA	NA	NA
RVSV	NA	NA	NA	NA
RVSVi	NA	NA	NA	NA
RVEF	NA	NA	NA	NA
RVPER	NA	NA	NA	NA
RVPFR	NA	NA	NA	NA
RVPAFR	NA	NA	NA	NA
LVEDVi	NA	NA	NA	NA
LVESVi	NA	NA	NA	NA
LVSV	NA	NA	NA	NA
LVSVi	NA	NA	NA	NA
LVEF	NA	NA	NA	NA
LVPER	NA	NA	NA	NA
LVPFR	NA	NA	NA	NA
LVPAFR	NA	NA	NA	NA
LVEDMi	NA	NA	NA	NA
LVMVR	NA	NA	NA	NA
LVEDV/RVEDV	NA	NA	NA	NA
LVESV/RVESV	NA	NA	NA	NA
peakEcc	NA	NA	NA	NA
TPKEcc	NA	NA	NA	NA
peakEll2Ch	NA	NA	NA	NA
TPKEll2Ch	NA	NA	NA	NA
peakEll4Ch	NA	NA	NA	NA
TPKEll4Ch	NA	NA	NA	NA
Wall thickness segment 1	NA	NA	NA	NA
Wall thickness segment 2	NA	NA	NA	NA
Wall thickness segment 3	NA	NA	NA	NA
Wall thickness segment 4	NA	NA	NA	NA
Wall thickness segment 5	NA	NA	NA	NA
Wall thickness segment 6	NA	NA	NA	NA
Wall thickness segment 7	NA	NA	NA	NA
Wall thickness segment 8	NA	NA	NA	NA
Wall thickness segment 9	NA	NA	NA	NA
Wall thickness segment 10	NA	NA	NA	NA
Wall thickness segment 11	NA	NA	NA	NA
Wall thickness segment 12	NA	NA	NA	NA
Wall thickness segment 13	NA	NA	NA	NA
Wall thickness segment 14	NA	NA	NA	NA
Wall thickness segment 15	NA	NA	NA	NA
Wall thickness segment 16	NA	NA	NA	NA
Global wall thickness	NA	NA	NA	NA
Septal wall thickness	NA	NA	NA	NA
Maximum wall thickness	NA	NA	NA	NA

* strict HCM group: HCM group after excluding carriers of the 3628-41_3628-17del *MYBPC3* and the Arg278Cys 862C>T *TNNT2* variant.

Abbreviations:
ARVC: arrythmogenic right ventricular cardiomyopathy; BMI: body mass index; CMR: cardiac magnetic resonance imaging; DCM: dilated cardiomyopathy; ECG: Electrocardiography; EDVi: indexed end-diastolic volume; EDMi: indexed end-diastolic mass; EF: ejection fraction; ESVi: indexed end-systolic volume; G+: carriers of likely pathogenic and pathogenic variants associated with one of the cardiomyopathies; HCM: hypertrophic cardiomyopathy; HDL: high-density lipoprotein; LDL: low-density lipoprotein; LV: left ventricular; MET: metabolic equivalent of task; MVR: mass to volume ratio; PAFR: peak atrial filling rate; peakEcc: peak circumferential strain; peakEll2Ch: longitudinal strain analyzed in 2-chamber view; peakEll4Ch: longitudinal strain analyzed in 4-chamber view; PER: peak ejection rate; PFR: peak filling rate; RV: right ventricular; SVi: indexed stroke volume; TPKEcc: global time to peak circumferential strain; TPKEll2Ch: global time to longitudinal strain analyzed in 2-chamber view; TPKEll4Ch: global time to longitudinal strain analyzed in 4-chamber view.

Supplementary Table VIII: P-values of Mann-Whitney U tests				
	ARVC G+P- vs G-P-	DCM G+P- vs G-P-	HCM G+P- vs G-P-	strict HCM* G+P- vs G-P-
**CARDIOVASCULAR RISK FACTORS**				
BMI	0.945	0.800	0.317	0.712
Mean systolic blood pressure	0.917	0.913	0.257	0.608
Mean diastolic blood pressure	0.367	0.760	0.934	0.737
Total cholesterol	0.381	0.780	0.211	0.808
HDL	0.999	0.471	0.070	0.161
LDL	0.223	0.991	0.404	0.991
MET minutes per week for walking	0.500	0.084	0.189	0.010
MET minutes per week for moderate activity	0.989	0.965	0.605	0.137
MET minutes per week for vigorous activity	0.434	0.885	0.379	0.557
Total MET minutes per week	0.290	0.722	0.943	0.038
**ECG MEASUREMENTS**				
P duration	0.315	0.304	0.997	0.999
P axis	0.477	0.162	0.085	0.179
PQ interval	0.617	0.989	0.527	0.904
QRS duration	0.385	0.043	0.445	0.436
R axis	0.208	0.156	0.868	0.699
QTC interval	0.255	0.422	0.300	0.270
T axis	0.818	0.572	0.074	0.128
**CMR MEASUREMENTS**				
RVEDVi	0.780	0.058	0.177	0.722
RVESVi	0.707	0.287	0.051	0.118
RVSV	0.713	0.071	0.910	0.140
RVSVi	0.546	0.155	0.872	0.106
RVEF	0.950	0.765	0.025	0.015
RVPER	0.869	0.038	0.711	0.615
RVPFR	0.908	0.120	0.064	0.249
RVPAFR	0.661	0.385	0.192	0.025
LVEDVi	0.060	0.125	0.378	0.444
LVESVi	0.414	0.032	0.276	0.460
LVSV	0.100	0.509	0.889	0.214
LVSVi	0.052	0.430	0.921	0.318
LVEF	0.452	0.009	0.366	0.607
LVPER	0.465	0.023	0.190	0.420
LVPFR	0.114	0.436	0.567	0.485
LVPAFR	0.670	0.412	0.659	0.189
LVEDMi	0.800	0.738	0.928	0.188
LVMVR	0.295	0.061	0.784	0.559
LVEDV/RVEDV	0.360	0.001	0.533	0.747
LVESV/RVESV	0.904	0.000	0.585	0.027
peakEcc	0.319	0.107	0.643	0.812
TPKEcc	0.555	0.155	0.723	0.850
peakEll2Ch	0.751	0.019	0.751	0.179
TPKEll2Ch	0.616	0.701	0.978	0.314
peakEll4Ch	0.483	0.009	0.079	0.286
TPKEll4Ch	0.941	0.708	0.227	0.403
Wall thickness segment 1	0.211	0.439	0.740	0.029
Wall thickness segment 2	0.446	0.022	0.160	0.155
Wall thickness segment 3	0.268	0.144	0.450	0.254
Wall thickness segment 4	0.020	0.626	0.460	0.919
Wall thickness segment 5	0.110	0.132	0.189	0.745
Wall thickness segment 6	0.736	0.363	0.978	0.503
Wall thickness segment 7	0.234	0.435	0.539	0.826
Wall thickness segment 8	0.622	0.251	0.348	0.753
Wall thickness segment 9	0.087	0.438	0.850	0.303
Wall thickness segment 10	0.035	0.508	0.789	0.220
Wall thickness segment 11	0.083	0.484	0.482	0.502
Wall thickness segment 12	0.237	0.108	0.361	0.943
Wall thickness segment 13	0.974	0.828	0.987	0.575
Wall thickness segment 14	0.832	0.243	0.479	0.869
Wall thickness segment 15	0.988	0.277	0.571	0.748
Wall thickness segment 16	0.938	0.423	0.836	0.333
Global wall thickness	0.159	0.232	0.961	0.270
Septal wall thickness	0.229	0.071	0.523	0.174
Maximum wall thickness	0.210	0.621	0.166	0.008

* strict HCM group: HCM group after excluding carriers of the 3628-41_3628-17del *MYBPC3* and the Arg278Cys 862C>T *TNNT2* variant.

Abbreviations:
ARVC: arrythmogenic right ventricular cardiomyopathy; BMI: body mass index; CMR: cardiac magnetic resonance imaging; DCM: dilated cardiomyopathy; ECG: Electrocardiography; EDVi: indexed end-diastolic volume; EDMi: indexed end-diastolic mass; EF: ejection fraction; ESVi: indexed end-systolic volume; G+: carriers of likely pathogenic and pathogenic variants associated with one of the cardiomyopathies; HCM: hypertrophic cardiomyopathy; HDL: high-density lipoprotein; LDL: low-density lipoprotein; LV: left ventricular; MET: metabolic equivalent of task; MVR: mass to volume ratio; PAFR: peak atrial filling rate; peakEcc: peak circumferential strain; peakEll2Ch: longitudinal strain analyzed in 2-chamber view; peakEll4Ch: longitudinal strain analyzed in 4-chamber view; PER: peak ejection rate; PFR: peak filling rate; RV: right ventricular; SVi: indexed stroke volume; TPKEcc: global time to peak circumferential strain; TPKEll2Ch: global time to longitudinal strain analyzed in 2-chamber view; TPKEll4Ch: global time to longitudinal strain analyzed in 4-chamber view.

Supplementary Table IX: Extensive baseline table of P- CMR participants					
	ARVC G+	DCM G+	HCM G+	Controls G-	Missing
n	33	87	130	986	
**Sex = Female (%)**	19 (57.6)	46 (52.9)	62 (47.7)	486 (49.3)	0
**Age (median [IQR])**	54.00 [50.00, 61.00]	55.00 [50.00, 59.50]	54.00 [48.00, 60.00]	55.00 [49.00, 60.00]	0
**Ethnicity (%)**					0
Asian	0 (0.0)	1 (1.1)	18 (13.8)	76 (7.7)	
Black	0 (0.0)	1 (1.1)	2 (1.5)	2 (0.2)	
Chinese	1 (3.0)	1 (1.1)	1 (0.8)	9 (0.9)	
Mixed	0 (0.0)	1 (1.1)	5 (3.8)	15 (1.5)	
Other	0 (0.0)	0 (0.0)	1 (0.8)	5 (0.5)	
White	32 (97.0)	83 (95.4)	103 (79.2)	879 (89.1)	
CARDIOVASCULAR RISK FACTORS					
**BMI (median [IQR])**	26.10 [24.23, 28.72]	26.27 [23.68, 29.11]	26.07 [23.82, 28.82]	25.84 [23.58, 28.62]	0
**Diabetes (%)**	2 (6.1)	7 (8.0)	12 (9.2)	78 (7.9)	0
**Hypertension (%)**	6 (18.2)	27 (31.0)	42 (32.3)	309 (31.3)	0
**Mean systolic blood pressure (median [IQR])**	131.00 [117.50, 141.00]	134.50 [120.75, 146.75]	133.00 [122.00, 146.50]	133.50 [122.00, 146.00]	0.2
**Mean diastolic blood pressure (median [IQR])**	79.50 [73.00, 86.50]	80.25 [74.50, 87.38]	81.50 [76.00, 87.50]	81.00 [74.50, 87.00]	0.2
**Hypercholesterolaemia (%)**	9 (27.3)	26 (29.9)	45 (34.6)	269 (27.3)	0
**Total cholesterol (median [IQR])**	5.39 [4.92, 6.09]	5.51 [4.76, 6.34]	5.66 [4.85, 6.48]	5.63 [4.94, 6.44]	3.5
**HDL (median [IQR])**	1.41 [1.12, 1.63]	1.42 [1.19, 1.61]	1.38 [1.14, 1.62]	1.40 [1.18, 1.71]	8.8
**LDL (median [IQR])**	3.31 [3.00, 4.02]	3.45 [2.87, 4.13]	3.58 [2.89, 4.24]	3.50 [3.00, 4.11]	3.7
**Ever Smoked (%)**	13 (39.4)	45 (51.7)	53 (40.8)	397 (40.3)	0
**MET minutes per week for walking (median [IQR])**	693.00 [309.38, 1,386.00]	462.00 [198.00, 1,188.00]	495.00 [247.50, 1,039.50]	594.00 [247.50, 1,188.00]	14.3
**MET minutes per week for moderate activity (median [IQR])**	360.00 [90.00, 880.00]	570.00 [240.00, 960.00]	360.00 [160.00, 840.00]	360.00 [120.00, 1,080.00]	14.3
**MET minutes per week for vigorous activity (median [IQR])**	240.00 [0.00, 1,110.00]	280.00 [0.00, 960.00]	320.00 [0.00, 960.00]	288.00 [0.00, 960.00]	14.3
**Total MET minutes per week (median [IQR])**	1,840.50 [1,113.00, 2,447.25	1,483.00 [937.50, 3,097.50]	1,436.00 [793.00, 2,559.00]	1,737.00 [736.88, 3,352.50]	14.3
**Family heart disease (%)**	17 (51.5)	47 (54.0)	82 (61.2)	542 (54.7)	0
CARDIAC DISEASE/OUTCOME					
**Cardiac problem (%)**	0 (0.0)	1 (1.1)	1 (0.8)	6 (0.6)	0
**Heart failure (%)**	0 (0.0)	0 (0.0)	1 (0.8)	5 (0.5)	0
**Cardiomyopathy (%)**	0 (0.0)	0 (0.0)	0 (0.0)	0 (0.0)	0
**Dilated cardiomyopathy (%)**	0 (0.0)	0 (0.0)	0 (0.0)	0 (0.0)	0
**Hypertrophic cardiomyopathy (%)**	0 (0.0)	0 (0.0)	0 (0.0)	0 (0.0)	0
**Ventricular arrhythmias (%)**	0 (0.0)	0 (0.0)	0 (0.0)	2 (0.2)	0
**Atrial arrhythmias (%)**	0 (0.0)	5 (5.7)	3 (2.3)	12 (1.2)	0
**Heart arrhythmia (%)**	1 (3.0)	3 (3.4)	0 (0.0)	5 (0.5)	0
**Chronic ischemic heart disease (%)**	2 (6.1)	3 (3.4)	3 (2.3)	43 (4.4)	0
**Acute myocardial infarction (%)**	0 (0.0)	0 (0.0)	1 (0.8)	29 (2.9)	0
**Cardiac arrest (%)**	0 (0.0)	0 (0.0)	0 (0.0)	1 (0.1)	0
**Angina pectoris (%)**	1 (3.0)	2 (2.3)	6 (4.6)	18 (1.8)	0
**Conduction disorders (%)**	0 (0.0)	0 (0.0)	2 (1.5)	10 (1.0)	0
**Valvular disease (%)**	0 (0.0)	2 (2.3)	0 (0.0)	23 (2.3)	0
**Congenital heart disease (%)**	0 (0.0)	0 (0.0)	0 (0.0)	0 (0.0)	0
**Pulmonary obstructive disease (%)**	2 (6.1)	3 (3.4)	4 (3.1)	35 (3.5)	0
**Cardiovascular death (%)**	0 (0.0)	0 (0.0)	1 (0.8)	3 (0.3)	0
**All-cause mortality (%)**	0 (0.0)	1 (1.1)	1 (0.8)	12 (1.2)	0
ECG MEASUREMENTS					
**n (%)**	29 (87.9)	80 (92.0)	110 (84.6)	856 (86.8)	0
**P duration (median [IQR])**	100.00 [92.00, 110.00]	98.00 [86.00, 106.00]	100.00 [90.00, 107.00]	100.00 [90.00, 108.00]	16.3
**P axis (median [IQR])**	54.00 [42.25, 61.50]	48.00 [36.00, 61.00]	49.00 [35.00, 62.50]	55.00 [41.00, 67.00]	37.4
**PQ interval (median [IQR])**	171.00 [147.00, 183.00]	165.00 [144.50, 176.00]	160.00 [146.00, 170.00]	160.00 [144.00, 178.00]	37.5
**QRS duration (median [IQR])**	88.00 [80.00, 94.00]	83.00 [78.00, 92.00]	84.00 [80.00, 92.00]	86.00 [80.00, 94.00]	13
**R axis (median [IQR])**	23.50 [-1.75, 50.00]	27.50 [-2.50, 50.00]	40.00 [13.00, 54.00]	35.50 [8.00, 58.00]	35.4
**QTC interval (median [IQR])**	429.50 [403.25, 440.00]	422.00 [405.50, 435.75]	414.00 [401.00, 429.00]	416.00 [402.00, 432.00]	35.4
**T axis (median [IQR])**	35.50 [20.25, 54.25]	42.00 [26.25, 57.00]	45.00 [31.00, 61.00]	40.00 [23.00, 55.75]	35.4
CMR MEASUREMENTS					
**RVEDVi (median [IQR])**	79.14 [73.73, 92.49]	76.54 [69.50, 84.81]	77.12 [67.27, 90.71]	80.19 [70.61, 90.27]	8.4
**RVESVi (median [IQR])**	35.16 [29.98, 38.71]	32.21 [27.10, 37.43]	31.40 [26.20, 37.28]	32.90 [27.42, 39.70]	8.4
**RVSV (median [IQR])**	87.13 [71.60, 106.86]	82.22 [69.02, 93.57]	86.09 [69.79, 105.70]	85.85 [72.77, 101.86]	8.4
**RVSVi (median [IQR])**	48.22 [41.97, 52.24]	44.50 [40.75, 51.29]	45.77 [40.64, 54.04]	46.53 [40.91, 52.81]	8.4
**RVEF (median [IQR])**	58.30 [53.30, 62.06]	59.31 [52.99, 62.59]	59.56 [54.80, 63.99]	58.37 [54.19, 62.74]	8.7
**RVPER (median [IQR])**	405.50 [291.70, 489.35]	361.23 [290.08, 443.96]	389.50 [310.20, 475.80]	388.65 [316.52, 465.95]	8.4
**RVPFR (median [IQR])**	302.80 [225.65, 375.82]	295.76 [220.44, 343.18]	278.70 [230.80, 334.70]	300.30 [245.10, 363.40]	8.5
**RVPAFR (median [IQR])**	274.70 [213.70, 343.90]	275.00 [224.92, 344.91]	300.10 [236.43, 366.40]	282.86 [222.52, 360.08]	8.4
**LVEDVi (median [IQR])**	80.77 [73.11, 88.68]	77.32 [68.06, 86.15]	72.34 [64.33, 84.59]	74.37 [66.38, 83.15]	17.8
**LVESVi (median [IQR])**	31.74 [25.91, 39.55]	31.69 [26.19, 39.84]	29.37 [24.09, 34.83]	30.02 [25.13, 35.72]	17.8
**LVSV (median [IQR])**	91.46 [74.68, 102.10]	80.50 [68.12, 95.54]	82.92 [64.93, 100.22]	81.72 [70.11, 95.72]	17.6
**LVSVi (median [IQR])**	46.82 [43.25, 50.82]	43.18 [37.50, 49.11]	44.07 [38.21, 50.19]	44.06 [39.37, 50.29]	17.6
**LVEF (median [IQR])**	59.69 [56.59, 66.23]	57.34 [52.60, 62.80]	59.74 [56.25, 63.62]	59.48 [55.29, 63.52]	17.8
**LVPER (median [IQR])**	407.30 [307.20, 455.45]	339.21 [259.00, 430.80]	340.46 [264.61, 460.80]	373.80 [302.47, 453.27]	17.6
**LVPFR (median [IQR])**	346.20 [290.80, 422.04]	314.20 [258.81, 366.80]	320.25 [248.49, 371.20]	321.50 [259.92, 385.64]	17.6
**LVPAFR (median [IQR])**	208.70 [158.60, 298.40]	253.35 [178.90, 330.36]	241.28 [167.28, 321.50]	233.50 [167.46, 305.10]	17.7
**LVEDMi (median [IQR])**	42.81 [36.04, 48.38]	42.96 [36.56, 46.70]	42.41 [34.90, 49.27]	41.85 [36.55, 48.61]	17.7
**LVMVR (median [IQR])**	0.55 [0.49, 0.60]	0.54 [0.49, 0.59]	0.56 [0.50, 0.64]	0.56 [0.50, 0.62]	17.6
**LVEDV/RVEDV (median [IQR])**	0.94 [0.90, 1.05]	1.00 [0.91, 1.08]	0.94 [0.86, 0.99]	0.93 [0.86, 1.03]	20.3
**LVESV/RVESV (median [IQR])**	0.91 [0.82, 1.00]	1.02 [0.89, 1.19]	0.90 [0.82, 1.02]	0.91 [0.80, 1.04]	20.3
**peakEcc (median [IQR])**	-22.87 [-26.91, -21.63]	-22.67 [-24.40, -19.13]	-22.91 [-25.18, -20.88]	-22.72 [-24.98, -20.42]	36.2
**TPKEcc (median [IQR])**	326.90 [318.44, 363.82]	334.75 [320.35, 360.47]	331.96 [308.18, 353.62]	331.30 [309.75, 354.70]	36.4
**peakEll2Ch (median [IQR])**	-21.37 [-23.84, -19.31]	-20.29 [-22.24, -17.97]	-21.54 [-23.50, -18.88]	-21.17 [-23.32, -18.93]	37.3
**TPKEll2Ch (median [IQR])**	346.80 [321.40, 370.50]	353.30 [330.98, 381.65]	353.10 [320.40, 379.60]	349.88 [321.78, 379.08]	37.4
**peakEll4Ch (median [IQR])**	-24.25 [-26.79, -21.38]	-22.30 [-24.57, -19.76]	-24.05 [-26.94, -22.28]	-23.30 [-25.98, -21.37]	38.3
**TPKEll4Ch (median [IQR])**	354.30 [328.00, 406.60]	354.80 [325.40, 392.55]	349.21 [318.52, 390.17]	357.30 [326.94, 397.80]	38.6
**Wall thickness segment 1 (median [IQR])**	7.05 [6.25, 8.57]	7.44 [6.78, 8.21]	7.59 [6.72, 8.55]	7.65 [6.81, 8.49]	31.6
**Wall thickness segment 2 (median [IQR])**	6.81 [5.24, 7.75]	6.03 [5.31, 7.39]	7.06 [5.87, 8.21]	6.75 [5.74, 7.90]	31.6
**Wall thickness segment 3 (median [IQR])**	5.58 [4.74, 7.16]	6.10 [4.85, 6.66]	6.22 [5.04, 7.39]	6.05 [5.17, 6.95]	31.6
**Wall thickness segment 4 (median [IQR])**	6.06 [5.49, 6.76]	6.57 [5.89, 6.99]	6.47 [5.85, 6.92]	6.54 [5.81, 7.21]	31.6
**Wall thickness segment 5 (median [IQR])**	5.96 [5.48, 6.40]	6.08 [5.57, 6.50]	6.03 [5.57, 6.64]	6.20 [5.62, 6.96]	31.6

	ARVC G+	DCM G+	HCM G+	Controls G-	Missing
n	33	87	130	986	
**Wall thickness segment 6 (median [IQR])**	6.45 [6.15, 6.79]	6.58 [6.05, 7.01]	6.56 [5.93, 7.09]	6.55 [5.97, 7.31]	31.6
**Wall thickness segment 7 (median [IQR])**	5.52 [5.21, 6.06]	5.56 [5.28, 6.14]	5.75 [5.21, 6.19]	5.73 [5.29, 6.31]	31.7
**Wall thickness segment 8 (median [IQR])**	6.97 [6.44, 7.28]	6.90 [6.27, 7.47]	6.89 [6.16, 7.49]	7.01 [6.28, 7.75]	31.7
**Wall thickness segment 9 (median [IQR])**	6.96 [6.18, 7.46]	7.18 [6.64, 7.85]	7.21 [6.38, 8.20]	7.38 [6.48, 8.25]	31.7
**Wall thickness segment 10 (median [IQR])**	5.88 [5.33, 6.23]	6.28 [5.90, 6.89]	6.18 [5.44, 7.00]	6.24 [5.60, 6.96]	31.7
**Wall thickness segment 11 (median [IQR])**	5.42 [4.96, 5.81]	5.56 [5.08, 6.08]	5.53 [5.02, 6.37]	5.62 [5.10, 6.32]	31.7
**Wall thickness segment 12 (median [IQR])**	5.38 [5.17, 6.12]	5.50 [5.14, 6.02]	5.59 [5.08, 6.13]	5.60 [5.22, 6.26]	31.7
**Wall thickness segment 13 (median [IQR])**	5.38 [5.24, 5.80]	5.44 [5.13, 5.91]	5.49 [5.12, 5.93]	5.48 [5.10, 5.91]	31.7
**Wall thickness segment 14 (median [IQR])**	5.92 [5.41, 6.60]	5.90 [5.44, 6.28]	5.95 [5.38, 6.58]	6.00 [5.36, 6.66]	31.7
**Wall thickness segment 15 (median [IQR])**	5.12 [4.78, 5.37]	4.99 [4.56, 5.39]	4.99 [4.44, 5.68]	5.09 [4.48, 5.69]	31.7
**Wall thickness segment 16 (median [IQR])**	5.32 [4.86, 5.67]	5.13 [4.82, 5.67]	5.23 [4.76, 5.65]	5.26 [4.80, 5.72]	31.7
**Global wall thickness (median [IQR])**	6.07 [5.57, 6.29]	6.17 [5.90, 6.59]	6.28 [5.70, 6.87]	6.30 [5.72, 6.84]	31.6
**Septal wall thickness (median [IQR])**	6.43 [5.69, 7.01]	6.37 [6.00, 6.90]	6.62 [6.12, 7.52]	6.67 [5.88, 7.34]	31.7
**Maximum wall thickness (median [IQR])**	7.81 [6.97, 8.59]	8.01 [7.43, 8.66]	8.16 [7.45, 9.58]	8.09 [7.24, 9.01]	31.7

Abbreviations:
ARVC: arrythmogenic right ventricular cardiomyopathy; BMI: body mass index; CMR: cardiac magnetic resonance imaging; DCM: dilated cardiomyopathy; ECG: Electrocardiography;
EDVi: indexed end-diastolic volume; EDMi: indexed end-diastolic mass; EF: ejection fraction; ESVi: indexed end-systolic volume;
G+: carriers of likely pathogenic and pathogenic variants associated with one of the cardiomyopathies; HCM: hypertrophic cardiomyopathy; HDL: high-density lipoprotein;
IQR: interquartile range; LDL: low-density lipoprotein; LV: left ventricular; MET: metabolic equivalent of task; MVR: mass to volume ratio; PAFR: peak atrial filling rate;
peakEcc: peak circumferential strain; peakEll2Ch: longitudinal strain analyzed in 2-chamber view; peakEll4Ch: longitudinal strain analyzed in 4-chamber view; PER: peak ejection rate;
PFR: peak filling rate; RV: right ventricular; SVi: indexed stroke volume; TPKEcc: global time to peak circumferential strain;
TPKEll2Ch: global time to longitudinal strain analyzed in 2-chamber view; TPKEll4Ch: global time to longitudinal strain analyzed in 4-chamber view.

Supplementary Table X: Outcome risk stratified by cardiomyopathy and gene

	ARVC G+ vs G-					DCM G+ vs G-					HCM G+ vs G-				
Phenotype	OR	95% LCI	95% UCI	p-value	Gene	OR	95% LCI	95% UCI	p-value	Gene	OR	95% LCI	95% UCI	p-value	Gene
Heart failure	8.270	0.900	36.898	0.030		0.000	0.000	2031.506	1.000		0.000	0.000	2031.506	1.000	
Cardiomyopathy	19.151	0.442	132.223	0.056		0.000	0.000	9000.382	1.000		0.000	0.000	9000.382	1.000	
Heart failure + Cardiomyopathy	7.476	0.814	33.321	0.036		0.000	0.000	1841.531	1.000		0.000	0.000	1841.531	1.000	
Phenotype positive	8.110	0.190	54.456	0.124		0.000	0.000	4144.720	1.000		0.000	0.000	4144.720	1.000	
Ventricular arrhythmias	0.000	0.000	88.063	1.000		0.000	0.000	9925.577	1.000		0.000	0.000	9925.577	1.000	
Atrial arrhythmias	3.657	0.086	24.275	0.253	*DES*	0.000	0.000	1936.952	1.000	*ACTC1*	0.000	0.000	1936.952	1.000	*ACTC1*
Heart arrhythmia	28.201	3.018	128.955	0.003		0.000	0.000	6442.674	1.000		0.000	0.000	6442.674	1.000	
Chronic ischemic heart disease	3.188	0.576	11.850	0.091		0.000	0.000	493.649	1.000		0.000	0.000	493.649	1.000	
Angina pectoris	2.211	0.052	14.617	0.380		0.000	0.000	1185.278	1.000		0.000	0.000	1185.278	1.000	
Cardiovascular death	3.863	0.091	25.642	0.241		0.000	0.000	2042.573	1.000		0.000	0.000	2042.573	1.000	
All-cause mortality	2.836	0.310	12.580	0.179		0.000	0.000	711.200	1.000		0.000	0.000	711.200	1.000	
Heart failure	1.312	0.032	7.822	0.540		10.751	0.226	96.809	0.105		0.000	0.000	7.948	1.000	
Cardiomyopathy	0.000	0.000	25.853	1.000		0.000	0.000	235.208	1.000		0.000	0.000	41.147	1.000	
Heart failure + Cardiomyopathy	1.186	0.029	7.061	0.576		9.715	0.205	87.209	0.116		0.000	0.000	7.178	1.000	
Phenotype positive	0.000	0.000	10.646	1.000		0.000	0.000	97.932	1.000		0.000	0.000	16.960	1.000	
Ventricular arrhythmias	0.000	0.000	29.170	1.000		0.000	0.000	264.754	1.000		0.000	0.000	46.435	1.000	
Atrial arrhythmias	0.000	0.000	4.745	1.000	*DSC2*	0.000	0.000	43.769	1.000	*ACTN2*	0.000	0.000	7.565	1.000	*CSRP3*
Heart arrhythmia	4.479	0.109	27.435	0.207		0.000	0.000	159.535	1.000		0.000	0.000	27.753	1.000	
Chronic ischemic heart disease	0.638	0.075	2.468	0.767		2.551	0.054	22.836	0.365		0.491	0.012	2.995	0.719	
Angina pectoris	0.755	0.019	4.480	1.000		0.000	0.000	26.400	1.000		0.000	0.000	4.558	1.000	
Cardiovascular death	1.319	0.032	7.866	0.538		10.812	0.227	97.361	0.105		0.000	0.000	7.993	1.000	
All-cause mortality	0.922	0.108	3.572	1.000		3.687	0.078	33.037	0.272		0.000	0.000	2.709	0.399	
Heart failure	0.000	0.000	6.863	1.000		8.270	0.900	36.898	0.030		0.000	0.000	45.996	1.000	
Cardiomyopathy	0.000	0.000	35.539	1.000		41.175	4.362	192.166	0.002		0.000	0.000	235.208	1.000	
Heart failure + Cardiomyopathy	0.000	0.000	6.196	1.000		7.476	0.814	33.321	0.036		0.000	0.000	41.524	1.000	
Phenotype positive	0.000	0.000	14.641	1.000		17.455	1.885	78.723	0.008		0.000	0.000	97.932	1.000	
Ventricular arrhythmias	0.000	0.000	40.048	1.000		21.465	0.494	148.888	0.050		0.000	0.000	264.754	1.000	
Atrial arrhythmias	0.000	0.000	6.530	1.000	*DSG2*	3.657	0.086	24.275	0.253	*BAG3*	10.231	0.215	92.112	0.110	*JPH2*
Heart arrhythmia	0.000	0.000	23.957	1.000		13.101	0.305	89.207	0.080		0.000	0.000	159.535	1.000	
Chronic ischemic heart disease	2.453	0.733	6.515	0.072		0.911	0.022	6.004	1.000		2.551	0.054	22.836	0.365	
Angina pectoris	2.135	0.246	8.503	0.254		0.000	0.000	8.675	1.000		15.464	1.394	108.566	0.014	
Cardiovascular death	0.000	0.000	6.902	1.000		3.863	0.091	25.642	0.241		0.000	0.000	46.258	1.000	
All-cause mortality	1.272	0.147	5.051	0.673		1.317	0.031	8.689	0.548		0.000	0.000	15.706	1.000	
Heart failure	1.121	0.028	6.628	0.596		3.507	0.691	11.071	0.061		1.609	0.966	2.554	0.047	
Cardiomyopathy	5.591	0.135	34.667	0.170		5.591	0.135	34.667	0.170		6.075	3.139	11.256	2.30E-07	
Heart failure + Cardiomyopathy	2.068	0.242	7.995	0.261		3.170	0.625	9.992	0.077		2.547	1.720	3.681	4.50E-06	
Phenotype positive	4.833	0.560	18.977	0.070		2.367	0.058	14.183	0.352		3.732	2.234	6.010	9.20E-07	
Ventricular arrhythmias	0.000	0.000	24.850	1.000		12.800	1.448	52.549	0.013		2.097	0.637	5.428	0.180	
Atrial arrhythmias	0.000	0.000	4.042	1.000	*DSP*	4.551	1.177	12.667	0.015	*DES*	1.011	0.540	1.751	0.889	*MYBPC3*
Heart arrhythmia	0.000	0.000	14.836	1.000		7.811	0.897	31.194	0.031		0.509	0.060	1.939	0.588	
Chronic ischemic heart disease	2.126	0.803	4.798	0.088		1.780	0.617	4.218	0.169		1.030	0.757	1.375	0.824	
Angina pectoris	2.752	0.714	7.618	0.068		0.645	0.016	3.797	1.000		1.294	0.846	1.913	0.188	
Cardiovascular death	2.302	0.269	8.907	0.225		1.127	0.028	6.666	0.594		0.529	0.209	1.118	0.106	
All-cause mortality	0.785	0.092	3.011	1.000		1.202	0.238	3.761	0.740		0.660	0.420	0.995	0.043	
Heart failure	0.000	0.000	9.023	1.000		1.121	0.028	6.628	0.596		1.673	0.656	3.576	0.208	
Cardiomyopathy	0.000	0.000	46.694	1.000		5.591	0.135	34.667	0.170		9.583	3.810	21.218	6.60E-06	
Heart failure + Cardiomyopathy	0.000	0.000	8.143	1.000		2.068	0.242	7.995	0.261		3.360	1.814	5.798	1.20E-04	
Phenotype positive	0.000	0.000	19.247	1.000		4.833	0.560	18.977	0.070		5.116	2.338	10.038	6.80E-05	
Ventricular arrhythmias	0.000	0.000	52.650	1.000		0.000	0.000	24.850	1.000		2.618	0.303	10.344	0.189	
Atrial arrhythmias	0.000	0.000	8.586	1.000	*JUP*	0.000	0.000	4.042	1.000	*DSP*	1.829	0.769	3.740	0.094	*MYH7*
Heart arrhythmia	0.000	0.000	31.449	1.000		0.000	0.000	14.836	1.000		0.795	0.020	4.666	1.000	
Chronic ischemic heart disease	0.555	0.013	3.425	1.000		2.126	0.803	4.798	0.088		1.008	0.573	1.665	0.899	
Angina pectoris	1.346	0.033	8.345	0.534		2.752	0.714	7.618	0.068		1.395	0.653	2.648	0.337	
Cardiovascular death	0.000	0.000	9.074	1.000		3.527	0.695	11.134	0.061		0.949	0.254	2.506	1.000	
All-cause mortality	0.000	0.000	3.074	0.633		1.202	0.238	3.761	0.740		1.458	0.827	2.413	0.135	
Heart failure	1.502	0.476	3.637	0.395		3.044	0.603	9.532	0.084		0.000	0.000	10.428	1.000	
Cardiomyopathy	1.467	0.036	8.797	0.501		0.000	0.000	19.186	1.000		0.000	0.000	53.909	1.000	
Heart failure + Cardiomyopathy	1.638	0.586	3.700	0.281		2.751	0.545	8.603	0.105		0.000	0.000	9.415	1.000	
Phenotype positive	0.621	0.015	3.596	1.000		4.207	0.489	16.404	0.088		0.000	0.000	22.239	1.000	
Ventricular arrhythmias	11.900	4.383	27.862	6.40E-06		0.000	0.000	21.656	1.000		0.000	0.000	60.833	1.000	
Atrial arrhythmias	1.726	0.617	3.902	0.174	*PKP2*	1.897	0.222	7.284	0.293	*FLNC*	2.560	0.061	16.167	0.335	*MYL2*
Heart arrhythmia	3.044	0.603	9.522	0.084		0.000	0.000	12.921	1.000		0.000	0.000	36.379	1.000	
Chronic ischemic heart disease	1.298	0.734	2.157	0.315		0.981	0.257	2.678	1.000		1.343	0.151	5.582	0.663	
Angina pectoris	1.224	0.481	2.606	0.522		0.000	0.000	2.121	0.422		1.548	0.037	9.738	0.488	
Cardiovascular death	2.139	0.836	4.591	0.086		2.003	0.235	7.700	0.272		0.000	0.000	10.487	1.000	
All-cause mortality	1.172	0.571	2.169	0.612		2.634	1.001	5.884	0.025		0.922	0.022	5.787	1.000	
Heart failure	0.000	0.000	2031.506	1.000		1.312	0.032	7.822	0.540		0.000	0.000	2031.506	1.000	
Cardiomyopathy	0.000	0.000	9000.382	1.000		0.000	0.000	25.853	1.000		0.000	0.000	9000.382	1.000	
Heart failure + Cardiomyopathy	0.000	0.000	1841.531	1.000		1.186	0.029	7.061	0.576		0.000	0.000	1841.531	1.000	
Phenotype positive	0.000	0.000	4144.720	1.000		0.000	0.000	10.646	1.000		0.000	0.000	4144.720	1.000	
Ventricular arrhythmias	0.000	0.000	9925.577	1.000		15.041	1.692	62.322	0.009		0.000	0.000	9925.577	1.000	
Atrial arrhythmias	0.000	0.000	1936.952	1.000	*PLN*	2.560	0.298	9.995	0.194	*LMNA*	0.000	0.000	1936.952	1.000	*MYL3*
Heart arrhythmia	0.000	0.000	6442.674	1.000		14.115	2.708	46.576	0.002		0.000	0.000	6442.674	1.000	
Chronic ischemic heart disease	0.000	0.000	493.649	1.000		1.343	0.347	3.744	0.545		0.000	0.000	493.649	1.000	
Angina pectoris	0.000	0.000	1185.278	1.000		0.755	0.019	4.480	1.000		0.000	0.000	1185.278	1.000	
Cardiovascular death	0.000	0.000	2042.573	1.000		0.000	0.000	5.015	1.000		0.000	0.000	2042.573	1.000	
All-cause mortality	0.000	0.000	711.200	1.000		0.450	0.011	2.662	0.724		0.000	0.000	711.200	1.000	
Heart failure						1.758	0.556	4.268	0.219		8.961	0.194	74.405	0.122	
Cardiomyopathy						12.440	4.605	28.856	4.60E-06		0.000	0.000	192.656	1.000	
Heart failure + Cardiomyopathy						3.994	1.985	7.346	1.20E-04		8.097	0.175	67.326	0.133	
Phenotype positive						6.057	2.490	12.775	1.10E-04		0.000	0.000	79.918	1.000	
Ventricular arrhythmias						3.860	0.445	15.325	0.103		0.000	0.000	216.918	1.000	
Atrial arrhythmias						2.021	0.721	4.584	0.132	*MYH7*	0.000	0.000	35.791	1.000	*TNNC1*
Heart arrhythmia						1.170	0.029	6.896	0.580		0.000	0.000	130.632	1.000	
Chronic ischemic heart disease						1.240	0.658	2.163	0.439		2.126	0.046	17.555	0.411	
Angina pectoris						1.870	0.831	3.693	0.101		5.158	0.112	42.747	0.200	
Cardiovascular death						1.047	0.212	3.166	0.765		0.000	0.000	37.806	1.000	
All-cause mortality						1.653	0.853	2.943	0.100		7.373	0.700	45.159	0.047	
Heart failure						0.000	0.000	82.035	1.000		0.000	0.000	4.160	1.000	
Cardiomyopathy						0.000	0.000	415.844	1.000		5.478	0.132	33.866	0.173	
Heart failure + Cardiomyopathy						0.000	0.000	73.980	1.000		0.992	0.025	5.856	1.000	
Phenotype positive						0.000	0.000	174.556	1.000		2.318	0.057	13.880	0.357	
Ventricular arrhythmias						0.000	0.000	465.410	1.000		6.143	0.148	38.292	0.157	
Atrial arrhythmias						0.000	0.000	77.944	1.000	*NEXN*	1.045	0.026	6.172	0.621	*TNNI3*
Heart arrhythmia						0.000	0.000	282.879	1.000		0.000	0.000	14.529	1.000	
Chronic ischemic heart disease						0.000	0.000	19.338	1.000		0.814	0.162	2.539	1.000	
Angina pectoris						0.000	0.000	47.053	1.000		1.976	0.391	6.192	0.207	
Cardiovascular death						0.000	0.000	82.505	1.000		1.104	0.027	6.523	0.601	
All-cause mortality						0.000	0.000	27.978	1.000		1.177	0.234	3.676	0.743	
Heart failure						0.000	0.000	31.741	1.000		1.000	0.318	2.406	1.000	
Cardiomyopathy						0.000	0.000	163.294	1.000		1.974	0.229	7.721	0.280	
Heart failure + Cardiomyopathy						0.000	0.000	28.673	1.000		1.088	0.391	2.442	0.826	
Phenotype positive						0.000	0.000	67.578	1.000		0.416	0.010	2.401	0.733	
Ventricular arrhythmias						0.000	0.000	183.598	1.000		0.000	0.000	4.318	1.000	
Atrial arrhythmias						0.000	0.000	30.192	1.000	*PLN*	1.343	0.527	2.859	0.376	*TNNT2*
Heart arrhythmia						0.000	0.000	110.596	1.000		0.673	0.017	3.943	1.000	
Chronic ischemic heart disease						0.000	0.000	7.488	1.000		0.791	0.443	1.319	0.410	
Angina pectoris						0.000	0.000	18.219	1.000		1.173	0.550	2.220	0.596	
Cardiovascular death						0.000	0.000	31.922	1.000		1.418	0.557	3.023	0.355	
All-cause mortality						0.000	0.000	10.830	1.000		1.068	0.585	1.812	0.781	

	ARVC G+ vs G-					DCM G+ vs G-					HCM G+ vs G-				
**Phenotype**	**OR**	**95% LCI**	**95% UCI**	**p-value**	**Gene**	**OR**	**95% LCI**	**95% UCI**	**p-value**	**Gene**	**OR**	**95% LCI**	**95% UCI**	**p-value**	**Gene**
**Heart failure**						0.000	0.000	59.126	1.000		0.000	0.000	45.996	1.000	
**Cardiomyopathy**						0.000	0.000	301.276	1.000		0.000	0.000	235.208	1.000	
**Heart failure + Cardiomyopathy**						0.000	0.000	53.348	1.000		0.000	0.000	41.524	1.000	
**Phenotype positive**						0.000	0.000	125.163	1.000		0.000	0.000	97.932	1.000	
**Ventricular arrhythmias**						0.000	0.000	339.088	1.000		0.000	0.000	264.754	1.000	
**Atrial arrhythmias**						0.000	0.000	56.235	1.000	*RBM20*	0.000	0.000	43.769	1.000	*TPM1*
**Heart arrhythmia**						0.000	0.000	204.364	1.000		0.000	0.000	159.535	1.000	
**Chronic ischemic heart disease**						0.000	0.000	13.944	1.000		0.000	0.000	10.853	1.000	
**Angina pectoris**						0.000	0.000	33.910	1.000		0.000	0.000	26.400	1.000	
**Cardiovascular death**						0.000	0.000	59.466	1.000		0.000	0.000	46.258	1.000	
**All-cause mortality**						0.000	0.000	20.179	1.000		0.000	0.000	15.706	1.000	
**Heart failure**						0.927	0.023	5.440	1.000						
**Cardiomyopathy**						0.000	0.000	18.183	1.000						
**Heart failure + Cardiomyopathy**						0.838	0.021	4.912	1.000						
**Phenotype positive**						1.959	0.048	11.638	0.406						
**Ventricular arrhythmias**						5.191	0.126	32.118	0.182						
**Atrial arrhythmias**						0.883	0.022	5.177	1.000	*SCN5A*					
**Heart arrhythmia**						3.166	0.077	19.105	0.278						
**Chronic ischemic heart disease**						1.444	0.505	3.372	0.444						
**Angina pectoris**						0.000	0.000	2.010	0.265						
**Cardiovascular death**						0.000	0.000	3.525	0.629						
**All-cause mortality**						0.988	0.197	3.055	1.000						
**Heart failure**						8.961	0.194	74.405	0.122						
**Cardiomyopathy**						0.000	0.000	192.656	1.000						
**Heart failure + Cardiomyopathy**						8.097	0.175	67.326	0.133						
**Phenotype positive**						0.000	0.000	79.918	1.000						
**Ventricular arrhythmias**						0.000	0.000	216.918	1.000						
**Atrial arrhythmias**						0.000	0.000	35.791	1.000	*TNNC1*					
**Heart arrhythmia**						0.000	0.000	130.632	1.000						
**Chronic ischemic heart disease**						2.126	0.046	17.555	0.411						
**Angina pectoris**						5.158	0.112	42.747	0.200						
**Cardiovascular death**						0.000	0.000	37.806	1.000						
**All-cause mortality**						7.373	0.700	45.159	0.047						
**Heart failure**						0.000	0.000	6.036	1.000						
**Cardiomyopathy**						0.000	0.000	31.281	1.000						
**Heart failure + Cardiomyopathy**						0.000	0.000	5.451	1.000						
**Phenotype positive**						0.000	0.000	12.879	1.000						
**Ventricular arrhythmias**						8.852	0.212	56.217	0.112						
**Atrial arrhythmias**						1.506	0.037	9.076	0.493	*TNNI3*					
**Heart arrhythmia**						0.000	0.000	21.072	1.000						
**Chronic ischemic heart disease**						1.196	0.234	3.836	0.739						
**Angina pectoris**						2.902	0.566	9.356	0.097						
**Cardiovascular death**						1.591	0.039	9.594	0.475						
**All-cause mortality**						1.117	0.130	4.390	0.701						
**Heart failure**						1.735	0.042	10.530	0.447						
**Cardiomyopathy**						8.655	0.207	54.922	0.115						
**Heart failure + Cardiomyopathy**						3.240	0.373	12.920	0.137						
**Phenotype positive**						3.664	0.089	22.504	0.247						
**Ventricular arrhythmias**						0.000	0.000	38.788	1.000						
**Atrial arrhythmias**						1.652	0.040	10.021	0.463	*TNNT2*					
**Heart arrhythmia**						0.000	0.000	23.166	1.000						
**Chronic ischemic heart disease**						1.319	0.257	4.272	0.504						
**Angina pectoris**						0.999	0.024	6.034	1.000						
**Cardiovascular death**						3.605	0.414	14.395	0.116						
**All-cause mortality**						1.907	0.371	6.184	0.227						
**Heart failure**						0.000	0.000	287.327	1.000						
**Cardiomyopathy**						0.000	0.000	1454.551	1.000						
**Heart failure + Cardiomyopathy**						0.000	0.000	259.607	1.000						
**Phenotype positive**						0.000	0.000	604.765	1.000						
**Ventricular arrhythmias**						0.000	0.000	1644.031	1.000						
**Atrial arrhythmias**						0.000	0.000	273.509	1.000	*TPM1*					
**Heart arrhythmia**						0.000	0.000	987.049	1.000						
**Chronic ischemic heart disease**						0.000	0.000	67.908	1.000						
**Angina pectoris**						0.000	0.000	165.176	1.000						
**Cardiovascular death**						0.000	0.000	288.947	1.000						
**All-cause mortality**						0.000	0.000	98.466	1.000						
**Heart failure**						3.585	2.012	6.014	2.20E-05						
**Cardiomyopathy**						10.241	4.492	21.287	3.20E-07						
**Heart failure + Cardiomyopathy**						3.444	1.969	5.695	2.10E-05						
**Phenotype positive**						3.442	1.426	7.187	0.004						
**Ventricular arrhythmias**						4.494	1.148	12.764	0.016						
**Atrial arrhythmias**						3.200	1.766	5.433	1.20E-04	*TTN*					
**Heart arrhythmia**						2.741	0.716	7.515	0.068						
**Chronic ischemic heart disease**						1.178	0.728	1.823	0.477						
**Angina pectoris**						1.182	0.554	2.238	0.595						
**Cardiovascular death**						2.064	0.962	3.946	0.037						
**All-cause mortality**						1.229	0.699	2.027	0.404						

Abbreviations:

ACTC1 : Actin Alpha Cardiac Muscle 1; *ACTN2* : Alpha-actinin 2; ARVC: Arrhythmogenic right ventricular cardiomyopathy; *BAG3* : BAG Cochaperone 3; *CSRP3* : Cysteine And Glycine Rich Protein 3; DCM: Dilated cardiomyopathy; *DES* : Desmin; *DSC2* : Desmocollin 2; *DSG2* : Desmoglein 2; *DSG2* : Desmoglein 2; *DSP* : desmoplakin; *FLNC* : Filamin-C; G+: carriers of likely pathogenic and pathogenic variants associated with one of the cardiomyopathies;
HCM: Hypertrophic cardiomyopathy; *JPH2* : Junctophilin 2; *JUP* : Junction Plakoglobin; LCI: lower limit confidence interval; *LMNA* : Lamin A/C; *MYBPC3* : Myosin Binding Protein C3; *MYH7* : Myosin Heavy Chain 7;
NEXN: Nexilin F-Actin Binding Protein; PKP2 : Plakophilin 2; *PLN* : phospholamban; *RBM20* : RNA Binding Motif Protein 20; *SCN5A* : Sodium Voltage-Gated Channel Alpha Subunit 5; *TNNC1* : Troponin C1, Slow Skeletal And Cardiac Type; *TNNI3: Troponin I3, Cardiac Type; TNNT2* : Troponin T2, Cardiac Type; *TPM1* : Tropomyosin 1; *TTN* : Titin; UCI: upper limit confidence interval.

Supplementary Table XI: Results of Fisher's Exact tests when excluding overlapping genes																
	ARVC G+ vs G-				DCM G+ vs G- (without ARVC genes)				DCM G+ vs G- (without HCM genes)				HCM G+ vs G-			
	OR	95% LCI	95% UCI	p-value	OR	95% LCI	95% UCI	p-value	OR	95% LCI	95% UCI	p-value	OR	95% LCI	95% UCI	p-value
**CARDIOVASCULAR RISK FACTORS**																
**Diabetes**	1.136	0.741	1.684	0.530	0.819	0.602	1.095	0.194	0.835	0.595	1.147	0.293	1.682	1.348	2.083	4.20E-06
**Hypertension**	0.989	0.762	1.276	0.949	1.092	0.927	1.285	0.283	0.999	0.831	1.198	1.000	1.025	0.875	1.197	0.753
**Hypercholesterolaemia**	1.033	0.774	1.365	0.833	1.137	0.949	1.356	0.156	1.057	0.864	1.288	0.579	1.192	1.006	1.407	0.041
**Ever Smoked**	1.158	0.905	1.479	0.244	1.223	1.045	1.432	0.011	1.192	1.002	1.418	0.044	0.869	0.745	1.013	0.069
**Family heart disease**	1.386	1.086	1.770	0.007	1.134	0.969	1.327	0.114	1.074	0.902	1.277	0.434	1.010	0.869	1.173	0.910
**CARDIAC DISEASE/OUTCOME**																
**Cardiac problem**	2.604	0.513	8.240	0.120	1.052	0.208	3.309	0.762	1.291	0.255	4.065	0.511	1.262	0.327	3.497	0.563
**Heart failure**	1.169	0.420	2.628	0.650	2.600	1.713	3.837	8.40E-06	3.010	1.955	4.497	1.00E-06	1.504	0.903	2.386	0.097
**Cardiomyopathy**	0.956	0.023	5.708	1.000	7.963	4.354	14.167	1.70E-10	6.807	3.379	12.983	2.90E-07	5.681	2.936	10.526	5.20E-07
**Dilated cardiomyopathy**	2.531	0.060	16.741	0.342	8.290	3.003	21.263	4.40E-05	11.486	4.365	28.657	1.40E-06	0.923	0.022	6.079	1.000
**Hypertrophic cardiomyopathy**	0.000	0.000	20.787	1.000	10.856	3.095	35.820	1.40E-04	2.204	0.050	16.486	0.39	17.990	6.569	51.615	1.10E-08
**Ventricular arrhythmias**	7.664	2.835	17.818	9.50E-05	4.849	2.201	9.888	8.70E-05	5.415	2.368	11.317	7.10E-05	1.963	0.597	5.081	0.192
**Atrial arrhythmias**	1.113	0.400	2.499	0.663	2.313	1.507	3.444	1.40E-04	2.465	1.556	3.766	1.10E-04	1.015	0.554	1.727	0.892
**Heart arrhythmia**	2.642	0.690	7.242	0.075	2.685	1.213	5.356	0.008	3.639	1.706	7.089	0.001	0.477	0.056	1.815	0.435
**Chronic ischemic heart disease**	1.241	0.783	1.889	0.297	1.207	0.901	1.594	0.176	1.318	0.964	1.770	0.069	1.017	0.754	1.349	0.886
**Acute myocardial infarction**	1.443	0.728	2.600	0.215	1.163	0.728	1.780	0.490	1.005	0.573	1.653	0.900	0.863	0.517	1.368	0.659
**Cardiac arrest**	0.000	0.000	4.064	1.000	2.549	0.872	6.170	0.043	2.080	0.534	5.863	0.145	1.140	0.224	3.638	0.747
**Angina pectoris**	1.257	0.613	2.316	0.486	1.157	0.732	1.756	0.500	0.901	0.505	1.500	0.803	1.252	0.824	1.839	0.242
**Conduction disorders**	1.899	0.797	3.891	0.084	1.633	0.921	2.723	0.080	1.409	0.708	2.554	0.289	1.289	0.700	2.208	0.361
**Valvular disease**	1.179	0.498	2.394	0.558	2.016	1.345	2.937	0.001	1.866	1.173	2.852	0.006	1.074	0.640	1.708	0.716
**Congenital heart disease**	2.536	0.291	10.141	0.199	1.542	0.299	5.010	0.452	1.259	0.145	5.016	0.675	1.385	0.269	4.500	0.486
**Pulmonary obstructive disease**	1.704	1.051	2.643	0.026	1.205	0.845	1.677	0.280	1.112	0.739	1.618	0.551	0.695	0.450	1.033	0.081
**Cardiovascular death**	1.579	0.665	3.223	0.254	1.605	0.951	2.574	0.058	1.879	1.097	3.049	0.016	0.495	0.196	1.046	0.064
**All-cause mortality**	1.036	0.567	1.756	0.891	1.432	1.037	1.940	0.022	1.290	0.888	1.826	0.145	0.643	0.413	0.961	0.032

Abbreviations:
ARVC: arrhythmogenic right ventricular cardiomyopathy; DCM: dilated cardiomyopathy; G+: carriers of likely pathogenic and pathogenic variants associated with one of the cardiomyopathies; HCM: hypertrophic cardiomyopathy;
LCI: lower limit confidence interval; UCI: upper limit confidence interval.

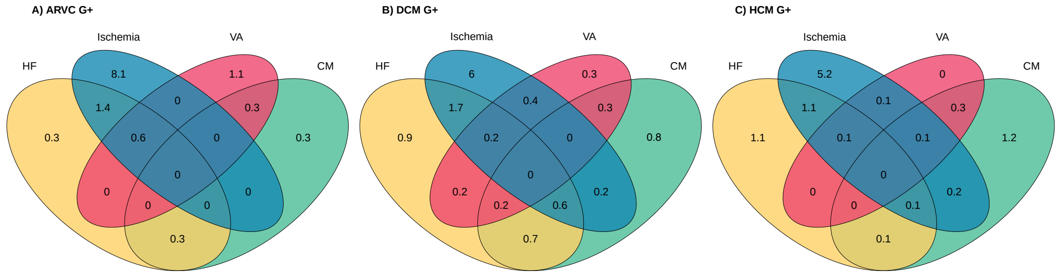

Figure I: Overlap cardiac diagnoses per inherited cardiomyopathy

The Venn diagram of the overlap between cardiomyopathy, heart failure, ventricular arrhythmia and chronic ischemic heart diagnoses in G+ individuals. The numbers in the diagram are the percentages of individuals diagnosed in the G+ of the specified cardiomyopathy.

Abbreviations: ARVC= arrhythmogenic right ventricular cardiomyopathy; CM= cardiomyopathy; DCM= dilated cardiomyopathy; G+= pathogenic variant carrier; HCM= hypertrophic cardiomyopathy; HF= heart failure; VA= ventricular arrhythmias.

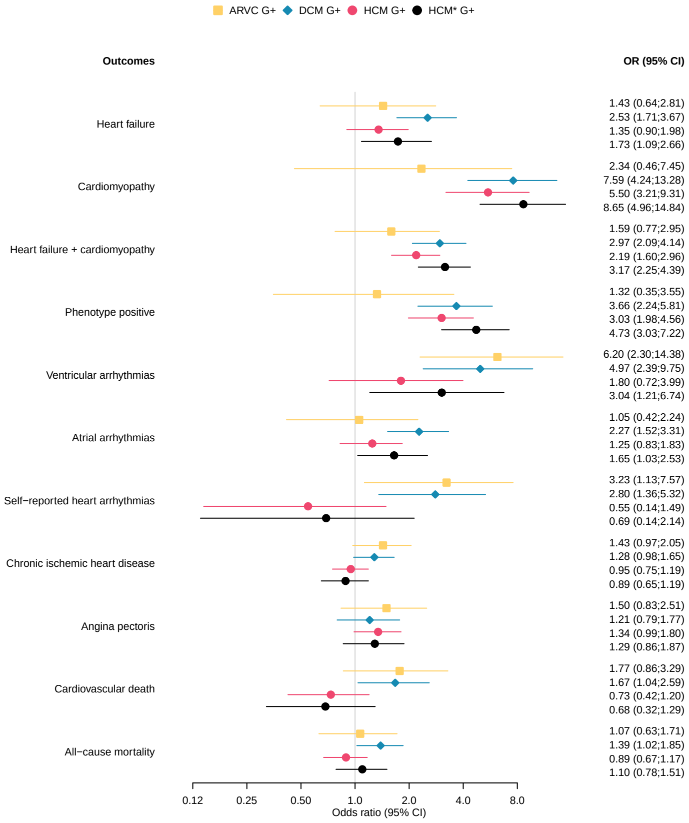

Figure II: Forest plot cardiac outcomes stratified per inherited cardiomyopathy

Odds ratios and 95% confidence intervals are given for the associations between cardiac outcomes and ARVC, DCM, or HCM pathogenic variant carriers.

Abbreviations: ARVC= arrhythmogenic right ventricular cardiomyopathy; DCM= dilated cardiomyopathy; G+= pathogenic variant carrier; HCM= hypertrophic cardiomyopathy.

* strict HCM group: HCM group after excluding carriers of the 3628-41_3628-17del MYBPC3 and the Arg278Cys 862C>T TNNT2 variant.

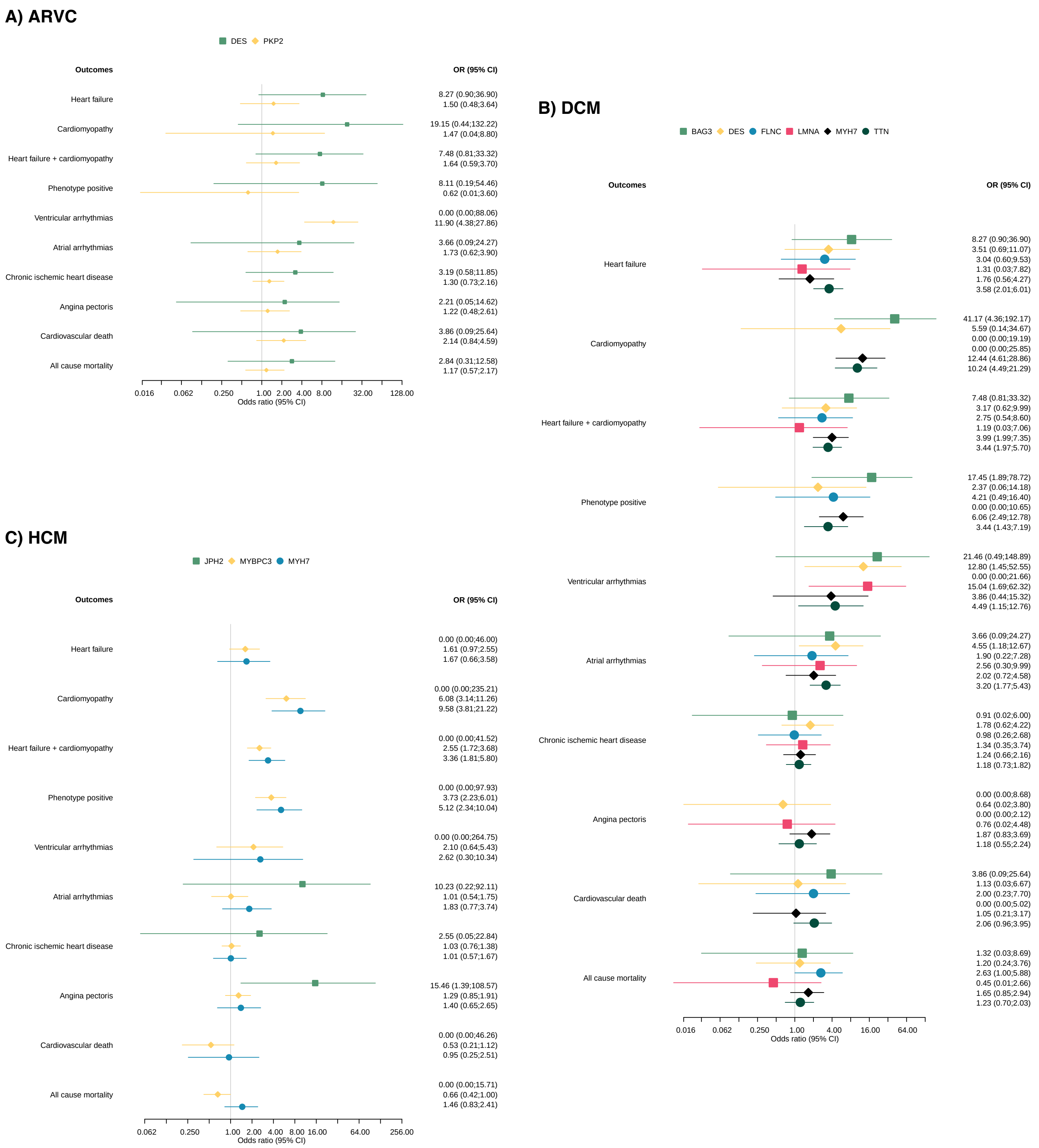

Figure III: Forest plot cardiac outcomes stratified per inherited cardiomyopathy and gene

Odds ratios and 95% confidence intervals are given for the associations between cardiac outcomes and A) ARVC, B) DCM, or C) HCM pathogenic variant carriers stratified by gene. Results are only visualized for genes with at least one significant result.

Abbreviations: ARVC= arrhythmogenic right ventricular cardiomyopathy; DCM= dilated cardiomyopathy; HCM= hypertrophic cardiomyopathy.

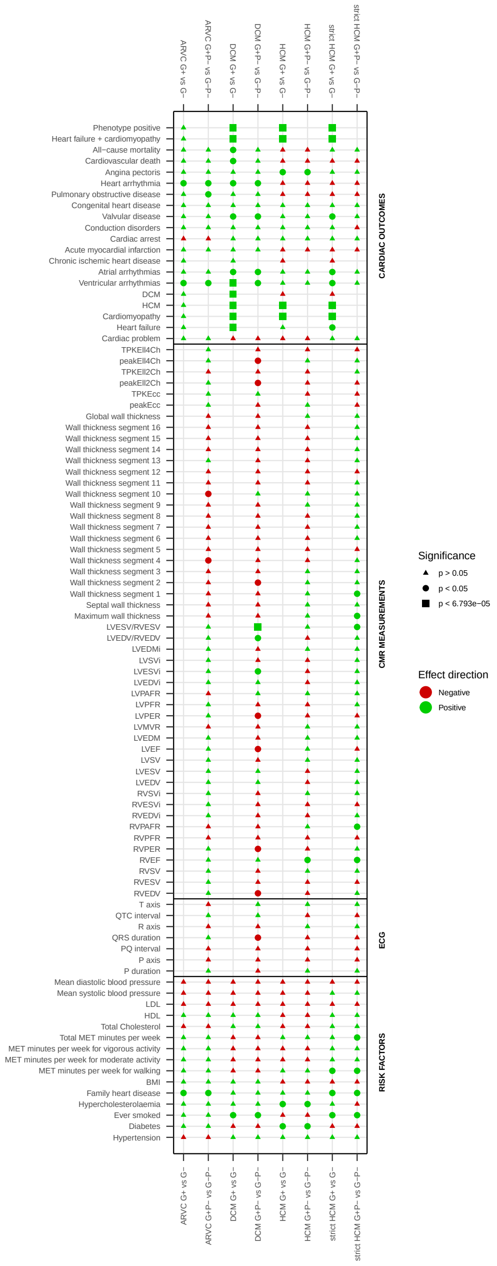

Figure IV: Matrix of all differences tested

Summary of p-values and effect direction for all performed tests in this study. The p-value is indicated by the shape of the symbol, whereas the effect direction is represented by the color. Red color indicates an odds ratio smaller than one for categorical variables and a lower mean value for the G+ compared to the G- group for continuous variables. No symbol indicates the test was not performed.

Abbreviations: ARVC= arrhythmogenic right ventricular cardiomyopathy; CMR= cardiac magnetic resonance imaging; DCM= dilated cardiomyopathy; ECG= Electrocardiography; G+= pathogenic variant carrier; HCM= hypertrophic cardiomyopathy; P-= phenotype negative individual; P+= phenotype positive individual.
